# Rigidity vs Activity: Design of Gramicidin S Analogs against Multidrug-Resistant Bacteria Based on Molecular Engineering

**DOI:** 10.1021/acs.jmedchem.5c01234

**Published:** 2025-09-30

**Authors:** Mikołaj Śleziak, Jarosław J. Panek, Tomasz Janek, Aneta Jezierska, Monika Kijewska

**Affiliations:** † 49572 University of Wrocław, Faculty of Chemistry, F. Joliot-Curie St. 14, Wrocław 50-383, Poland; ‡ 56641 Wrocław University of Environmental and Life Sciences, Faculty of Biotechnology and Food Science, Chełmońskiego St. 37, Wrocław 51-630, Poland

## Abstract

Antimicrobial peptides are a promising class of therapeutics to address antibiotic resistance; yet, their clinical use is limited by toxicity and narrow-spectrum activity. To better understand how conformational rigidity influences efficacy and safety, a series of β-sheet antimicrobial peptide analogs based on gramicidin S were designed and synthesized. Two stapled derivatives (GS_C_-FB and GS_C_-SS) and a flexible linear analog (GS-L) were prepared and evaluated. GS_C_-FB retained potent activity against Gram-positive bacteria with a significantly reduced cytotoxicity. GS-L, characterized by increased conformational flexibility, showed broader-spectrum activity, including activity against Gram-negative strains, and similarly improved safety. Circular dichroism spectroscopy revealed that all analogs displayed structural perturbations relative to native gramicidin S. Molecular dynamics simulations indicated that only flexible or moderately rigid analogs effectively interact with membrane models. These findings demonstrate that conformational rigidity is a key parameter in the design of antimicrobial peptides, enabling the optimization of antimicrobial potency while mitigating toxicity.

## Graphical abstract

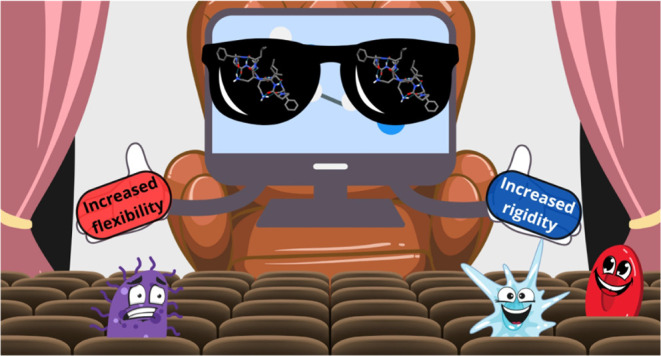

## 1 Introduction

Antibiotics are widely regarded as some of the most significant medical discoveries of the 20th century.^1^ Since their introduction, they have been hailed as “miracle drugs” for their ability to cure previously fatal infectious diseases.^2^ Moreover, their utilization has extended beyond medicine; they have also been employed in agriculture, particularly in animal husbandry and food production, as essential prophylactic agents.^1^ However, antibiotic consumption is directly associated with the emergence of antimicrobial resistance (AMR).^1,3–5^ The widespread and prolonged misuse of antibiotics over recent decades has significantly contributed to the rise of drug-resistant bacterial strains.^1,5^

Currently, AMR poses a critical and growing threat to global health. In 2019, AMR was associated with an estimated 4.95 million deaths worldwide, including 1.27 million directly attributable to resistant infections.^6^ According to the WHO, this number could rise to 10 million annually by 2050.^7^ Beyond its health impact, AMR also imposes a significant economic burden due to the costs of second-line therapies, extended hospital stays, and lost productivity.^8^ In the United States alone, treating AMR infections from six key pathogens is estimated to exceed $4.6 billion per year.^9^ Addressing AMR requires coordinated global action across healthcare, agriculture, and education. Among the necessary strategies, the development of new antimicrobial agents with a reduced resistance potential is paramount.

Promising candidates for this type of therapeutics are antimicrobial peptides (AMPs). This diverse class of compounds exhibits broad-spectrum activity against bacteria, viruses, fungi, protozoa, and even cancer cells.^10–13^ Found across all domains of life,^14^ naturally occurring AMPs, also known as host defense peptides (HDPs),^11^ play a vital role in innate immunity.^10,12,13,15^ A well-characterized AMP is gramicidin S, first discovered by Gause and Brazhinkova in 1942.^16^ It is a cyclic decapeptide with the sequence cyclo­(-Val-Orn-Leu-d-Phe-Pro-)_2_,^17^ in which the polar ornithine residues are positioned opposite the nonpolar valine and leucine residues, forming an amphiphilic structure.^18^ Gramicidin S adopts an antiparallel β-sheet conformation stabilized by two type II’ β-turns formed by Pro and d-Phe residues.^18^ Produced by the Gram-positive aerobic bacterium *Aneurinibacillus migulanus* (syn. *Bacillus brevis*),^19,20^ this cationic peptide functions as a defense mechanism against microbial competitors and displays potent activity against Gram-positive and Gram-negative bacteria, yeasts^21,22^ and viruses.^23^

Despite its potent antimicrobial activity, the clinical use of gramicidin S is restricted to topical applications due to its hemolytic and cytotoxic side effects.^17,24,25^ This toxicity arises from its mechanism of action, which poorly discriminates between microbial and mammalian membranes. Gramicidin S disrupts the lipid bilayer, increasing membrane permeability and ultimately causing cell death.^26^ To enhance its therapeutic potential, novel analogs with improved selectivity for bacteria over human membranes are urgently needed. A powerful tool in the development of new antimicrobial agents is *de novo* design, which involves creating entirely new molecular entities aimed at targeting well-defined protein-binding sites.^27^ However, this method is less applicable to membrane-active peptides like gramicidin S, whose efficacy relies on nonspecific interactions with lipid bilayers rather than specific ligand–protein recognition. In the absence of a discrete binding pocket, the structure-based design becomes challenging. As an alternative, we focused on the rational modification of gramicidin S using a peptide-stapling strategy to modulate its physicochemical properties, particularly conformational rigidity, which plays a critical role in membrane interaction, antimicrobial potency, and cytotoxicity. Molecular engineering plays a pivotal role in contemporary drug design, enabling the integration of experimental and computational approaches to develop compounds with tailored properties.

Peptide stapling is a macrocyclization technique that locks peptides into defined conformations, enhancing their stability, cell permeability, and selectivity. While widely used for α-helical peptides, typically via all-hydrocarbon stapling,^28^ its application to β-sheet AMPs remains largely unexplored. To date, only two β-sheet AMPs, BTT3 ^29^ and CA-4,^30^ have been stapled. Unlike α-helices, no clear guidelines exist for residue positioning in β-sheet stapling, and the impact of conformational rigidity on their bioactivity is poorly understood. Two main stapling strategies exist: one-component (direct side-chain bridging)^31^ and two-component (using bifunctional linkers),^32^ with the latter allowing more diverse chemical linkages without requiring unnatural amino acids.^28^

In this study, two stapled analogs of gramicidin S were designed and synthesized, differing in bridge length and consequently in their degree of conformational rigidity. Our approach involved the substitution of Leu residues with Cys residues and then—in the first stapling strategy—an introduction of a perfluoroaryl bridge via thiolate moieties to enforce structural constraints and incorporate a hydrophobic element on one side of the cyclic structure. The second stapling strategy involved the formation of a disulfide bridge between two Cys residues. To fully understand the impact of constraints on bioactivity, the stapled analogs were compared to a linear analog with reduced rigidity relative to native gramicidin S. To achieve a greater understanding of the structure–activity relationship, molecular modeling techniques were employed. We combined static and time-evolution models of gramicidin S, its two stapled analogs, and a linear variant to describe physicochemical differences in the obtained peptides. The investigations were carried out using Density Functional Theory (DFT)^33,34^ and classical molecular dynamics (MD).^35^ Both membrane and aqueous models were used to gain insight into the distinct dynamics of the peptides in hydrophobic and hydrophilic environments. The obtained results shed new light on the molecular features of gramicidin S and its stapled analogs and could be useful in the design of new β-sheet peptide analogs with improved antimicrobial activity.

## 2 Results and Discussion

### 2.1 Synthesis and Purification

Peptide stapling was employed to impose an additional level of conformational control on the already cyclized gramicidin S scaffold. We used cysteine-based side-chain stapling with variable-length bridges to precisely control conformational constraints and systematically study their impact on membrane interaction and biological activity. Perfluoroaryl- and disulfide-stapled analogs of gramicidin S were obtained following the Fmoc strategy,^36^ according to the logic of the designed experiments, by replacing two Leu residues with Cys residues in the peptide sequence. These modifications were introduced for several reasons: (i) thiol groups were required for the stapling reactions, (ii) the size of the gramicidin S backbone was preserved to minimize confounding effects on biological activity, (iii) cysteine residues enabled cyclization via native chemical ligation (NCL), and (iv) reduced lipophilicity contributed to lower cytotoxicity.^24^ The comprehensive list of all obtained peptides and intermediates is provided in Table S1.

In the first step of the synthesis, bromoacetic acid was attached to the resin, followed by the substitution of the bromine atom with Trt-cysteamine to obtain an *N*-(2-sulfanylethyl)­glycinamide moiety on the *C*-terminus. Subsequently, the amino acid chain was assembled using Boc-ornithine as the initial amino acid, possessing a Cys residue at the *N*-terminus (Scheme 1, step 1). This, along with the presence of *N*-(2-sulfanylethyl)­glycinamide at the *C*-terminus, was crucial for carrying out the cyclization step (Scheme 1, step 2). The detailed synthesis procedure was placed in Section 4.2. After the synthesis, the peptide was cleaved from the resin, simultaneously removing all acid-labile protecting groups, which was confirmed by ESI-MS analysis (Figures S1 and S2). Lyophilized cysteine-containing analog was subjected to cyclization via native chemical ligation, which was beneficial on many levels. First, the process involves a thioester, which then undergoes *trans*-thioesterification followed by an *S,N*-acyl shift.^37^ Therefore, the starting sequence does not need to contain a carboxyl group at its *C*-terminus, as this moiety does not participate in the cyclization process and is ultimately removed from the final molecule. Consequently, the synthesis can be performed by using a variety of resins with different linker types. This reaction exhibits chemoselectivity as it specifically involves the *C*-terminal *N*-(2-sulfanylethyl)­glycinamide moiety and *N*-terminal cysteine, enabling the cyclization of entirely unprotected peptides without any side reactions. Moreover, NCL operates effectively in aqueous solutions with higher peptide concentrations, presenting a more environmentally friendly approach compared to traditional head-to-tail cyclization. After a 48-h time frame, complete conversion of the linear peptide was achieved, with no remaining uncyclized precursor, which was confirmed by ESI-MS analysis (Figures S3 and S4).

**Scheme 1:**
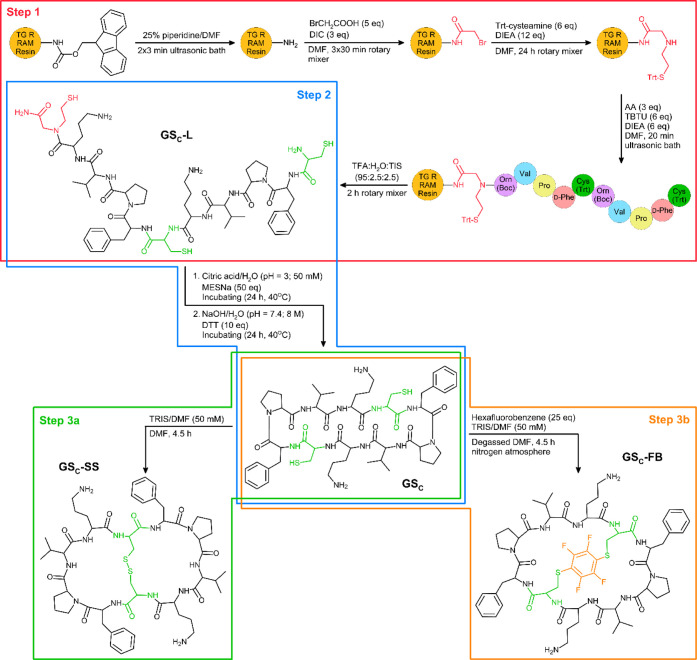
Gramicidin S Stapled Analogs (GS_C_-SS and GS_C_-FB) Synthesis Procedure^a^

a Step 1: Solid-phase synthesis of gramicidin S analog; step 2: cyclization of gramicidin S analog *via* native chemical ligation; step 3a: stapling of gramicidin S analog utilizing oxidation of −SH groups; step 3b: stapling of gramicidin S analog utilizing S_N_Ar perfluoroarylation reaction.

The cysteine-containing gramicidin S analog was subjected to stapling via two approaches. In the first approach, the bifunctional reagent hexafluorobenzene was employed. In the S_N_Ar perfluoroarylation reaction, two fluorine atoms located in the 1,4-positions of the reagent ring were substituted with thiol groups (Scheme 1, step 3b). In this reaction, anaerobic conditions were used because a competitive reaction occurred, leading to the oxidation of thiol groups into a disulfide bridge. In the second approach, the conditions for oxidizing thiol groups in the sequence to form a disulfide bridge were optimized (Scheme 1, step 3a).

GS_C_-FB was initially purified using gel filtration followed by reversed-phase high-performance liquid chromatography (RP-HPLC). In contrast, GS_C_-SS was purified only through RP-HPLC. Gel filtration was used as a preliminary purification technique for the GS_C_-FB due to its tendency to aggregate, which arises from the introduction of an additional aromatic system within its cyclic structure. The aggregation led to significant interactions with the reverse phase, which rendered purification attempts ineffective. The identity of the obtained analogs (GS_C_-FB and GS_C_-SS) was confirmed by LC-MS (Figures S5, S6, S10 and S11), ESI-MS (Figures S7, S8, S12 and S13), and ESI-MS/MS (Figures S9 and S14). An exemplary ESI-MS spectrum for the purified GS_C_-SS analog is provided in Figure 1. The spectrum shows protonated signals with *m*/*z* values of 560.2799 and 1119.5482 for the [M+2H]^2+^ and [M + H]^1+^ ions, which are consistent with the simulated isotopic distribution for the molecular formula C_54_H_78_N_12_O_10_S_2_ (Figure 2). In the fragmentation analysis, the *b* series of ions dominates, confirming the sequence of the synthesized analog (Figure 3). Full analytical data for GS_C_-FB and GS_C_-SS are provided in Tables S2 and S3, respectively.

**Figure 1:**
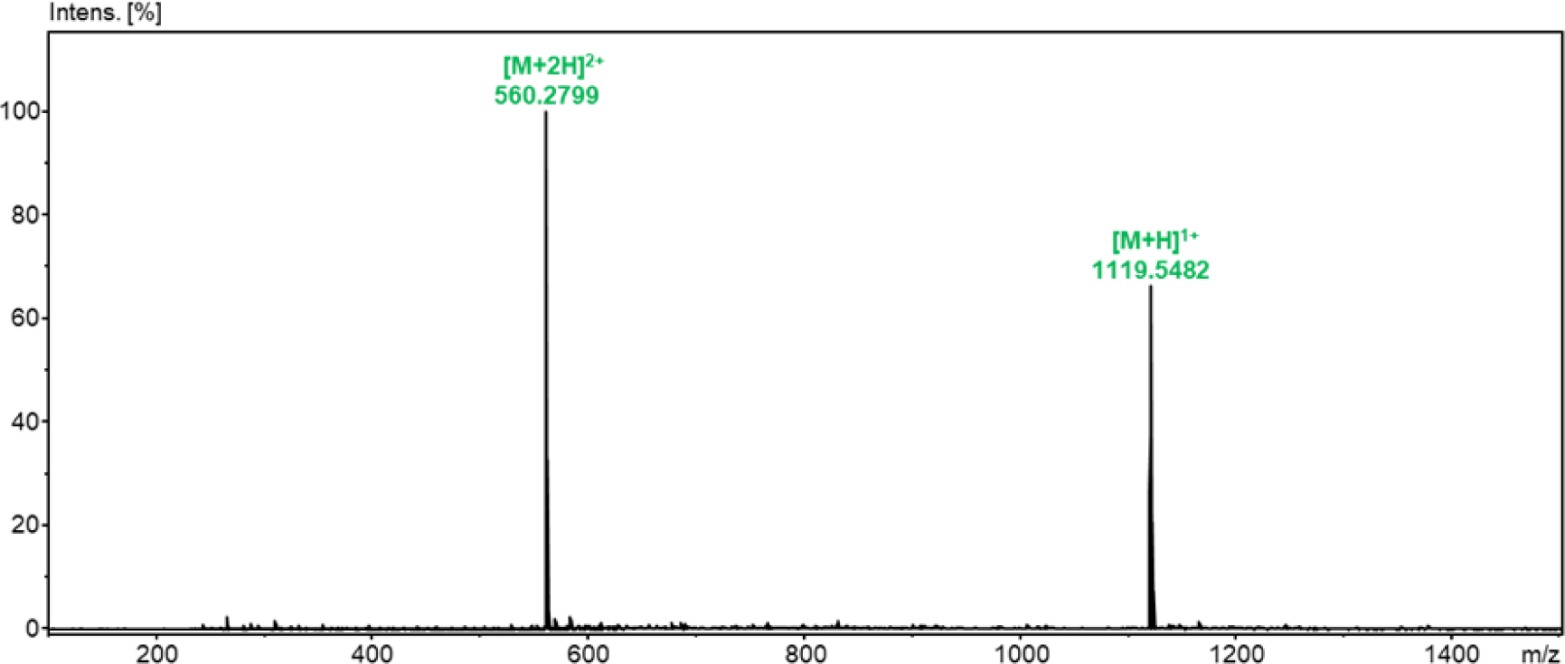
ESI-MS spectrum of GS_C_-SS (positive ion mode).

**Figure 2:**
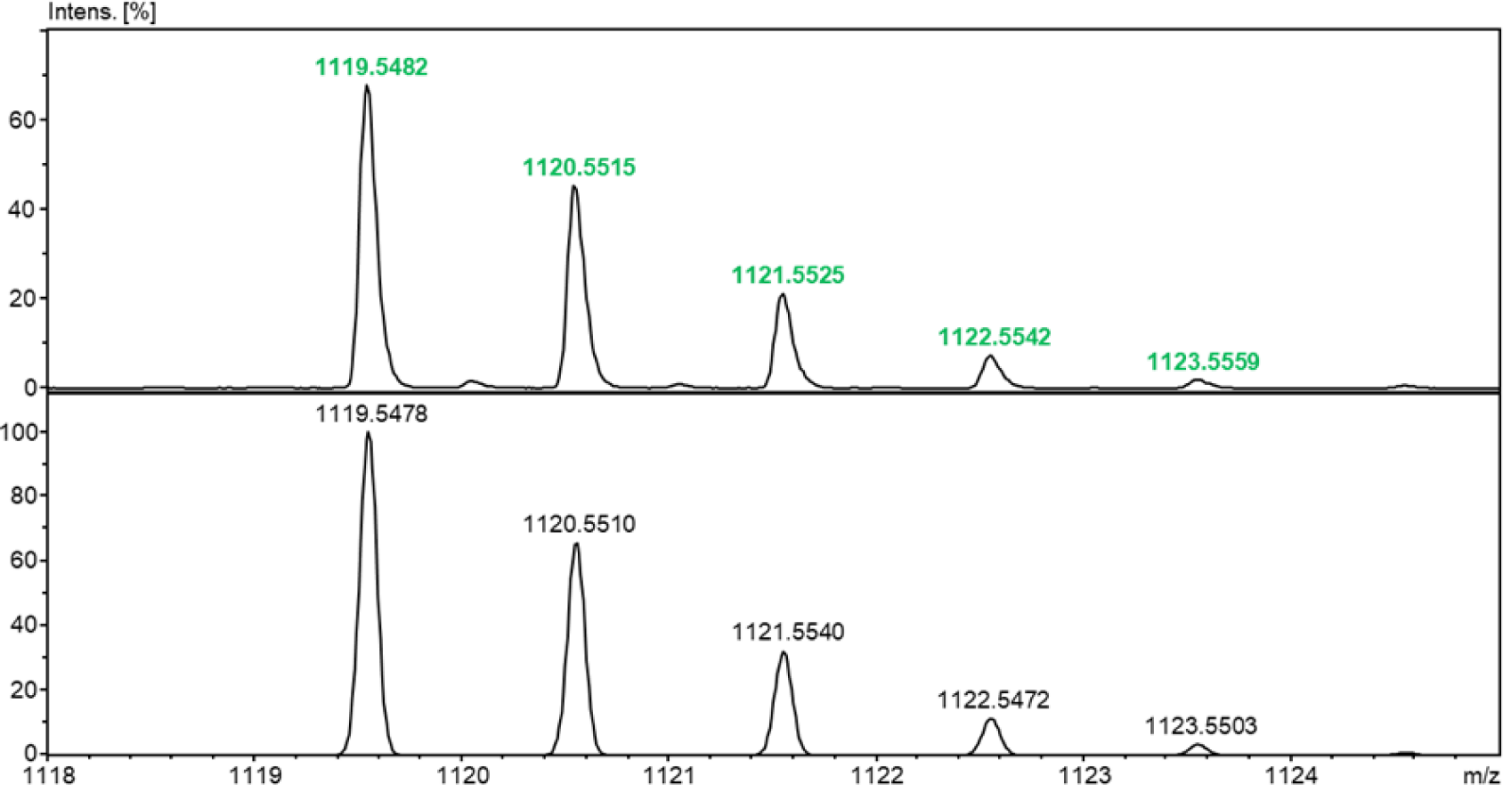
ESI-MS of GS_C_-SS in the zoom range at *m*/*z* 1118–1125 (top) and simulated for the pseudomolecular ion [M + H]^1+^ where *M* = C_54_H_78_N_12_O_10_S_2_ (bottom).

**Figure 3:**
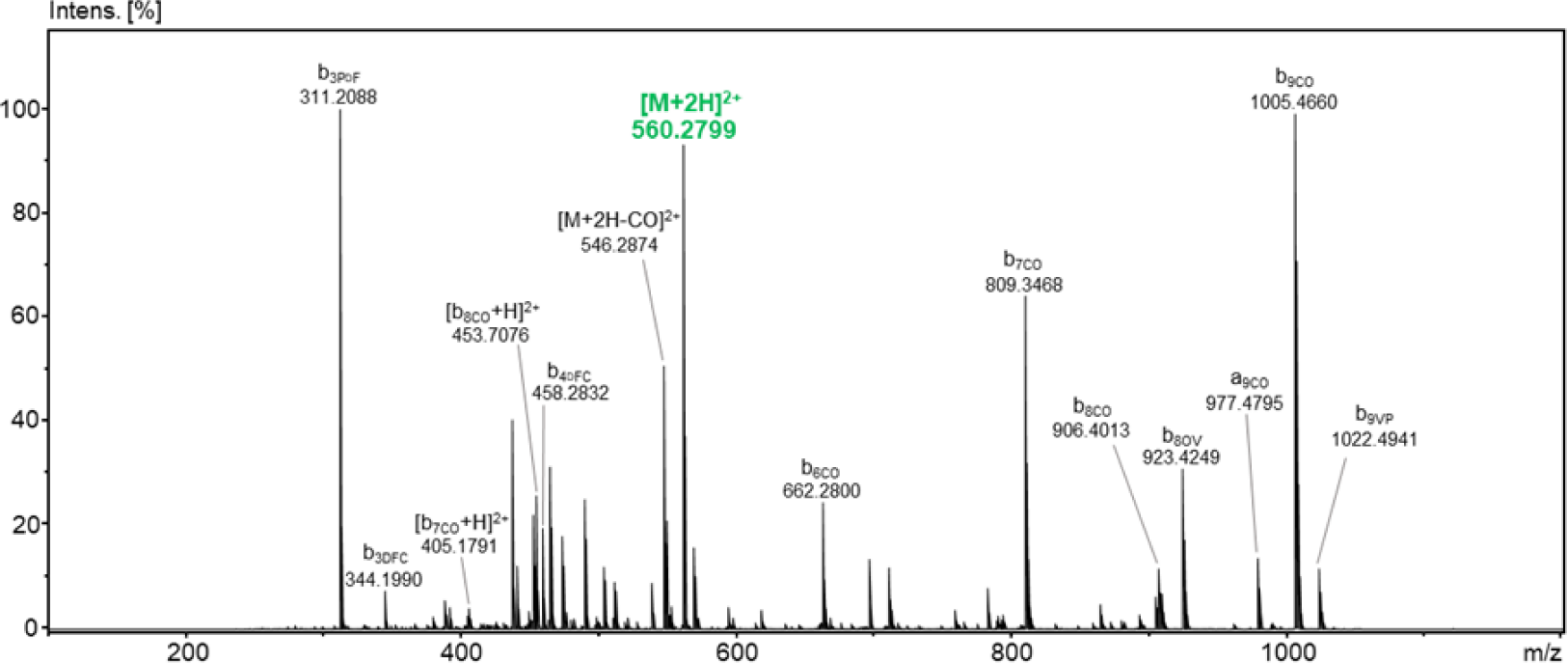
ESI-MS/MS spectrum of GS_C_-SS for the precursor ion *m*/*z* 560.2799 (positive ion mode; collision energy: 20 eV).

In order to facilitate a comparison of the biological activity at a later stage of the research project, it was necessary to obtain unmodified gramicidin S. GS solid-phase synthesis was performed using d-Phe as the starting amino acid, as it was expected to result in the highest overall yield, according to Wadhwani et al.^38^ Cyclization via a standard head-to-tail strategy requires side-chain protection of the Orn residues to avoid side reactions. Therefore, the synthesis was performed on 2-chlorotrityl chloride (2-CTC) resin, which allows the release of the peptide from the solid support under mildly acidic conditions without affecting the *tert*-butoxycarbonyl groups protecting the side chains of Orn residues (Scheme 2). After the synthesis of the whole peptide sequence, its cleavage from the resin, and identity confirmation by ESI-MS analysis (Figures S15 and S16), the cyclization reaction was carried out. The progress was monitored by ESI-MS, which resulted in an extension of the reaction time to 48 h. After this time, the postreaction mixture, containing Boc-protected gramicidin S and its linear precursor (with Pro on *N*-terminus), was deprotected under strongly acidic conditions and then separated by RP-HPLC. The identity of cyclic (GS) and linear (GS-L) products was confirmed by LC-MS (Figures S17, S18, S22 and S23), ESI-MS (Figures S19, S20, S24 and S25), and ESI-MS/MS (Figures S21 and S26). Full analytical data for GS and GS-L are provided in Tables S4 and S5, respectively.

**Scheme 2:**
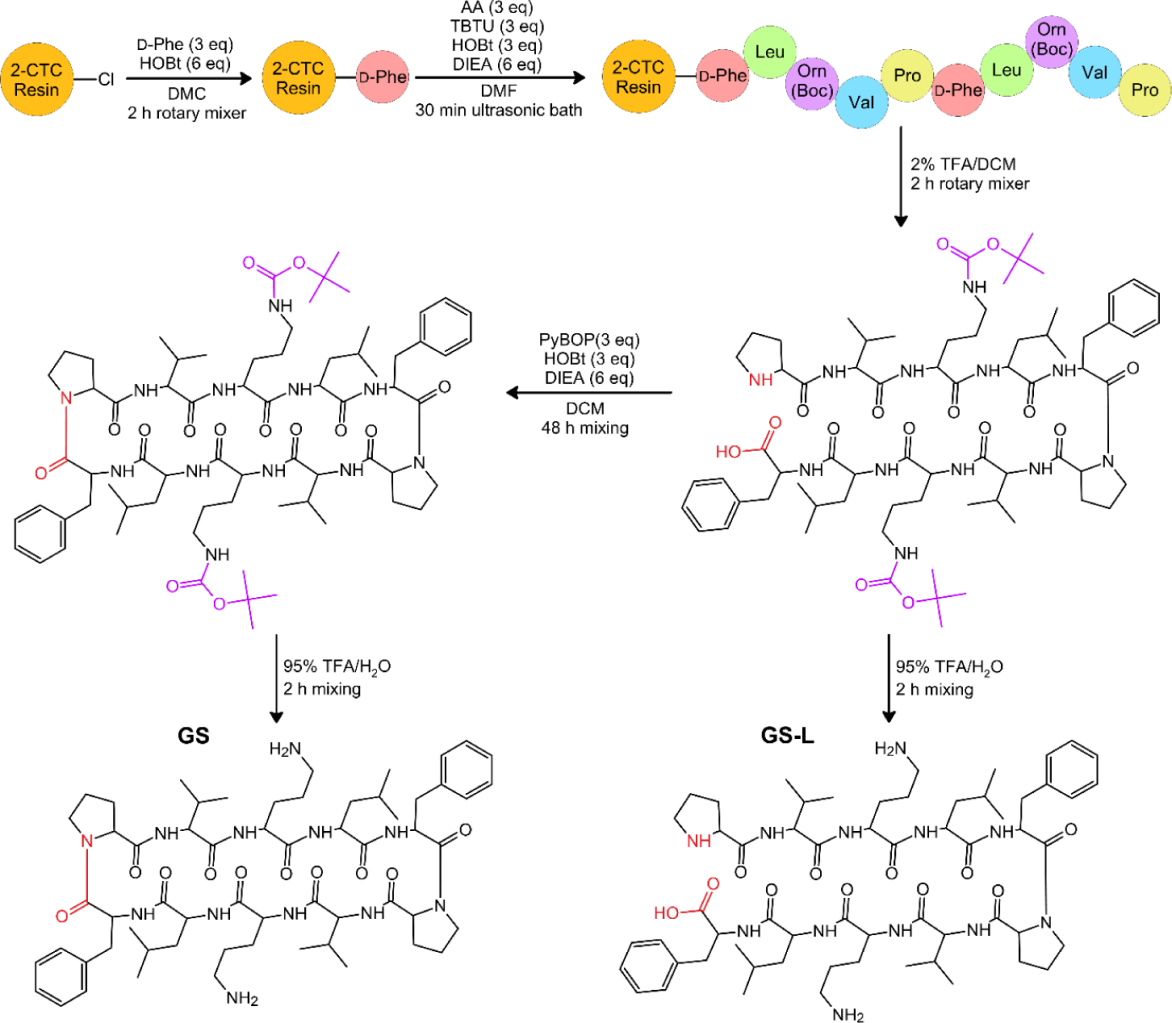
Procedure of Synthesis of the Nonmodified Gramicidin S (GS) and Its Linear Analog (GS-L)

Although the head-to-tail cyclization of gramicidin S yielded the desired product after 48 h, attempts to cyclize an analog containing Trt-cysteine residues (which remained after peptide cleavage under mildly acidic conditions) did not yield satisfactory results. Even after proceeding with the reaction for over 48 h, a significant amount of linear peptide remained (data not shown). The detailed procedure was placed in Section 4.1. We assume that the formation of a peptide bond between the *N*-terminus and *C*-terminus was limited by steric hindrance caused by the presence of two bulky trityl groups. In addition, this approach is more solvent-consuming, as it requires low concentrations of peptide (0.5 mg/mL) to avoid intermolecular reactions. Therefore, the use of NCL was a better choice for the cyclization of the cysteine-containing gramicidin S analog.

The LC-MS and HPLC-DAD analysis of the two stapled analogs (GS_C_-FB, GS_C_-SS), unmodified gramicidin S (GS) and the linear precursor (GS-L), are presented in Figures 4 and S27–S30, respectively. An introduction of the staple to the gramicidin analog results in a reduction in the hydrophobicity of these compounds compared to the unmodified peptide. The recorded retention times (RTs) for GS_C_-FB and GS_C_-SS are 9.3 and 7.6 min, respectively, compared to gramicidin S, which has an RT of 11.0 min. The linear analog has an RT of 9.3 min, which is the same as for GS_C_-FB. In the case of GS_C_-FB, next to the intense peak with an RT of 9.3 min, a less intense one with an RT of 8.2 min is also observed. However, both of these signals have identical *m*/*z* values, which correspond to the desired analog. The fragmentation spectra are also identical. This can be explained by the aggregation of this compound, which hampered purification in the reversed phase.

**Figure 4:**
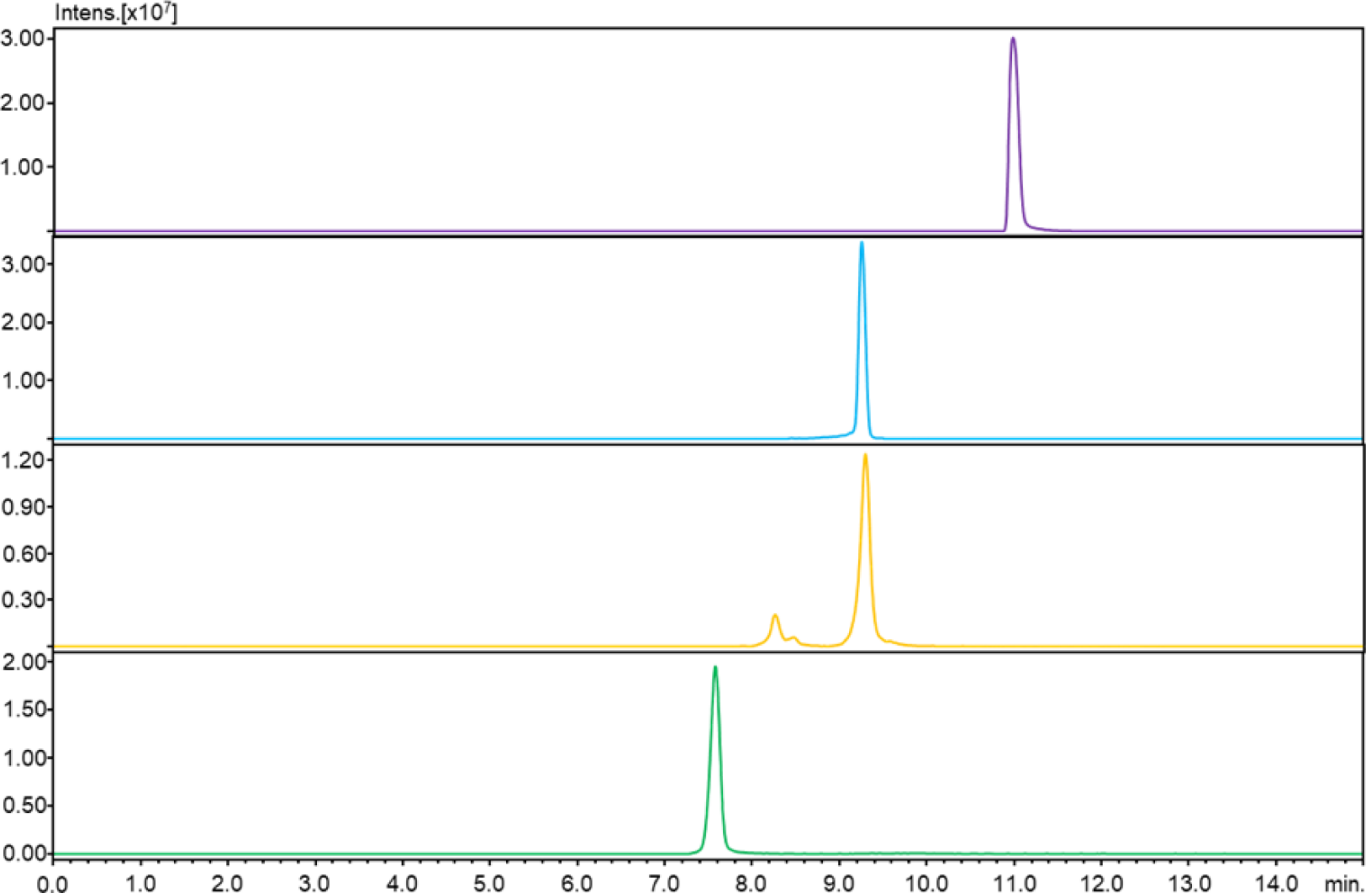
LC-MS analysis (XIC) for two stapled analogs (GS_C_-FB, GS_C_-SS), nonmodified gramicidin S (GS), and the linear precursor of gramicidin S (GS-L). Conditions: RP-Zorbax column (50× 2.1 mm, 3.5 μm); gradient elution of 0–80% B in A (A = 0.1% HCOOH in water; B = 0.1% HCOOH in MeCN) in 15 min; flow rate: 0.2 mL/min.

### 2.2 Circular Dichroism (CD) Analysis

Conformational studies of gramicidin S, GS-L, GS_C_-FB, and GS_C_-SS were carried out with CD in methanol. The choice of solvent was guided by its hydrophobic characteristics, which can mimic the interior of a membranous environment. Notably, the CD spectra of GS in methanol and sodium dodecyl sulfate (SDS) exhibit comparable minima; however, the spectrum obtained in methanol displays greater intensities, which can be linked to better stabilization of its secondary structure.^39^ This can be advantageous for detecting subtle variations in the spectral profiles. The CD spectrum of gramicidin S (Figure 5) reveals negative shoulders at wavelengths of 204.6 and 217.0 nm, which are indicative of a combination of β-sheet and β-turn motifs as described in the existing literature.^39,40^ A quantitative comparison of the CD spectra was performed to assess the relationship between the conformational variations and biological activity (Table 1). In comparison to native GS, all analogs exhibited notable spectral changes, as reflected by the progressive decrease in ellipticity in the order: GS_C_-FB, GS-L, and GS_C_-SS. The implemented modifications most prominently affected the spectral region associated with β-sheet structures (215–225 nm), whereas the β-turn region (200–210 nm) was less impacted. In all three analogs—GS-L, GS_C_-SS, and GS_C_-FB—the characteristic minima of β-turns remained clearly visible but were accompanied by a slight blue shift (from −0.2 to −1.6 nm) and a reduction in ellipticity. These observations suggest that the β-turns are largely preserved although somewhat distorted. Measurements of dihedral angles in DFT-optimized models of all peptides (Table S6) have confirmed the presence of type II’ turns, which aligns with the literature data.^41^ In contrast, changes in the β-sheet region were more pronounced. While native GS displayed a less intense minimum in this region, its analogs showed a substantial reduction in ellipticity, particularly GS_C_-SS, suggesting a diminished contribution of ordered β-sheet structures. Notably, GS_C_-SS also exhibited a red shift (+0.8 nm) in contrast to the blue shifts observed for GS-L (−4.6 nm) and GS_C_-FB (−6.8 nm). All of these spectral changes align with the observed reduction in antimicrobial activity, suggesting that peptide stapling not only increases conformational rigidity but also affects secondary structure in a bridge-length-dependent manner.

**Table 1:** Qualitative CD Parameters of GS and GS Analogs

	**β-turn region**			**β-sheet region**		
**Compound**	**λ [nm]**	**θ [deg**·**cm** ^**2**^·**dmol** ^**–1**^]	**Δλ [nm]**	**λ [nm]**	**θ [deg**·**cm** ^**2**^·**dmol** ^**–1**^]	**Δλ [nm]**
**GS**	204.6	–238499.72	N/A	217.0	–206471.69	N/A
**GS-L**	204.4	–139531.98	–0.2	212.4	–118007.41	–4.6
**GS**_**C**_ **-FB**	202.8	–184663.25	–1.6	210.2	–158099.39	–6.8
**GS**_**C**_ **-SS**	203.8	–82993.20	–0.8	217.8	–52469.30	0.8

**Figure 5:**
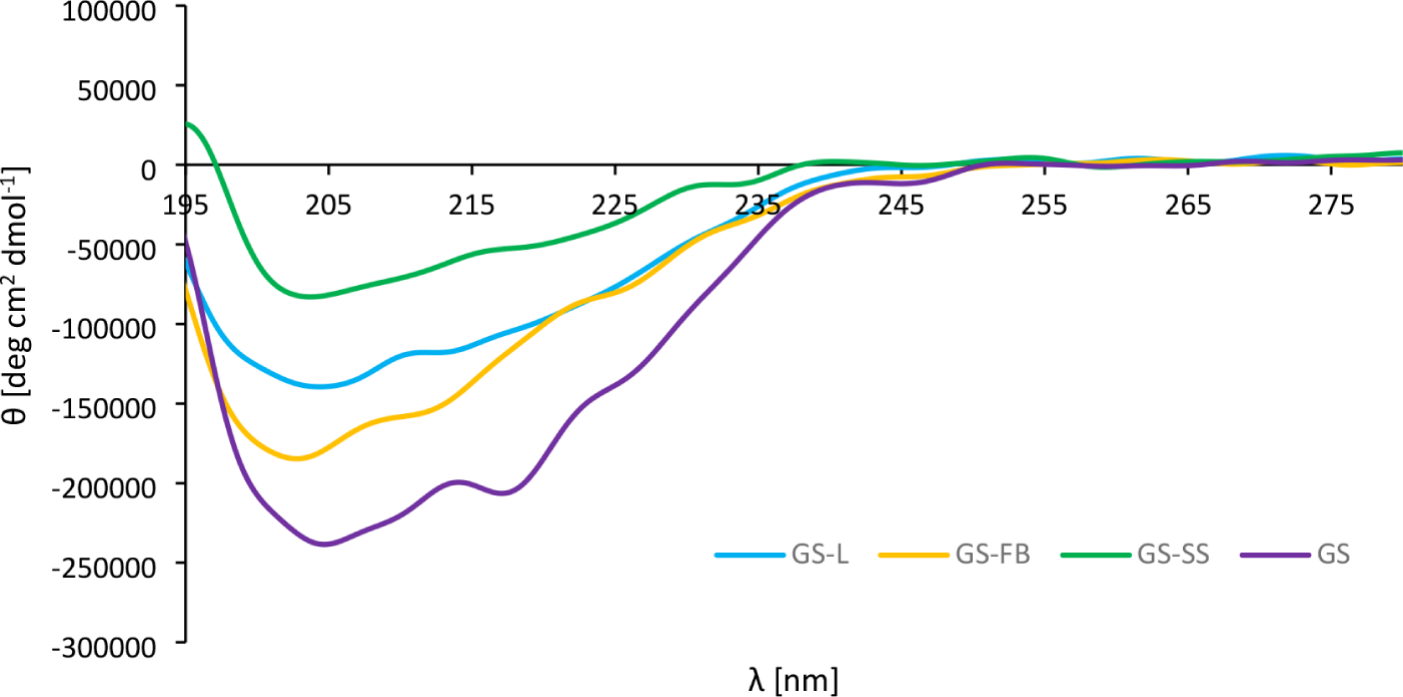
Circular dichroism (CD) spectra of GS, GS-L, GS_C_-FB, and GS_C_-SS.

GS_C_-SS with a shorter staple showed the most significant disruption in both the β-sheet and β-turn parts of the molecule as well as the lowest retention time among the analyzed compounds (LC-MS). When the CD spectra of the peptides reflect changes in the secondary structure, the RTs observed in RP-HPLC correspond to alterations in hydrophobicity. These correlations, considered together, can be associated with the structural changes in the peptide backbone and the reorganization of the side chains, which may have significant implications for biological activity. The CD spectral pattern of GS-L and GS_C_-FB is similar between those analogs but slightly distinct from the one obtained for GS, thus showing structural changes that led to altered biological activity (discussed in detail below). The pronounced disruption of the secondary structure of GS_C_-SS in solution, as confirmed by its CD spectrum, combined with reduced hydrophobicity and increased conformational rigidity, resulted in a complete loss of biological activity.

### 2.3 Antibacterial Activity

Infections caused by pathogens can result in both acute and chronic diseases. Frequently encountered multidrug-resistant pathogens responsible for hospital-acquired infections include Gram-positive bacteria such as *Staphylococcus aureus*, *Staphylococcus epidermidis*, and *Enterococcus faecalis*, as well as Gram-negative bacteria like *Pseudomonas aeruginosa*, *Escherichia coli*, *Proteus mirabilis*, and *Klebsiella pneumoniae*.^42^ Gram-negative bacteria tend to exhibit higher resistance to antibiotics due to their outer membrane, which limits drug permeability.^43^ The antimicrobial properties of synthetic GS and its analogs GS-L, GS_C_-FB, and GS_C_-SS were evaluated against both Gram-positive and Gram-negative bacteria using a standard broth microdilution assay. The tested bacterial strains included *S. aureus*, *E. faecalis*, *P. aeruginosa*, and *E. coli*. The minimum inhibitory concentration (MIC) for each compound and strain was measured (Table 2). In comparison to GS, the average MIC for Gram-positive bacteria increased by 4 to 8 times for GS-L and GS_C_-FB, while the GS_C_-SS analog showed no activity. The stapled GS_C_-FB analog showed selectivity only toward Gram-positive bacteria, while the linear analog exhibited activity against both Gram-positive and Gram-negative bacteria, with a higher MIC value for the Gram-negative strains.

**Table 2:** *In Vitro* Antibacterial Activity of Peptides Determined as a Minimal Inhibitory Concentration (MIC) (μg/mL)

	**MIC [μg/mL]**			
**Strain**	**GS**	**GS-L**	**GS**_**C**_ **-FB**	**GS**_**C**_ **-SS**
*S. aureus* ATCC 25923	8	64	32	>128
*E. faecalis* ATCC 29212	16	64	32	>128
*P. aeruginosa* ATCC 15422	32	64	>128	>128
*E. coli* ATCC 25922	64	128	>128	>128

### 2.4 Hemolytic Activity

The hemolytic activity of each compound was also evaluated. The minimum concentration needed to cause 50% lysis of sheep red blood cells (HC_50_) was determined (Table 3). The HC_50_ value for synthetic GS was 16 μg/mL, which is consistent with previously reported data.^44^ The HC_50_ values indicate that GS modifications also influenced the hemolytic activity. Replacing the Leu residues with Cys residues and introducing stiffening in the form of an aromatic system in GS_C_-FB led to an increase in HC_50_ values by up to 4 times compared to GS, suggesting that the hemolytic effects of this analog were reduced. Analogous findings were noted for the linear precursor, where an 8-fold reduction in hemolysis was observed. However, the MIC required to achieve antimicrobial activity for these peptides was only 2–8 times higher, depending on the bacterial strain, compared to the unmodified GS. This suggests that a better balance between safety and antibacterial potency was achieved. On the other hand, GS_C_-SS once again exhibited a total lack of activity.

**Table 3:** Hemolytic Activity of GS and GS Analogs

**Compound**	**HC**_**50**_ **[μg/mL]**
**GS**	16
**GS-L**	128
**GS**_**C**_ **-FB**	64
**GS**_**C**_ **-SS**	>128

### 2.5 Cytotoxicity Assay

The cytotoxicity of the peptides in Normal Human Dermal Fibroblast (NHDF) cells was evaluated by a standard MTT assay, which demonstrates active energization of cells and is conventionally used to measure cell viability. GS, a well-known cytolytic peptide, induced 100% cytotoxicity at 64 μg/mL, whereas GS_C_-FB, GS-L, and GS_C_-SS at 64 μg/mL were nontoxic to NHDF cells, resulting in nearly 70, 85, and 90% cell viability, respectively (Figure 6). GS_C_-FB and GC-L did not show cytotoxicity or hemolytic activity over the entire range of concentrations in which they exhibit antibacterial activity. In contrast, GS in the case of *P. aeruginosa* and *E. coli* is cytotoxic and causes hemolysis at the minimal inhibitory concentrations. Additionally, they show a hemolytic effect at the MIC for *E. faecalis*. Cytotoxicity increased in the order: GS_C_-SS < GS-L < GS_C_-FB < GS. While this trend partly correlates with increasing hydrophobicity, as indicated by RT in RP-HPLC (Figure 5) for GS and GS_C_-SS, it does not fully explain the differences observed between GS_C_-FB and GS-L, which exhibit identical RTs but differ in cytotoxicity.

**Figure 6:**
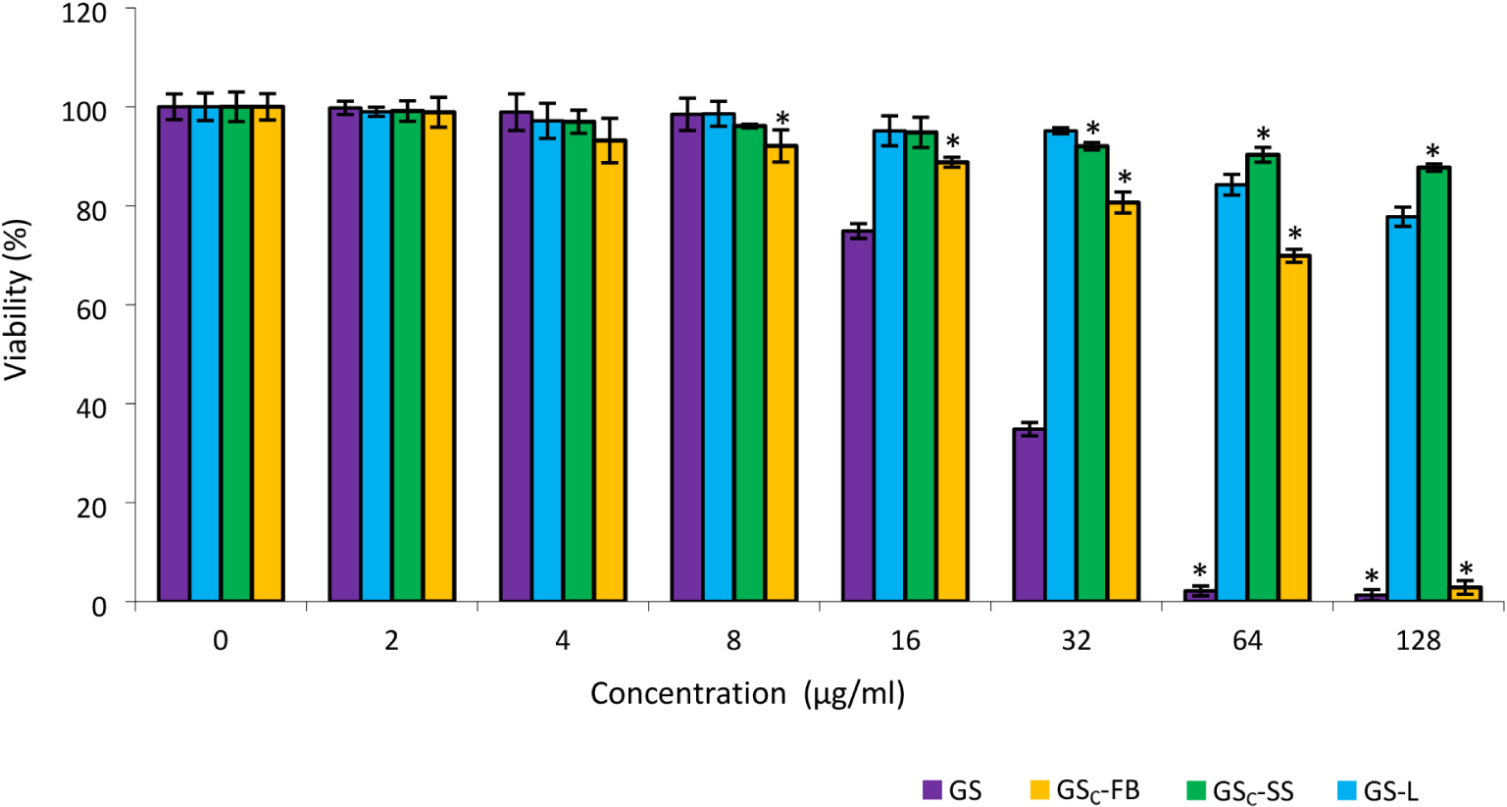
MTT assay of GS and GS derivatives’ cytotoxicity on NHDF cells. Cell lines were exposed to GS and GS derivatives (0–128 μg/mL) for 24 h, followed by an MTT incubation and viability determination via spectrophotometry at 570 nm. The bars represent the means ± SD of three independent experiments. *p < 0.05.

### 2.6 Molecular Modeling

Various theoretical chemistry approaches were employed to provide a comprehensive characterization of the new peptides. A two-step strategy was employed for this purpose. It consisted of static, DFT-based geometric and electronic structure studies, as well as time-evolution—classical molecular dynamics. This methodological approach was dictated by the novelty and challenges associated with the project. The relevant results from the MD studies on the interactions of the stapled peptides with biological membrane models, as well as the discussion of their MD behavior in aqueous solution, are presented in the main text. Additional data, including density functional theory (DFT) results and supplementary MD findings in aqueous solution, are provided in the Supporting Information.

We assumed that the linear peptide GS-L would exhibit greater conformational flexibility in comparison to the cyclic GS. Similarly, it follows that the rigidity of GS_C_-FB would be stronger due to the presence of an additional covalent bridge, and GS_C_-SS would demonstrate even greater stiffening owing to the shorter staple. Our assumptions concerning the rigidity of gramicidin S analogs have been validated with time-evolution studies based on classical MD studies, which were performed for two kinds of models: the aqueous solution and within the membrane environment. The first analysis conducted was the estimation of the root mean square deviation (RMSD), which provided information on the overall stability of the studied peptides as well as their conformational diversity. The RMSD analysis (Figure 7) indicated that among all of the investigated peptides, GS-L, which lacks a cyclic structure, displayed the greatest deviation from its initial position, regardless of the surrounding environment. Notably, within the membrane environment, GS-L showed fluctuations around a lower value. This behavior suggests that GS-L can adapt its conformational changes to better fit the surrounding conditions. Stapled analogs displayed lower RMSD values in water compared to unmodified GS, with GS_C_-SS showing the strongest similarity to the reference structures (geometries taken from the static DFT simulations, Figures S31–S34). In the membrane, GS and mildly stiffened GS_C_-FB exhibited lower values and fluctuations than in water, approaching values observed for the most rigid GS_C_-SS.

**Figure 7:**
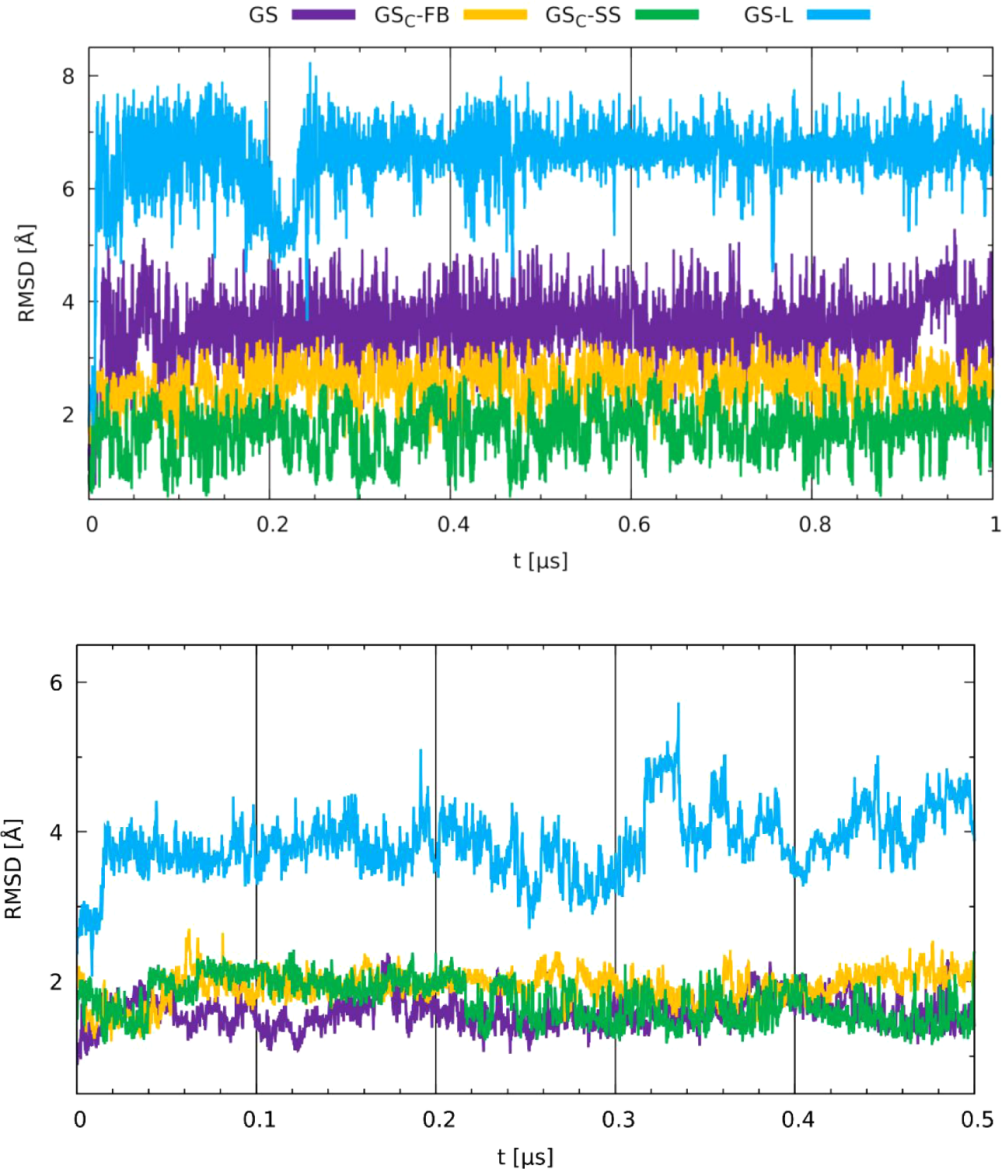
Root mean square deviation (RMSD) obtained for GS, GS-L, GS_C_-FB, and GS_C_-FB in water (top) and the membrane environment (bottom).

The radius of gyration (RGYR) analysis in the membrane (Figure 8 bottom) shows that the largest fluctuations were observed for the GS-L analog, followed by the original gramicidin, GS. The GS_C_-SS staple, after an initial period of approximately 200 ns, managed to expand its structure, which—considering the following analyses (e.g., center of mass distances)—can relate to its diminished ability to embed within the membrane. The GS and GS_C_-FB analogs also stabilized their RGYR feature after about 200 ns, indicating that this is the time required for the gramicidin analog to establish a preferable position in the membrane. As shown in Figure 8 top panel, the most significant fluctuations in water were also observed for the GS-L analog. In the case of other peptides, the values of RGYR were comparable (between 5.5 Å and 6.5 Å), suggesting that cyclic peptides exhibit similar properties in water when RGYR is taken as a descriptor of the molecular shape.

**Figure 8:**
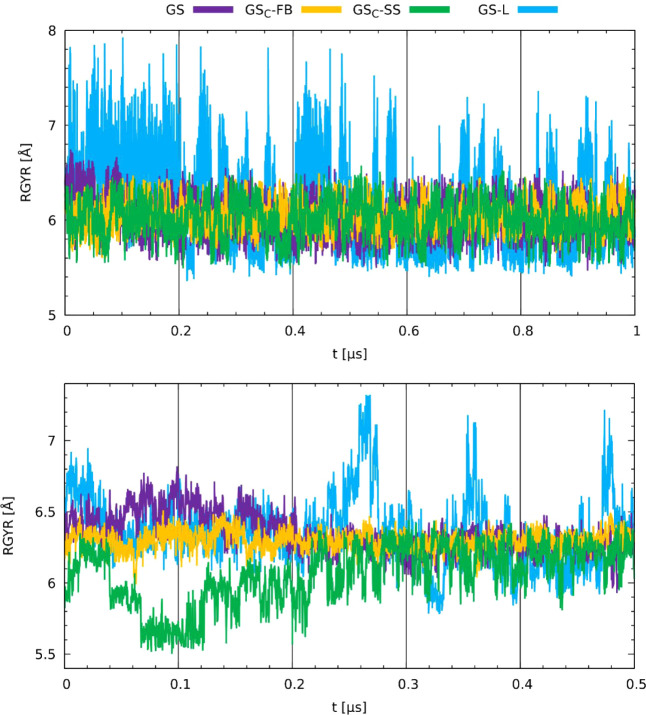
Radius of gyration (RGYR) calculated for GS, GS-L, GS_C_-FB, and GS_C_-FB in water (top) and the membrane environment (bottom).

The solvent-accessible surface area of a peptide (SASA) is another structural parameter, which—in the case of the amphiphilic environment of the membrane—has different application than its routine uses in explicit solvation models. In our case, the SASA analysis was applied to reveal structural changes that could expose different fragments of the studied peptides to the external factors. Figure 9 shows that the GS_C_-SS analog is again much different from the other peptides due to its more rigid nature, while the GS-L peptide is the most exposed. However, it must be noted that—in distinction from the RGYR parameter—the SASA does not exhibit dramatical fluctuations, so none of the peptides underwent conformational changes that would expose or hide polar groups.

**Figure 9:**
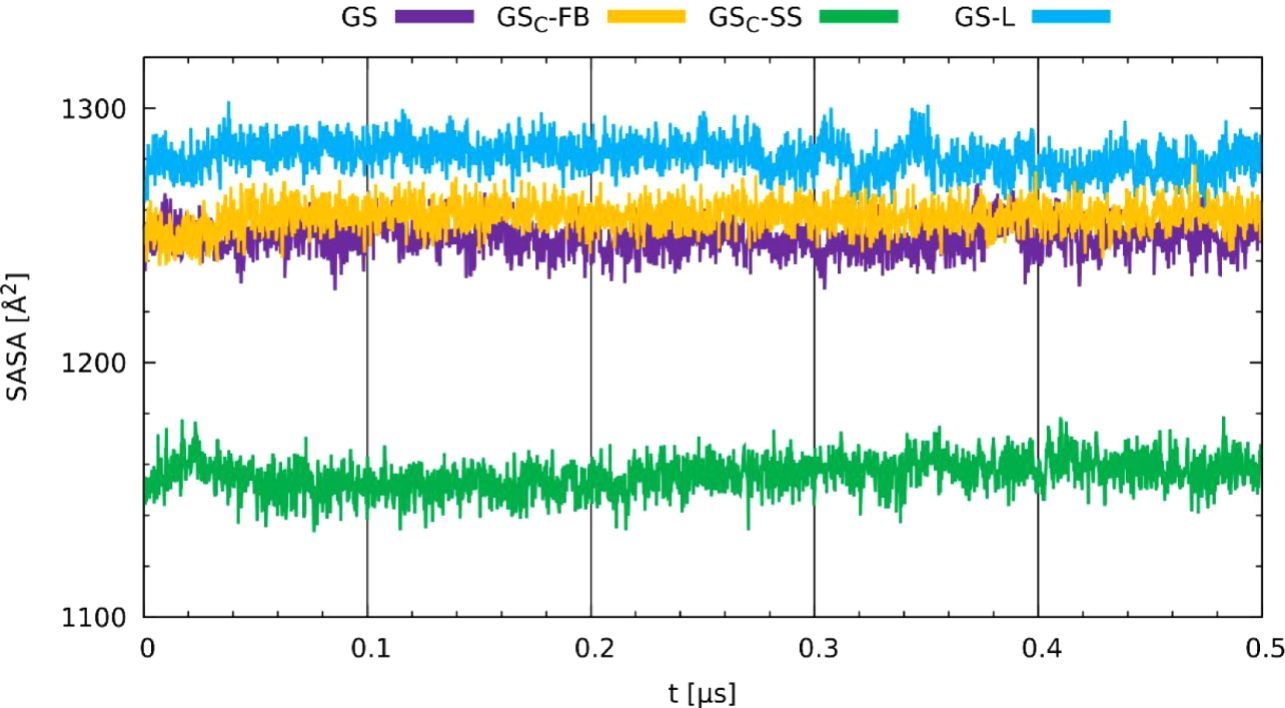
Time evolution of the solvent-accessible surface area (SASA) obtained for GS, GS-L, GS_C_-FB, and GS_C_-SS in membrane environment.

To further assess the structural rigidity and overall shape parameters, the distances between α-carbons of selected residues along the obtained trajectories were predicted (Scheme 3). The interactions between the corresponding pairs of Orn residues in the membrane environment (Figure 10 bottom) are most constant for the GS and GS_C_-SS peptides. Moreover , those distances in this pair of peptides remain the same throughout the entire simulation, fluctuating around 4 Å. This value is also preserved in the water environment for GS_C_-SS but not for GS, indicating that the unmodified peptide was highly compressed by the membrane to reach values observed in the most rigid analog (Figure 10 top). For the linear GS-L peptide, the absence of a cyclic structure allows for large amplitude fluctuations in the Orn–Orn contacts in both environments. Interestingly, the GS_C_-FB stapled peptide expands this contact at ca. 40 ns of the simulation in the membrane and ca. 100 ns in the water, and then maintains this relatively longer distance until the end of the simulation, with also larger amplitude than for the GS and GS_C_-SS. This expansion might relate to the behavior observed in the water environment (discussed in detail below), where GS_C_-FB exhibited larger water retention in the formed pocket. Notably, both stapled peptides exhibited similar spatial distances along the obtained trajectories, regardless of the surrounding environment, with GS_C_-SS maintaining an approximate distance of 4 Å and GS_C_-FB at approximately 7 Å. This observation suggests that the incorporation of stapling imparts structural rigidity, thereby reducing the conformational susceptibility of the peptides to their environment.

**Scheme 3:**
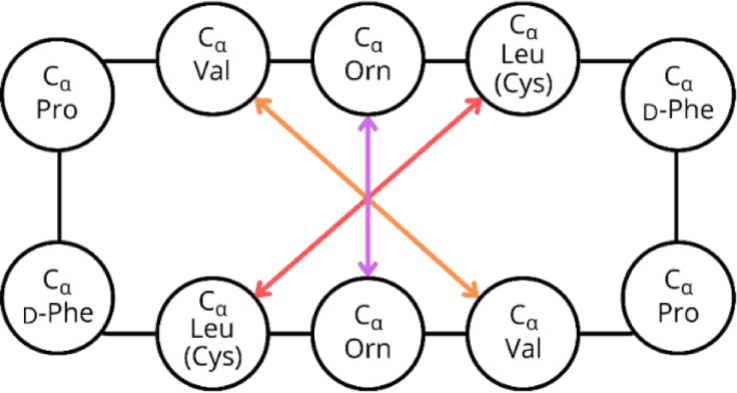
Selected Residues for Calculations of the Distance Evolution between α-Carbons Along the Obtained MD Trajectories (in Both the Membrane Model and the Aqueous Environment)

**Figure 10:**
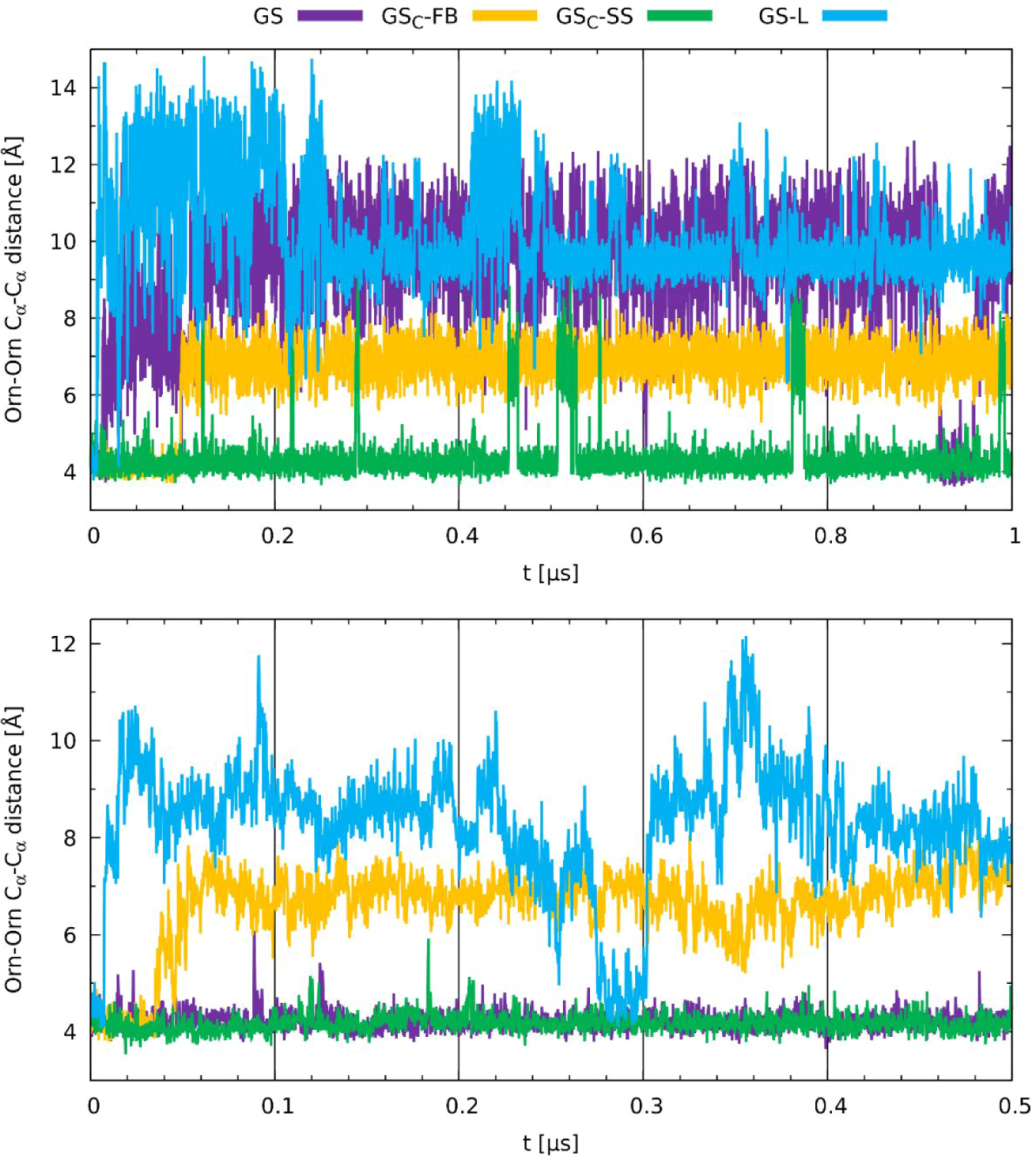
Time evolution of distances of α-carbons between Orn residues obtained for GS, GS-L, GS_C_-FB, and GS_C_-SS in water (top) and the membrane environment (bottom).

In Figure 11, the Leu–Leu (Cys–Cys in modified peptides) distance time evolution is presented. A similar pattern emerges, in which the GS_C_-SS structure preserves the relevant interatomic distances most closely throughout the simulation; in contrast, the linear peptide exhibits the greatest conformational flexibility. The behavior of Leu–Leu distances in GS within the membrane environment is similar to that observed in previous analysis, indicating that the peptide adopts a more compressed structure compared to its conformation in water, with values approaching those seen in the most rigid analog, GS_C_-SS. Consistent distances were again observed in the stapled peptides across both environments, with values fluctuating around 8.5 Å for GS_C_-FB and approximately 6 Å for GS_C_-SS. Notably, these measurements correspond to the residues involved in staple formation, thus reflecting the effective length of the staple.

**Figure 11:**
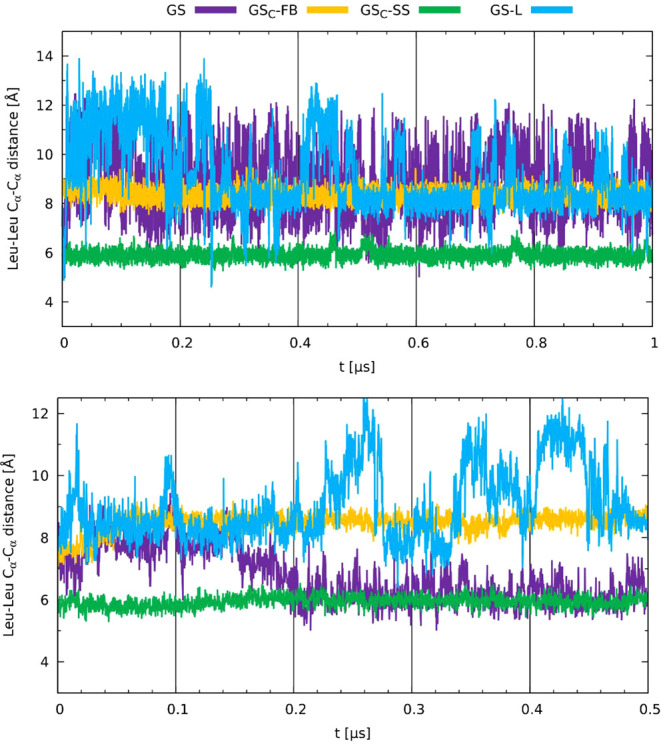
Time evolution of distances of α-carbons between Leu residues for GS, GS-L, as well as between Cys residues for GS_C_-FB and GS_C_-SS in the water (top) and the membrane environment (bottom).

The last analysis of the shape parameter concerns the Val–Val distance (Figure 12). Here, we observed the smallest differences among all four analogs. This can be attributed to the position of valine residues, which are the most distant from the staples among all measured residues; thus, the flexibility of those regions starts to converge. The analysis in both environments indicated that the GS and GS_C_-SS are the most stable, while the GS_C_-FB is also relatively rigid after the initial structural expansion at ca. 40 ns. The GS-L peptide shows not only the largest amplitudes but also the overall closest Val–Val distances, which could be attributed to the zwitterionic ends being able to maintain close contacts but also prone to detachment under the influence of the environment.

**Figure 12:**
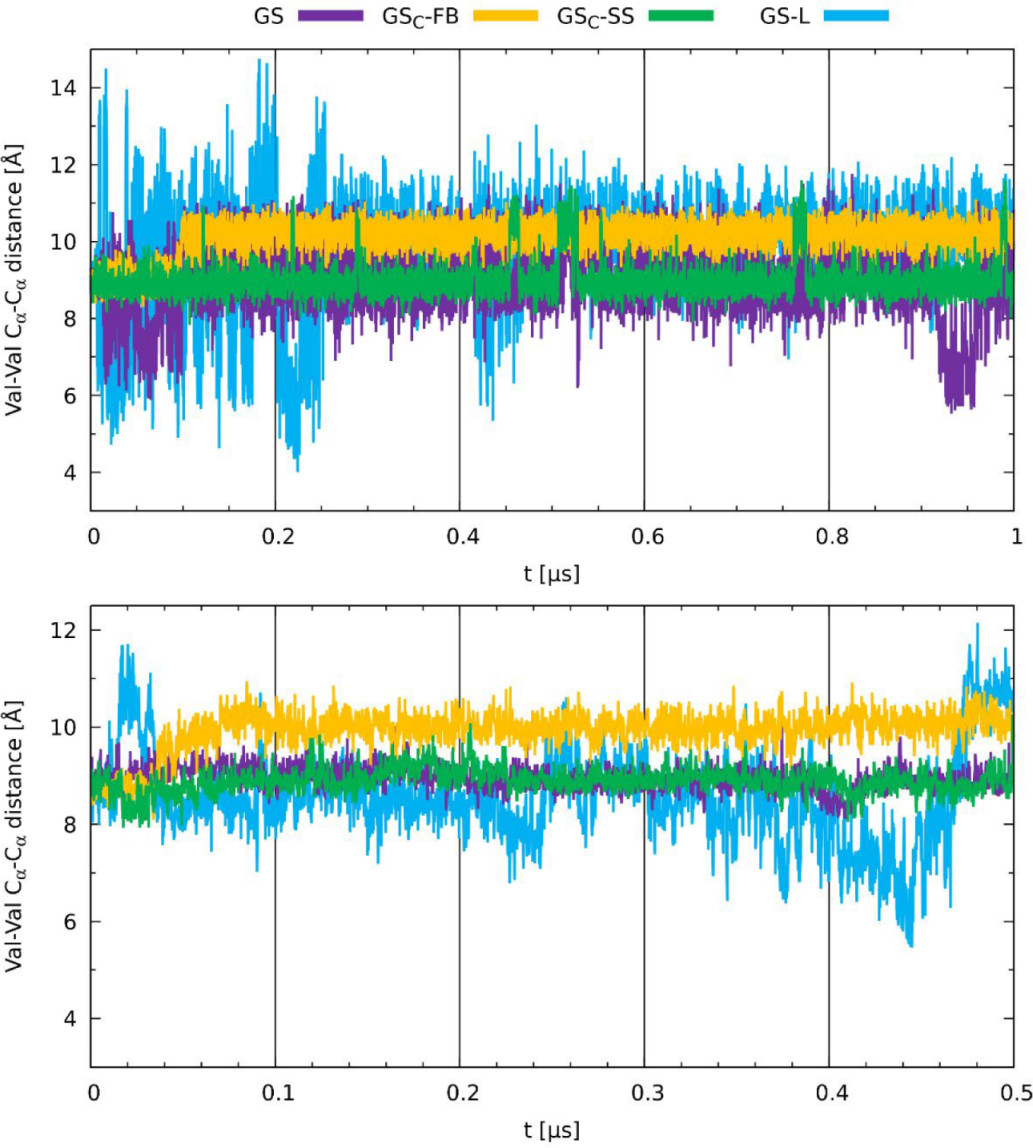
Time evolution of distances of α-carbons between Val residues obtained for GS, GS-L, GS_C_-FB, and GS_C_-SS in water (top) and the membrane environment (bottom).

Throughout the entire duration of these dynamics, all distances for GS_C_-SS were consistently maintained, indicating the presence of structural constraints. Moreover, their values were usually the lowest among all the studied peptides. Similarly, the distances between α-carbons of residues in GS_C_-FB were characterized by small deviations during the simulation time, but with higher values compared to GS_C_-SS. All contacts within stapled peptides remained consistent across both environments. This observation suggests that stapling enhances the rigidity of the secondary structure, and shorter staples contribute to greater rigidity. Once the peptide adopts this stabilized conformation, it becomes significantly challenging for the peptide to alter its structure, even in a dense phospholipid bilayer. The highest variation in α-carbon distances was observed in the most flexible linear analog. The unmodified GS demonstrated a higher degree of structural variability compared to the stapled analogs. A noteworthy observation is the consistent proximity of the distances between the Orn, Val, and Leu residues in GS in water, all fluctuating around approximately 9.0 Å. This phenomenon suggests that in a polar environment, gramicidin S adopts an expanded conformation. This conformation demonstrated the capability to establish hydrogen bonds with a maximum of three water molecules inside the cyclic structure, thereby displacing intramolecular bonds crucial for maintaining the antiparallel β-sheet structure (Figure S45). In the case of GS_C_-FB, a cavity was formed, allowing only one water molecule at a time to form an intermolecular hydrogen bond (Figure S46). This single water molecule was able to occupy the cavity for as long as 7.58% of the molecular dynamics’ simulation time (75.8 ns), while for the GS the water molecules’ retention was less than 1% of the MD production run (<10 ns). On the contrary, GS_C_-SS no longer possessed the capability to engage in hydrogen bonding with water molecules within the cyclic structure—the net result is visible in the SASA analysis in water (Figure S43). We noted that the GS_C_-SS in water exhibits the lowest Orn–Orn and Cys–Cys distances among the studied cyclic peptides, but the Val–Val distances are similar across all peptides. This indicates that the structure of GS_C_-SS is the most elongated and remains so through the majority of the MD simulation. The stapled analogs showed a conformation more similar to that obtained from DFT calculations. Regarding the behavior of GS in the membrane environment, it was observed that GS compressed its inter-residual distances to values closely resembling those calculated for GS_C_-SS. This suggests that GS possesses a flexible structure capable of adapting to changing environmental conditions. The membrane-associated conformation is likely bioactive, implying that the disulfide bridge in GS_C_-SS may stabilize those distances characteristic of the bioactive form of gramicidin S. However, inter-residual distances represent only one aspect of the secondary structure. Taken together with CD spectral data, it is evident that the overall secondary structure of GS_C_-SS is significantly disrupted. This structural distortion, combined with a substantial decrease in hydrophobicity (Figure 4), likely accounts for the complete loss of antimicrobial activity observed for GS_C_-SS.

The biological activity of gramicidin S is associated with its ability to interact with the membranes. For this reason, the process of embedding the peptides in the membranes was closely investigated. Figure 13 shows that three peptides were able to anchor quite well, with differences due to their stapling and spatial extent. The remaining peptide, GS_C_-SS, is an exception—it is ejected from the membrane within the simulation time. This finding is in line with the experimental biological activity data, where this peptide was found to be the least favorable and thus the least promising for further consideration as a potential antimicrobial agent.

**Figure 13:**
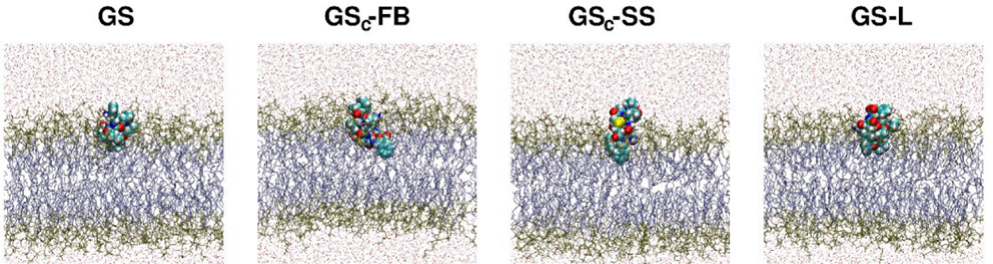
Final structures of the peptides in the membrane after the 0.5 μs of the production MD run showing differences in the embedding efficiency. The online version contains Web Enhanced Objects: videos of the production runs for the peptides: GS, GS_C_-FB, GS_C_-SS, and GS-L.

To quantitatively assess differences in peptide anchoring to the membrane, we conducted a structural analysis based on the distances between the centers of mass of the peptides and the membrane. As shown in Figure 14, the disulfide-stapled GS_C_-SS peptide cannot achieve and maintain a stable position within the membrane. The data align with the experimental biological activity findings presented in Table 1. The other three peptides that exhibited biological activity anchored within the phospholipid bilayer at a similar depth—corresponding to the position of the glycerol backbones—and maintained this position throughout the simulation. Thus, the *in silico* data provide a molecular-scale rationalization for the macroscopic observations. In this case, the chosen theoretical chemistry approaches were able to shed new light on the processes corresponding to the experiment. The connection of various approaches (implicit solvation, classical MD in the aqueous and amphiphilic membrane environments), yielding mutually converged results, allowed us to provide a comprehensive picture of the underlying processes across diverse scales.

**Figure 14:**
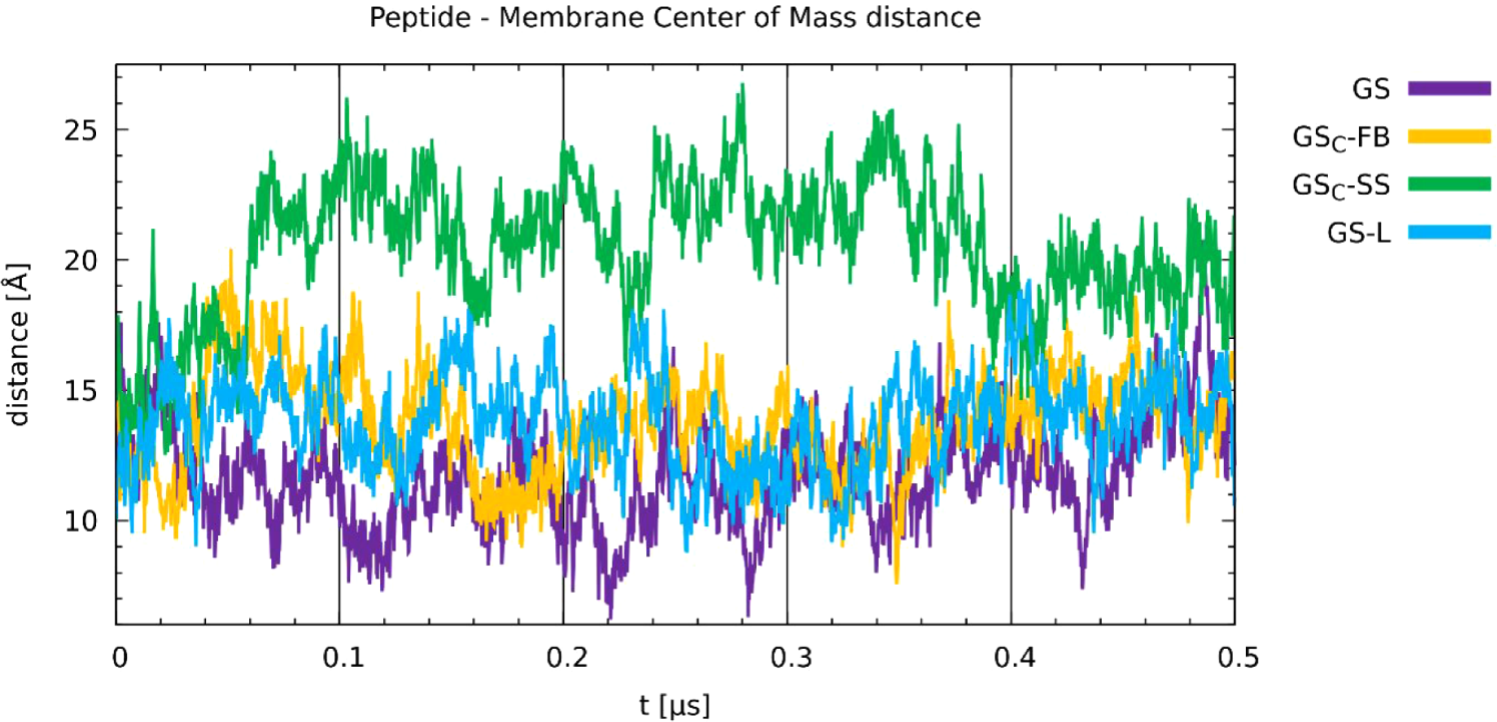
Time evolution of the distance between the barycenters of the peptide and the membrane.

Based on the analyzed structural parameters, embedding efficiency, and depth of penetration, it can be hypothesized that all three bioactive peptides likely operate through a similar mechanism of action. Despite extensive research into the mechanism of action of GS over many years, a clear explanation remains elusive. This highlights the complexity of the task, which persists despite the employment of various analytical methods, including differential scanning calorimetry,^45^ densitometry, sound velocimetry,^46^ isothermal titration calorimetry,^47^ solid-state NMR,^48^ ^31^P NMR,^49^ X-ray diffraction,^50^ Fourier transform infrared spectroscopy,^26^ measurement of ion conductance events,^51^ and molecular dynamics.^52^ The scientific community continues to debate whether GS induces the formation of pores that facilitate the leakage of intracellular substances. However, there is general agreement that GS likely exerts its effects by disrupting the inner membrane.^17^ It is hypothesized that following initial electrostatic interactions with the negatively charged outer surface of the membrane, GS subsequently integrates into the phospholipid bilayer and predominantly localizes at the interface. At this interface, it interacts primarily with the polar head groups and the glycerol backbones of the phospholipids.^26^ Upon insertion of GS, it is plausible that the compound modifies the thermotropic phase behavior by reducing the temperature, enthalpy, and cooperativity associated with the gel/liquid-crystalline phase transition.^47^ The formation of fluid membrane domains may lead to the development of weak points in the lipid bilayer at the interface with gel-phase domains, facilitating cell permeabilization.^53^ It has been observed that GS has a more pronounced effect on anionic phospholipids than on zwitterionic, and the presence of cholesterol slightly decreases its potency.^47^ The slightly increased activity of gramicidin S against Gram-positive bacteria may be attributed to their comparatively higher content of anionic phospholipids when contrasted with Gram-negative bacteria or eukaryotic cells.^54,55^

According to the calculated molecular electrostatic potentials (MEP, Figure S40), positive charges dominate the external surface of the peptides, suggesting that the first step of their mechanism of action—electrostatic interaction with negatively charged components of bacterial membranes—is unlikely to be impeded. The subsequent step, namely, insertion into the phospholipid bilayer, may be significantly hindered by reduced hydrophobicity and conformational flexibility. This is exemplified by the highly hydrophilic and most rigid analog, GS_C_-SS, as membrane insertion depends primarily on hydrophobic interactions and requires squeezing between densely packed phospholipid molecules. However, the correlation between hydrophobicity and membrane insertion does not fully apply to GS_C_-FB and GS-L. Despite their lower hydrophobicity compared to unmodified GS, both analogs exhibited membrane embedding behavior comparable to that of GS. Interestingly, although GS_C_-FB and GS-L possess similar hydrophobicity, their biological activity profiles differ—both in terms of the bacterial strains they affect and their MICs. A key structural distinction between these peptides is their markedly different conformational rigidity. The isolated effect of Leu-to-Cys substitution on activity remains unclear, as the free −SH analog can easily oxidize to form the disulfide analog (GS_C_-SS). We therefore hypothesize that factors beyond hydrophobicity—particularly conformational flexibility—may play a crucial role in determining peptide bioactivity. This hypothesis is consistent with findings reported by Selvarajan et al.^29^ The ability to adopt multiple conformations may facilitate insertion into the tightly packed lipid bilayer. We propose that the loss of GS_C_-FB activity against Gram-negative strains results from its relatively high rigidity among the three bioactive analogs. Molecular dynamics simulations revealed that its structure remained conformationally fixed, even within the membrane environment, which may hinder its ability to penetrate the outer membrane of Gram-negative bacteria. As documented, many cationic and cyclic AMPs or conformationally constrained peptides face significant barriers in penetrating the outer membrane of Gram-negative bacteria due to the dense lipopolysaccharide layer and lack of sufficient translocation mechanisms.^56^ In contrast, GS and GS-L exhibited sufficient flexibility to allow them to squeeze between phospholipids in the outer leaflet. These findings suggest that complete rigidification of the peptide structure may not be an optimal strategy, as it can lead to a total loss of antimicrobial activity. On the other hand, excessive conformational freedom may impair the ability of a peptide to maintain a bioactive structure, thereby requiring higher concentrations to exert its intended antimicrobial effect. This could explain the higher MIC values observed for the linear analog GS-L compared to unmodified GS. Our study demonstrates a clear relationship between structural properties and biological activity. Specifically, we show that a reduction in hydrophobicity, when accompanied by an appropriate modulation of peptide rigidity, can yield β-sheet peptides that retain antimicrobial activity while exhibiting reduced cytotoxicity and hemolytic potential. For instance, GS_C_-FB at its MIC (32 μg/mL) against *E. faecalis* does not induce hemolysis, in contrast to unmodified GS, which causes hemolysis at 16 μg/m—the same as its MIC against this strain. Moreover, at these concentrations, NHDF cell viability is higher for GS_C_-FB than for GS. This effect is even more pronounced in the case of GS-L, which shows significantly improved NHDF cell viability at its MIC (64 μg/mL) against *P. aeruginosa*, compared to unmodified GS.

We showed that excessive rigidification combined with reduced hydrophobicity (e.g., in GS_C_-SS) impairs membrane activity, while moderate rigidity (e.g., in GS_C_-FB) offers a better balance between efficacy and toxicity. These findings underscore the importance of tuning the conformational constraint and hydrophobicity in AMP design. Rather than positioning stapled peptides as superior to gramicidin S, we present them as valuable tools within a broader structure–activity optimization strategy.

## 3 Conclusion

In this work, we explored the influence of conformational rigidity on the biological properties of β-sheet antimicrobial peptides by synthesizing and characterizing three novel gramicidin S analogs: a linear derivative (GS-L), a perfluoroaryl-stapled analog (GS_C_-FB), and a disulfide-stapled analog (GS_C_-SS). By combining rational design, advanced synthetic strategies, biophysical characterization, biological testing, and molecular modeling, we provided a comprehensive evaluation of how structural modifications affect antimicrobial activity, cytotoxicity, and membrane interactions. Biological testing showed that modulation of structural rigidity had a pronounced effect on the peptides’ properties. Both analogs with moderately increased (GS_C_-FB) and decreased (GS-L) rigidity compared to native gramicidin S exhibited reduced cytotoxicity and hemolytic activity. At the same time, these analogs maintained antimicrobial activity, although to a lesser extent than the parent compound. GS_C_-FB retained activity against Gram-positive strains with MIC values 2–4 times higher than GS but was inactive against Gram-negative bacteria. Its reduced flexibility, introduced through stapling, may hinder the peptide’s ability to pass through the outer membrane to reach its site of action—inner leaflet. Decreased hydrophobicity, as indicated by RP-HPLC, may also contribute to this effect but does not fully explain the loss of activity since the equally hydrophobic GS-L retained partial activity against Gram-negative strains with only 2 times higher MIC compared to GS and is less potent against Gram-positive strains compared to GS_C_-FB. The differences in biological activity of the stapled peptides also correlate very well with the secondary structure changes measured with CD. It is believed that a slightly disordered β-sheet in AMPs can reduce cytotoxicity without affecting antimicrobial properties excessively, which was confirmed in our work. GS_C_-FB and GS-L showed smaller deviations from the β-sheet structure than GS_C_-SS, which, with lower hydrophobicity as well as much greater stiffening, did not exhibit any biological activity. Based on MD studies for each of the peptides, we could support experimental challenges based on design and confirm that the hypotheses associated with the idea of the project (biological consequences of tuning β-sheet peptide rigidity) are reproducible using theoretical chemistry tools. MD simulations revealed distinct behaviors of the peptides in membrane environments. GS-L and GS_C_-FB both inserted effectively into the bilayer, although GS-L exhibited dynamic structural adaptations due to its flexibility, while GS_C_-FB adopted a stable, unchangeable conformation. In contrast, the most rigid peptide, GS_C_-SS, despite maintaining its secondary structure, was quickly excluded from the membrane, which aligns with its lack of biological activity. Meanwhile, native GS, recognized as the most active peptide, displayed conformational adaptability in response to its environment while preserving its bioactive structure within the membrane. Our findings revealed that conformational rigidity is a critical but underexplored parameter in the design of β-sheet AMPs. We demonstrated that modulating the rigidity of β-sheet peptides within a reasonable range can lead to a compound with dissociated antimicrobial activity from its harmful side effects. However, obtaining overly stiff compounds, which cannot adapt their structure to the environment, results in the complete disappearance of any biological effects. Our results provide a valuable framework for the future design of β-sheet-based antimicrobial agents with improved safety and efficacy profiles, particularly in the context of multidrug resistance.

## 4 Experimental Section

### 4.1 Synthesis

#### 4.1.1 Reagents

Derivatives of amino acids for peptide synthesis, 2-CTC Resin (1.60 mmol Cl/g), and the coupling reagents TBTU (2-(1*H*-Benzotriazole-1-yl)-1,1,3,3-tetramethylaminium tetrafluoroborate) and PyBop (benzotriazol-1-yloxytripyrrolidinophosphonium hexafluorophosphate) were purchased from NovaBiochem and/or Iris Biotech. The solvents for peptide synthesis (analytical grade) were obtained from Riedel de Haen (DMF) and J. T. Baker (methanol, acetonitrile). LC-MS solvents (water, acetonitrile, and methanol) were purchased from ChemSolve and J. T. Baker. Other reagents used in this work were obtained from Aldrich: triisopropylsilane (TIS), 1-hydroxybenzotriazole (HOBt), *N*,*N*-diisopropylethylamine (DIEA), hexafluorobenzene, and IrisBiotech: trifluoroacetic acid (TFA).

#### 4.1.2 Synthesis of Gramicidin S (GS)

The peptide was synthesized manually on 2-CTC resin (loading 1.60 mmol/g) using 200 mg of resin, following the Fmoc protocol with ultrasonic agitation developed by Wołczański et al.^36^ using DIEA (6 equiv), TBTU (3 equiv), and HOBt (3 equiv) as coupling reagents. The progress of the coupling reaction was monitored by a Kaiser test after each step of the synthesis. Resin loading was performed using the following protocol: the resin was swelled for 30 min in DCM (10 mL/g), then the solution of Fmoc-d-Phe-OH (3 equiv) in DCM with DIEA (6 equiv) was added and mixed for 2 h on a rotary shaker. After that, the capping mixture was added (DCM:MeOH:DIEA; 17:2:1; v/v) and placed in a rotary shaker for 30 minutes. Finally, the resin was washed with DCM (3 × 1 min) and DMF (3 × 1 min). After the synthesis of the whole peptide sequence, the Fmoc protecting group was removed using 25% piperidine in DMF (2 × 3 min) in an ultrasonic bath. The peptidyl resin was then washed with DMF (7 × 1 min), DCM (3 × 1 min), and MeOH (3 × 1 min) and dried. The peptide was cleaved from the resin with DCM:TFA (98:2; v/v) mixture, resulting in ∼80% yield of the crude product (GS-L­(2Boc)). The linear Boc-protected peptide was analyzed by the ESI-MS method and was further subjected to the head-to-tail cyclization using the procedure described by Wadhwani et al.^38^ Briefly, GS-L­(2Boc) was dissolved in degassed DCM (0.5 mg/mL) in a 500 mL round-bottom flask. Solid PyBOP (3 equiv) and a solution of HOBt (3 equiv) in degassed DMF (0.5 mL) were added to the flask, and the mixture was stirred to dissolve. DIEA (6 equiv) was then added to initiate the reaction. The reaction was monitored by ESI-MS and allowed to proceed for 48 h under a nitrogen atmosphere, resulting in an ∼70% yield of the crude product (GS­(2Boc)). The completion of cyclization was confirmed by ESI-MS. After removing the solvent by rotary evaporation, the resulting oil was dissolved in the mixture of TFA:H_2_O (95:5; v/v) for 2 h to remove the Boc-protection group on the δ-amino groups of Orn. After the mixture was evaporated under a nitrogen stream, the obtained product (GS) was dissolved in water and lyophilized. The crude product was dissolved in acetonitrile/water (1:1) and purified directly by preparative HPLC. The identity of the product was confirmed by LC-MS, ESI-MS, and ESI-MS/MS analytical methods, and the purity was determined by HPLC-DAD.

#### 4.1.3 Synthesis of Linear Analog of Gramicidin S (GS-L)

The peptide was synthesized manually on 2-CTC resin (loaded 1.60 mmol Cl/g) using 200 mg of resin according to the Fmoc protocol with ultrasonic agitation developed by Wołczaski et al.^36^ using DIEA (6 equiv), TBTU (3 equiv), and HOBt (3 equiv) as coupling reagents. The progress of the coupling reaction was monitored by the Kaiser test after each step of the synthesis. Resin loading was performed using the following protocol: the resin was swelled for 30 min in DCM (10 mL/g), then the solution of Fmoc-d-Phe-OH (3 equiv) in DCM with DIEA (6 equiv) was added and mixed for 2 h on a rotary shaker. After that, a capping mixture (DCM:MeOH:DIEA; 17:2:1; v/v) was added and placed on a rotary shaker for 30 minutes. Finally, the resin was washed with DCM (3 × 1 min) and DMF (3 × 1 min). After the synthesis of the whole peptide sequence, the Fmoc protecting group was removed in the 25% of piperidine in DMF (2 × 3 min) in an ultrasonic bath. The peptidyl resin was then washed with DMF (7 × 1 min), DCM (3 × 1 min), and MeOH (3 × 1 min) and dried. The peptide was cleaved from the resin with DCM:TFA (98:2; v/v) mixture, resulting in ∼80% yield of the crude product (GS-L­(2Boc)). After the mixture was removed under a nitrogen stream, the resulting product was dissolved in the mixture of TFA:H_2_O (95:5; v/v) for 2 h to remove the Boc-protection group on the δ-amino groups of Orn. After evaporating the mixture, the obtained product (GS-L) was dissolved in water and lyophilized. The crude product was dissolved in acetonitrile/water (1:1) and purified directly by preparative HPLC. The product identity was confirmed by LC-MS, ESI-MS, and ESI-MS/MS analytical methods, and its purity was determined by HPLC-DAD.

#### 4.1.4 Synthesis of Stapled Analog of Gramicidin S (GS_C_-FB)

##### 4.1.4.1 Procedure A

The peptide was synthesized manually on 2-CTC resin (loaded at 1.6 mmol Cl/g) using 200 mg of resin, following the Fmoc protocol with ultrasonic agitation developed by Wołczański et al.^36^ using DIEA (6 equiv), TBTU (3 equiv), and HOBt (3 equiv) as coupling reagents. The progress of the coupling reaction was monitored by the Kaiser test after each step of the synthesis. Resin loading was performed using the following protocol: the resin was swelled for 30 min in DCM (10 mL/g), then the solution of Fmoc-d-Phe-OH (3 equiv) in DCM with DIEA (6 equiv) was added and mixed for 2 h on a rotary shaker. Afterward, capping mixture was added (DCM:MeOH:DIEA; 17:2:1; v/v) and placed in a rotary shaker for 30 minutes. Finally, the resin was washed with DCM (3 × 1 min) and DMF (3 × 1 min). After synthesizing the whole peptide sequence, the Fmoc protecting group was removed in 25% piperidine in DMF (2 × 3 min) in an ultrasonic bath. The peptidyl resin was then washed with DMF (7 × 1 min), DCM (3 × 1 min), and MeOH (3 × 1 min) and dried. The peptide was cleaved from the resin with DCM:TFA (98:2; v/v) mixture, resulting in ∼75% yield of the crude product (GS_C_-L­(2Boc)). GS_C_-L­(2Boc) was analyzed by the ESI-MS method and further subjected to head-to-tail cyclization according to the same procedure as for gramicidin S (see Section 4.2). The completion of the cyclization was tested by ESI-MS on an aliquot of the reaction mixture, but the reaction did not give the desired product.

##### 4.1.4.2 Procedure B Based on NCL

The peptide was synthesized manually on TentaGel R RAM resin (loaded at 0.18 mmol/g) using 400 mg of resin according to the Fmoc protocol with ultrasonic agitation developed by Wołczański et al.^36^ using DIEA (6 equiv) and TBTU (3 equiv) as a coupling reagents. The cyclization of the linear peptide analog was based on the native chemical ligation method proposed by Wierzbicka et al.^37^ The building block *N*-(2-sulfanylethyl)­glycinamide was attached in two steps. First, bromoacetic acid (5 equiv) with DIC (diisopropylcarbodiimide) (5 equiv) in DMF was added three times, each time using a fresh portion of the reagent. The mixture was stirred for 30 min on a rotary mixer, followed by washing with DMF (7 × 1 min) and filtrations. Then, the Trt-cysteamine (6 equiv) with DIEA (12 equiv) in DMF was added, and the mixture was stirred overnight at room temperature on a rotary mixer. Next, the peptidylresin was filtered and washed with DMF (5 × 1 min). Coupling the next amino acid (Orn) to the secondary nitrogen atom required repeating the coupling reaction twice using PyBop (3 equiv) as the coupling reagent. The reaction steps were monitored with both Kaiser and chloranil tests. After the synthesis, the peptidylresin was washed with DMF/DCM, DCM, and MeOH, and dried in a desiccator. The peptide was cleaved from resin with TFA:H_2_O:TIS (95:2.5:2.5) mixture, resulting in ∼95% yield of the crude product. After evaporating trifluoroacetic acid, the linear product was lyophilized and analyzed by ESI-MS. Next, the crude product was subjected to the cyclization reaction. Briefly, the linear peptide (GS_C_-L) was dissolved in 1 mL of citrate acid solution in water (pH 3; 50 mM), and 50 equiv of MESNa (sodium 2-sulfanylethanesulfonate) was added in a volume of citrate acid solution that gave the final peptide concentration of 3 mM. The reaction mixture was incubated (mixed) at 40 °C for 24 h. Then, the pH was adjusted to 7.4 with 8 M NaOH, and 10 equiv of DTT (dithiothreitol) was added. The mixture was incubated for the next 24 h at 40 °C, resulting in >99% yield of the crude product (GS_C_). After this time, the solvent was evaporated, and the sample was desalted by SPE and analyzed by ESI-MS. Briefly, a solid sample of GS_C_ was divided into flasks (7.5 μmoles of peptide in each), and 1.9 mL of 100 μM solution (∼25 equiv) of hexafluorobenzene in degassed DMF and 1.5 mL of 50 mM solution of TRIS base (tris­(hydroxymethyl)­aminomethane) in degassed DMF were added to each. The flasks were vigorously mixed in a shaker for 30 s and left at room temperature in a nitrogen atmosphere for 4.5 h, resulting in ∼70% yield of the crude product (GS_C_-FB). The progress of the reaction was monitored by the ESI-MS method. In the final step, GS_C_-FB was dissolved in methanol and purified by size exclusion chromatography (SEC), followed by purification by preparative HPLC. The identity of the product was confirmed by LC-MS, ESI-MS, and ESI-MS/MS analytical methods, and its purity was determined by HPLC-DAD.

#### 4.1.5 Synthesis of Stapled Analog of Gramicidin S (GS_C_-SS): Cyclo­(−Val–Orn–Cyclo­(Cys-d–Phe–Pro–Val–Orn–Cys)–S–S-d–Phe–Pro−)

The peptide was synthesized manually on TentaGel R RAM Resin (loaded 0.18 mmol/g) using 400 mg of resin, following the Fmoc protocol with ultrasonic agitation developed by Wołczański et al.^36^ using DIEA (6 equiv) and TBTU (3 equiv) as a coupling reagents. The cyclization of the linear peptide analog was based on the native chemical ligation method proposed by Wierzbicka et al.^37^ The building block *N*-(2-sulfanylethyl)­glycinamide was attached in two steps. First, bromoacetic acid (5 equiv) with DIC (5 equiv) in DMF was added three times, each time using a fresh portion of the reagent. The mixture was stirred for 30 min on a rotary mixer, followed by filtration and washing with DMF (7 × 1 min). Then, the Trt-cysteamine (6 equiv) with DIEA (12 equiv) in DMF was added, and the mixture was stirred overnight at room temperature on a rotary mixer. Next, the peptidylresin was filtered and washed with DMF (5 × 1 min). Coupling the next amino acid (Orn) to the secondary nitrogen atom required repeating the coupling reaction twice using PyBop (3 equiv) as the coupling reagent. The reaction steps were monitored with both Kaiser and chloranil tests. After the synthesis, the peptidylresin was washed with DMF/DCM, DCM, and

MeOH and dried in a desiccator. The peptide was cleaved from resin with TFA:H_2_O:TIS (95:2.5:2.5) mixture, resulting in ∼95% yield of the crude product (GS_C_-L). After evaporating trifluoracetic acid, the product was lyophilized and analyzed by ESI-MS. Next, the crude product was subjected to a cyclization reaction. Briefly, GS_C_-L was dissolved in 1 mL of citrate acid solution in water (pH 3; 50 mM), and 50 equiv of MESNa was added in a volume of citrate acid solution that gave the final peptide concentration of 3 mM. The reaction mixture was incubated (mixed) at 40 °C for 24 h. Then, the pH was adjusted to 7.4 with 8 M NaOH, and 10 equiv of DTT was added. The mixture was incubated for the next 24 h at 40 °C, resulting in >99% yield of the crude product (GS_C_). After this time, the solvent was evaporated, and the sample was desalted by SPE and analyzed by analytical methods: ESI-MS/MS and HPLC-DAD. Briefly, a solid sample of GS_C_ was dissolved in 50 mM solution of TRIS base in DMF and left at room temperature for 4.5 h, resulting in >99% yield of the crude product (GS_C_-SS). The progress of the reaction was monitored by the ESI-MS method. In the last step, the stapled analog was dissolved in acetonitrile/water (1:1) and purified directly by preparative HPLC. The product identity was confirmed by LC-MS, ESI-MS, and ESI-MS/MS analytical methods, and purity was determined by HPLC-DAD.

#### 4.1.6 Purification

All compounds were obtained with a purity >95%, as determined by HPLC-DAD analysis.

##### 4.1.6.1 Purification of GS, GS-L and GS_C_-SS

The products were purified by preparative reversed-phase HPLC on a Vydac C18 column (22 × 250 mm), using solvent systems S1:0.1% aqueous TFA, S2:80% acetonitrile +0.1% TFA. A linear gradient was individually set for each compound with a flow rate of 7.0 mL/min and UV detection at 210 nm. The fractions were collected and lyophilized, and their identities were confirmed by HPLC-DAD and LC-MS.

##### 4.1.6.2 Purification of GS_C_-FB

In the first step, the product was preliminarily purified by SEC on a glass column (20 mm × 600 mm), using Sephadex LH-20 as a gel filtration medium and MeOH as an eluent, with a flow rate of 25 mL/h. Fractions were collected, analyzed with ESI-MS, and lyophilized. The fractions containing the desired product were combined and purified by a preparative reversed-phase HPLC, following the same procedure as for GS, GS-L, and GS_C_-SS. Its identity was confirmed by HPLC-DAD and LC-MS.

### 4.2 Physicochemical Data

#### 4.2.1 LC-MS Analysis

The LC-MS analysis was performed on Shimadzu LC IT-TOF. Separation was carried out on an RP-Zorbax (50 × 2.1 mm, 3.5 μm) column with a gradient elution of 0–80% B in A for all compounds (A= 0.1% HCOOH in water; B = 0.1% HCOOH in MeCN) at room temperature over a period of 15 min (flow rate: 0.2 mL/min).

#### 4.2.2 ESI-MS and ESI-MS/MS Analysis

ESI-MS experiments were performed using a Bruker Compact qTOF instrument (Bruker Daltonic, Germany) equipped with an ESI source. The instrument was operated in positive-ion mode and calibrated with the ESI-L Low Concentration Tuning Mix (Agilent Technologies). The mass accuracy was better than 5 ppm. An acetonitrile/water/formic acid (50:50:0.1) mixture was used as the solvent for recording the mass spectra. The sample was infused at a flow rate of 3 μL/min. The instrumental parameters were as follows: scan range 100–2000 *m*/*z*; drying gas: nitrogen; the temperature of drying gas: 200 °C; the potential between the spray needle and the orifice: 3.5 kV. The obtained mass spectra were analyzed using the Bruker Data Analysis (Bruker Daltonic, Germany) software. In the MS/MS mode, the quadrupole was used to select the precursor ions, which were fragmented in the hexapole collision cell by applying argon as the collision gas. The obtained fragments were subsequently analyzed with a TOF mass analyzer. For the MS/MS measurements, the voltage over the hexapole collision cell varied from 15 to 40 V.

#### 4.2.3 HPLC-DAD Analysis

The HPLC-DAD analysis was performed on a UHPLC Nexera equipped with a PDA detector. Separation was carried out on an RP-Zorbax (50 × 2.1 mm, 3.5 μm) column with a gradient elution of 0–80% B in A for all compounds (A= 0.1% HCOOH in water; B = 0.1% HCOOH in MeCN) at room temperature over a period of 15 min (flow rate: 0.1 mL/min).

#### 4.2.4 Circular Dichroism (CD) Spectroscopy

The measurements were carried out using a J-600 Circular Dichroism Spectrophotometer equipped with a temperature control accessory of the cell holder under a constant nitrogen flow. Peptides’ secondary structure was measured with far-UV (195–280 nm) and 1 mm of cuvette path length. Peptide concentrations were 50 μM. For each CD spectrum, an average of 20 scans of the same sample was collected at 25 °C with a step resolution of 0.2 nm, a scan speed of 50 nm per minute, and a bandwidth of 1 nm. The data were processed by Spectra Manager Analysis software provided by JASCO as follows: the spectrum of each sample was corrected to the baseline, smoothed with a Savitzky-Golay filter, and converted to molar ellipticity.

### 4.3 Biological Activity

#### 4.3.1 *In Vitro* Antibacterial Activity

The antibacterial properties of four peptides (GS, GS-L, GS_C_-FB, GS_C_-SS) were assayed using the following Gram-positive bacteria: *Staphylococcus aureus* ATCC 25923, *Enterococcus faecalis* ATCC 29212, and Gram-negative bacteria: *Pseudomonas aeruginosa* ATCC 15422 and *Escherichia coli* ATCC 25922. The peptides’ MICs were determined using the protocol recommended by the National Committee for Clinical Laboratory Standards (NCCLS).^57^ The peptides were dissolved in dimethyl sulfoxide (DMSO) to obtain a concentration of 12.8 mg/mL. The final concentrations of each peptide were ranged from 2 to 128 μg/mL. Growth control wells contain 1% DMSO. Each bacterial suspension was standardized at a cell density of 1–2 × 10^8^ colony-forming units ml^–1^ (CFU ml^–1^) by using a 0.5 McFarland standard, and each well was inoculated with 10 μL of the bacterial suspension. Strains were cultured with peptides in Mueller–Hinton broth for 24 h at 37 °C in a 96-well plate. After incubation, 20 μL of 2,3,5-triphenyltetrazolium chloride (TTC, Merck Millipore, Darmstadt, Germany) solution 0.125% (w/v) was added to each well, and the plates were incubated again for 2 h. After the incubation, visual readings were performed in a microplate reader (Spark, Tecan Trading AG., Switzerland) at 540 nm. The MIC was defined as the lowest concentration of the tested compounds at which no bacterial growth occurred. All measurements were performed in three independent experiments.

#### 4.3.2 Hemolytic Assay

Sheep red blood cells (SRBC; Pro Animali Company, Wroclaw, Poland) were centrifuged at 2500 rpm for 5 min at 10 °C. Next, the erythrocytes obtained were washed three times with PBS (10 mM phosphate and 150 mM NaCl, pH 7.4). The peptides were diluted in PBS to prepare 1.0 mL volumes of 2, 4, 8, 16, 32, 64, and 128 μg/mL solutions, and then SRBCs ∼2 × 10^7^ were added to 1.0 mL solutions. After 30 min of incubation at room temperature, the cells were centrifuged, and the supernatant was used to measure the absorbance of the liberated hemoglobin at 540 nm using a microplate reader (Spark, Tecan Trading AG., Switzerland). Two controls were prepared without compounds: the negative control received sterile PBS, while the positive control received 0.1% Triton X-100. The minimum concentration of peptides required to induce 50% hemolysis (HC_50_) was determined, with PBS as the negative control (0% hemolysis). The average value will be calculated from triplicate assays.

#### 4.3.3 Cytotoxicity Assay

The Normal Human Dermal Fibroblasts (NHDF) (Lonza, Basel, Switzerland) were cultured in α-Minimum Essential Medium (α-MEM, Institute of Immunology and Experimental Therapy (IITD), Wroclaw, Poland) containing 10% Fetal Bovine Serum (FBS; Capricorn Scientific GmbH, Ebsdorfergrund, Germany), 2 mM glutamine, and antibiotics (100 U/mL penicillin, 100 μg/mL streptomycin; Sigma, St. Louis, MO, USA). The NHDF cells were seeded onto 96-well plates at a density of 3 × 10^3^ cells per well in 100-μL medium and incubated at 37 °C in 5% CO_2_ for 24 h or when they reached 80% confluence. Four peptides (GS, GS-L, GS_C_-FB, GS_C_-SS) were dissolved in DMSO to obtain a concentration of 12.8 mg/mL. NHDF cells were treated for 24 h with increasing concentrations of peptides (2–128 μg/mL) added to the cell culture medium. The control wells were maintained with 1% DMSO. The cell proliferation rate was determined with the standard MTT (3-(4,5-dimethylthiazol-2-yl)-2,5-diphenyltetrazolium bromide) (Sigma, St. Louis, MO, USA) assay procedure.^58^ All measurements were performed in three independent experiments. The percentage of cell viability was calculated as follows: percentage of cell viability = [(A treatment – A blank)/(A control – A blank)] × 100 (where A = absorbance at λ = 570 nm). Statistical significance was determined using Student’s *t*-test. The significance level was set at *p* < 0.05.

### 4.4 Computational Methodology

#### 4.4.1 Static Models

The model of gramicidin S was prepared based on the crystal structure of gramicidin S^59^ available in the Cambridge Structural Database (CSD)^60^ under the deposition number 1870209. However, the models of stapled analogs (GS_C_-FB and GS_C_-SS) of gramicidin S were built manually by modifying the available crystal structure. The Avogadro program was used for this purpose.^61^ It is worth mentioning that the ornithine residues were protonated. Next, the energy minimization was performed based on DFT.^33,34^ The functional M06–2X^62^ and the def2-TZVP basis set^63^ were applied. Subsequently, harmonic frequencies were computed to confirm that the structures obtained correspond to the minima on the potential energy surfaces (PES). The simulations were carried out *in vacuo* and with a solvent reaction field—the IEF-PCM model was used, with water as the solvent.^64^ At this point, it is necessary to underline that the simplest gas-phase models were used only as a starting point for further IEF-PCM simulations. The wave functions for electronic structure analyses were obtained using the same level of theory. This part of the simulations was performed using the Gaussian 16 C.01 suite of programs.^65^ The electronic structure analysis includes results derived from the quantum theory of atoms in molecules (QTAIM).^66^ Additionally, the MEP was computed to show parts of the peptides with positive and negative potential regions. The electronic structure analysis was carried out with the assistance of the AimAll^67^ and VMD 1.9.3 ^68^ programs.

#### 4.4.2 Molecular Dynamics Simulations

Classical molecular dynamics (MD) simulations were performed using the Amber22 suite of programs.^69^ Two independent MD models were prepared: the first one was devoted to the simulations of the investigated peptides in aqueous solution, while the second one—the interactions of the investigated peptides with a membrane model. The following MD protocol was used with small variations in both MD models. The models of gramicidin S and its stapled analogs were prepared based on the structures obtained based on DFT/M06–2X/def2-TZVP/IEF-PCM. Next, each of the studied peptides was placed in a rectangular box with approximate (49 × 49 × 49 Å) initial dimensions filled with water (approximately 2850–3000 water molecules, depending on the model). Periodic boundary conditions (PBCs) were applied during the simulations to simulate the bulk solution. The chloride anions were used to neutralize the cationic peptides. The Amber ff19SB force field^70^ was applied for the peptides, while the explicit solvation model was represented by TIP3P.^71^ Nonbonded van der Waals and short-range electrostatic interactions were switched off at 10 Å, while the particle mesh Ewald approach was applied to evaluate the long-range electrostatic interactions.^72–74^ All studied models of peptides were prepared with the assistance of the LEAP program implemented in the Amber22 suite of programs. The MD simulations consisted of three steps. In the first step, the energy minimization of the systems (1000 steps, steepest descent algorithm) was performed to prepare the models for further studies. In this part of the simulations, the focus was on removing short contacts between the peptides and water molecules. In the second step, the equilibration of the models was carried out using the NPT ensemble (0.4 ns). During this part of the MD, the investigated models were heated to 300 K and thermostated using Langevin thermostats.^75^ The barostat set at 1 atm was used for pressure control.^76^ A time step of 2 fs was employed. The density of the system stabilized after 0.1 ns. The third step was the so-called production run. The simulations continued with the NPT ensemble, using Langevin thermostats and barostats set at 300 K and 1 atm conditions; data were collected for 1 μs for each of the studied peptides. The SHAKE algorithm was applied to maintain fixed bond lengths involving hydrogen atoms.^77^ Based on the obtained trajectories, postprocessing was performed, which consisted of:

- analyses of the whole structure (room mean square deviation [RMSD], radius of gyration [RGYR], solvent-accessible surface area [SASA], and polar surface area [PSA]);
- analyses of the selected fragments of the peptides (analyses of the α-carbons of Orn, Leu, Cys, and Val of GS, GS-L, GS_C_-FB, and GS_C_-SS; root mean square fluctuation [RMSF]);
- analyses of the hydrogen bond network established between the peptide and water molecules located in the interior of the peptides.

The last part of the theoretical study contains the models of the peptides embedded in the model of the membrane. The model of the DMPC membrane was generated using the PACKMOL-MEMGEN facility of the Amber22 package^69^ ensuring solvation of the membrane. The lipids were described with the classical LIPID21 force field parameters.^78^ The investigated peptides were manually placed close to the center of the membrane, and the overlapping residues were removed. The resulting simulation cells were of approximate 84 × 84 × 85 Å initial dimensions. The initial minimization, equilibration, and production run were carried out with the same protocol as provided above. The production data were collected for 0.5 μs for each of the studied peptides. The data analysis for the membrane part of the study contains:

- analyses of the whole structure [room mean square deviation [RMSD], radius of gyration [RGYR], and solvent-accessible surface area [SASA]);
- analyses of the selected fragments of the peptides (analyses of the α-carbons of Orn, Leu, Cys, and Val of GS, GS-L, GS_C_-FB, and GS_C_-SS);
- analysis of the time evolution of the peptide penetration of the membrane (the distance between the centers of mass of the peptide and the membrane).

The graphical presentation and data analyses were prepared with the assistance of the VMD 1.9.3,^68^ Molden,^79^ Gnuplot,^80^ and ChimeraX^81^ programs. The MD runs were carried out in triplicate, and no significant variations were registered between the runs—see Figure S41.

## Supplementary Material













## Notes

Table: S1: List of obtained peptides; S2–S5: Analytical data for obtained peptides; S6: List of dihedral angle values measured for obtained peptides; S7: Selected hydrogen bonds present in GS obtained at different levels of theory; S8: Selected hydrogen bonds present in obtained peptides. Figures S1–S26: LC-MS and ESI-MS data for obtained peptides and their intermediates; S27–S30: HPLC-DAD chromatograms of obtained peptides; S31–S34: DFT optimized structures of obtained peptides; S35–S39: DFT optimized structures of obtained peptides indicating noncovalent interactions based on the QTAIM; S40: MEP surfaces of obtained peptides; S41: RMSD calculated for obtained peptides in water; S42: RMSF calculated for obtained peptides in water; S43: SASA calculated for obtained peptides in water; S44: PSA calculated for obtained peptides in water; S45–S46: Frames from the MD simulations in water showing the expanded GS structure and GS_C_-FB structure (PDF)
Molecular formula strings and molecular dynamics data containing: 1. AMBER input files for minimization (min.mdin), equilibration (eq 1.mdin or eq 2.mdin) and production run (md.mdin) 2. AMBER topology (.prmtop) and initial coordinates (.inpcrd) 3. AMBER NETCDF restart data after equilibration (.rst) 4. PDB files for visualization (ZIP)
Video of the production runs for the peptide GS (AVI)
Video of the production runs for the peptide GSC-FB (AVI)
Video of the production runs for the peptide GSC-SS (AVI)
Video of the production runs for the peptide GS-L (AVI)

## Abbreviations

2-CTC: 2-chlorotrityl chloride
AA: amino acid
AMPs: antimicrobial peptides
AMR: antimicrobial resistance
ATCC: American type culture collection
Boc: *tert*-butyloxycarbonyl group
CD: circular dichroism spectroscopy
CSD: Cambridge Structural Database
DIC: diisopropylcarbodiimide
DIEA: *N*,*N*-diisopropylethylamine
DFT: density functional theory
DMF: *N*,*N*-dimethylformamide
DTT: dithiothreitol
ESI-MS: electrospray ionization mass spectrometry
ESI-MS/MS: electrospray ionization tandem mass spectrometry
FBS: fetal bovine serum
Fmoc: fluorenylmethyloxycarbonyl group
HC_50_: 50% hemolytic concentration
HDPs: host defense peptides
HOBt: 1-hydroxybenzotriazole
HPLC-DAD: high-performance liquid chromatography with diode-array detection
IEF-PCM: integral equation formalism Polarizable Continuum Model
IT-TOF: ion trap time-of-flight
LC-MS: liquid chromatography–mass spectrometry
MD: molecular dynamics
MeCN: acetonitrile
MeOH: methanol
MEP: molecular electrostatic potentials
MESNa: sodium 2-sulfanylethanesulfonate
MIC: minimum inhibitory concentration
MTT: 3-(4,5-dimethylthiazol-2-yl)-2,5-diphenyltetrazolium bromide
NHDF: normal human dermal fibroblasts
NCL: native chemical ligation
NMR: nuclear magnetic resonance
Orn: ornithine
PBC: periodic boundary conditions
PES: potential energy surface
PyBop: benzotriazol-1-yloxytripyrrolidinophosphonium hexafluorophosphate
QTAIM: quantum theory of atoms in molecules
qTOF: quadrupole time-of-flight
RDF: Radial Distribution Function
RGYR: radius of gyration
RMSD: root mean square deviation
RMSF: root mean square fluctuation
RP-HPLC: reversed-phase high-performance liquid chromatography
RT: retention time
SASA: solvent-accessible surface area
SEC: size exclusion chromatography
S_N_Ar: nucleophilic aromatic substitution
SRBC: sheep red blood cells
TBTU: 2-(1*H*-benzotriazole-1-yl)-1,1,3,3-tetramethylaminium tetrafluoroborate
TFA: trifluoroacetic acid
TIC: total ion chromatogram
TIS: triisopropylsilane
TRIS: tris­(hydroxymethyl)­aminomethane
Trt: trityl group
VMD: visual molecular dynamics
XIC: extracted ion chromatogram
