# Supplementary material for: Rigidity vs Activity: Design of Gramicidin S Analogs against Multidrug-Resistant Bacteria Based on Molecular Engineering
Source: J Med Chem. 2025 Sep 30;68(19):20243–63. doi: 10.1021/acs.jmedchem.5c01234 (PMC12516685; doi:10.1021/acs.jmedchem.5c01234)
Supplement: Supplementary file 1 [file jm5c01234_si_001.pdf]

Supporting Information

Rigidity vs activity: design of gramicidin S analogs against multidrug-resistant bacteria based on molecular engineering

Mikołaj Śleziak[1], Jarosław J. Panek[1], Tomasz Janek[2], Aneta Jezierska[1], Monika Kijewska[1*]*

[1] University of Wrocław, Faculty of Chemistry, F. Joliot-Curie St. 14, 50-383 Wrocław, Poland

[2] Wrocław University of Environmental and Life Sciences, Faculty of Biotechnology and Food Science, Chełmońskiego St. 37, 51-630 Wrocław, Poland

ORCID: 0009-0004-4595-2010 (M.S.)
ORCID: 0000-0002-2952-9739 (J.J.P.)
ORCID: 0000-0003-1939-5500 (T.J.)
ORCID: 0000-0001-6601-9124 (A.J.)
ORCID: 0000-0001-6227-7169 (M.K.)

Correspondence should be addressed to:

* aneta.jezierska@uwr.edu.pl (A.J.), monika.kijewska@uwr.edu.pl (M.K.)

Table of contents:

Table S1. List of the obtained peptides. S6

Table S2. Analytical data for **GS_C-FB**. S9

Table S3. Analytical data for **GS_C-SS**. S10

Table S4. Analytical data for **GS**. S11

Table S5. Analytical data for **GS-L**. S12

Figure S1. ESI-MS spectrum of crude **GS_C-L** (positive ion mode). S13

Figure S2. ESI-MS of crude **GS_C-L** in zoom range at m/z 1254-1262 (top) and simulated for pseudomolecular ion $[M+H]^+$ where M= $C_{58}H_{90}N_{14}O_{11}S_3$ (bottom). S13

Figure S3. ESI-MS spectrum of crude **GS_C** (after cyclization via native chemical ligation); positive ion mode. S14

Figure S4. ESI-MS of crude **GS_C** (after cyclization via native chemical ligation) in zoom range at m/z 1120-1128 (top) and simulated for pseudomolecular ion $[M+H]^+$ where M = $C_{54}H_{80}N_{12}O_{10}S_2$ (bottom). S14

Figure S5. LC-MS (**TIC**) of **GS_C-FB**. Conditions: RP-Zorbax column (50 × 2.1 mm, 3.5 µm); gradient elution of 0-80% B in A (A= 0.1% HCOOH in water; B= 0.1% HCOOH in MeCN) in 15 min; flow rate: 0.2 ml/min. S15

Figure S6. LC-MS (**XIC** for m/z 634.2693) of **GS_C-FB**. Conditions: RP-Zorbax column (50 × 2.1 mm, 3.5 µm); gradient elution of 0-80% B in A (A= 0.1% HCOOH in water; B= 0.1% HCOOH in MeCN) in 15 min; flow rate: 0.2 ml/min. S15

Figure S7. ESI-MS spectrum of **GS_C-FB** (positive ion mode). S16

Figure S8. ESI-MS of **GS_C-FB** in zoom range at m/z 1265-1274 (top) and simulated for pseudomolecular ion $[M+H]^+$ where M = $C_{60}H_{78}F_4N_{12}O_{10}S_2$ (bottom). S16

Figure S9. ESI-MS/MS spectrum of **GS_C-FB** for precursor ion m/z 634.2693 (positive ion mode; collision energy: 20 eV). S17

Figure S10. LC-MS (**TIC**) of **GS_C-SS**. Conditions: RP-Zorbax column (50 × 2.1 mm, 3.5 µm); gradient elution of 0-80% B in A (A= 0.1% HCOOH in water; B= 0.1% HCOOH in MeCN) in 15 min; flow rate: 0.2 ml/min. S17

Figure S11. LC-MS (**XIC** for *m/z* 560.2799) of **GS_C-SS**. Conditions: RP-Zorbax column (50 × 2.1 mm, 3.5 µm); gradient elution of 0-80% B in A (A= 0.1% HCOOH in water; B= 0.1% HCOOH in MeCN) in 15 min; flow rate: 0.2 ....S18

Figure S12. ESI-MS spectrum of **GS_C-SS** (positive ion mode). S18

Figure S13. ESI-MS of **GS_C-SS** in zoom range at m/z 1118-1125 (top) and simulated for pseudomolecular ion $[M+H]^+$ where M = $C_{54}H_{78}N_{12}O_{10}S_2$ (bottom). S19

Figure S14. ESI-MS/MS spectrum of **GS_C-SS** for precursor ion m/z 560.2799 (positive ion mode; collision energy: 20 eV). S19

Figure S15. ESI-MS spectrum of crude **GS-L(2Boc)** (positive ion mode). S20

Figure S16. ESI-MS of crude **GS-L(2Boc)** in zoom range at m/z 1358-1366 (top) and simulated for pseudomolecular ion $[M+H]^+$ where M = $C_{70}H_{110}N_{12}O_{15}$ (bottom). S20

Figure S17. LC-MS (TIC) of **GS**. Conditions: RP-Zorbax column (50 × 2.1 mm, 3.5 µm); gradient elution of 0-80% B in A (A= 0.1% HCOOH in water; B= 0.1% HCOOH in MeCN) in 15 min; flow rate: 0.2 ml/min. S21

Figure S18. LC-MS (XIC for m/z 571.3675) of **GS**. Conditions: RP-Zorbax column (50 × 2.1 mm, 3.5 µm); gradient elution of 0-80% B in A (A= 0.1% HCOOH in water; B= 0.1% HCOOH in MeCN) in 15 min; flow rate: 0.2 ml/min. S21

Figure S19. ESI-MS spectrum of **GS** (positive ion mode). S22

Figure S20. ESI-MS of **GS** in zoom range at m/z 1140-1148 (top) and simulated for pseudomolecular ion $[M+H]^+$ where M = $C_{60}H_{92}N_{12}O_{10}$ (bottom). S22

Figure S21. ESI-MS/MS spectrum of **GS** for precursor ion m/z 571.3675 (positive ion mode; collision energy: 25 eV). S23

Figure S22. LC-MS (TIC) of **GS-L**. Conditions: RP-Zorbax column (50 × 2.1 mm, 3.5 µm); gradient elution of 0-80% B in A (A= 0.1% HCOOH in water; B= 0.1% HCOOH in MeCN) in 15 min; flow rate: 0.2 ml/min. S23

Figure S23. LC-MS (XIC for m/z 580.3673) of **GS-L**. Conditions: RP-Zorbax column (50 × 2.1 mm, 3.5 µm); gradient elution of 0-80% B in A (A= 0.1% HCOOH in water; B= 0.1% HCOOH in MeCN) in 15 min; flow rate: 0.2 ml/min. S24

Figure S24. ESI-MS spectrum of **GS-L** (positive ion mode). S24

Figure S25. ESI-MS of **GS-L** in zoom range at m/z 579-583 (top) and simulated for pseudomolecular ion $[M+2H]^{2+}$ where M = $C_{60}H_{94}N_{12}O_{11}$ (bottom). S25

Figure S26. ESI-MS/MS spectrum of **GS-L** for precursor ion m/z 580.3673 (positive ion mode; collision energy: 25 eV). S25

Figure S27. HPLC-DAD chromatogram of **GS**. Conditions: RP-Zorbax column (50 × 2.1 mm, 3.5 µm); gradient elution of 0-80% B in A (A = 0.1% HCOOH in water; B = 0.1% HCOOH in MeCN) in 15 min; flow rate: 0.2 ml/min; detection – DAD, 210 nm. S26

Figure S28. HPLC-DAD chromatogram of GS_C-FB. Conditions: RP-Zorbax column (50 × 2.1 mm, 3.5 µm); gradient elution of 0-80% B in A (A = 0.1% HCOOH in water; B = 0.1% HCOOH in MeCN) in 15 min; flow rate: 0.2 ml/min; detection – DAD, 210 nm. S26

Figure S29. HPLC-DAD chromatogram of **GS_C-SS**. Conditions: RP-Zorbax column (50 × 2.1 mm, 3.5 µm); gradient elution of 0-80% B in A (A = 0.1% HCOOH in water; B = 0.1% HCOOH in MeCN) in 15 min; flow rate: 0.2 ml/min; detection – DAD, 210 nm. S27

Figure S30. HPLC-DAD chromatogram of **GS-L**. Conditions: RP-Zorbax column (50 × 2.1 mm, 3.5 µm); gradient elution of 0-80% B in A (A = 0.1% HCOOH in water; B = 0.1% HCOOH in MeCN) in 15 min; flow rate: 0.2 ml/min; detection – DAD, 210 nm. S27

Figure S31. GS structure optimized at the M06-2X/def2-TZVP level of theory with solvent reaction field (IEF-PCM, water as a solvent). Side view (left) and top view (right). Hydrogen atoms not involved in the hydrogen bond formation are omitted for clarity. Dotted cyan lines indicate the presence of intramolecular hydrogen bonds. S28

Figure S32. GS_C-FB structure optimized at the M06-2X/def2-TZVP level of theory with solvent reaction field (IEF-PCM, water as a solvent). Side view (left) and top view (right). Hydrogen atoms not involved in the hydrogen bond formation are omitted for clarity. Dotted cyan lines indicate the presence of intramolecular hydrogen bonds. S29

Figure S33. GS_C-SS structure optimized at the M06-2X/def2-TZVP level of theory with solvent reaction field (IEF-PCM, water as a solvent). Side view (left) and top view (right). Hydrogen atoms not involved in the hydrogen bond formation are omitted for clarity. Dotted cyan lines indicate the presence of intramolecular hydrogen bonds. S29

Figure S34. GS-L structure optimized on M06-2X/def2-TZVP level of theory with solvent reaction field (IEF-PCM, water as a solvent). Side view (left) and top view (right). Hydrogen atoms not involved in the hydrogen bond formation are omitted for clarity. Dotted cyan lines indicate the presence of intramolecular hydrogen bonds....................S29

Table S6. Dihedral angle values obtained at the M06-2X/def2-TZVP level of theory with solvent reaction field (IEF-PCM, water as a solvent) for GS, GS_C-FB, GS_C-SS, and GS-L structures....................S30

Table S7. Selected hydrogen bonds present in GS obtained at different levels of theory in the gas phase and compared with the literature crystal structure. The calculations were performed using various functionals and def2-TZVP basis set. S31

Table S8. Selected hydrogen bonds present in GS and its analogs. The data was obtained as a result of the DFT/M06-2X/def2-TZVP with IEF-PCM and water as a solvent. The hydrogen bond energy was estimated using data from the QTAIM and based on EML equation. Donor-acceptor distances in parentheses for GS are experimental values [Ref. 1]. S32

Figure S35. The top view of the GS structure calculated at the M06-2X/def2-TZVP level of theory, with the solvent reaction field (IEF-PCM, water as a solvent) indicating non-covalent interactions based on the QTAIM. The yellow ellipsoids indicate intramolecular hydrogen bonds presented in Table 3. The dotted lines indicate intramolecular interactions for which the QTAIM method detected bond critical points (BCPs)....................S34

Figure S36. Top view of GS model calculated at the M06-2X/def2-TZVP level of theory with solvent reaction field (IEF-PCM, water as a solvent) indicating non-covalent interactions based on the QTAIM. The dotted lines indicate intramolecular interactions for which the QTAIM method detected bond critical points (BCPs). S34

Figure S37. Top view of GS_C-FB model calculated at the M06-2X/def2-TZVP level of theory with solvent reaction field (IEF-PCM, water as a solvent) indicating non-covalent interactions based on the QTAIM. The dotted lines indicate intramolecular interactions for which the QTAIM method detected bond critical points (BCPs). S35

Figure S38. Top view of GS_C-SS model calculated at the M06-2X/def2-TZVP level of theory with solvent reaction field (IEF-PCM, water as a solvent) indicating non-covalent interactions based on the QTAIM. The dotted lines indicate intramolecular interactions for which the QTAIM method detected bond critical points (BCPs). S35

Figure S39. Top view of GS-L model calculated at the M06-2X/def2-TZVP level of theory with solvent reaction field (IEF-PCM, water as a solvent) indicating non-covalent interactions based on the QTAIM. The dotted lines indicate intramolecular interactions for which the QTAIM method detected bond critical points (BCPs). S36

Figure S40. Molecular Electrostatic Potential (MEP) surfaces of GS, GS_C-FB, GS_C-SS, and GS-L. Blue color indicates the positive MEP while the red – negative one....................S37

Figure S41. Root Mean Square Deviation (RMSD) calculated for GS, GS-L, GS_C-FB, and GS_C-FB (three MD runs for the set of the studied peptides) in water. S38

Figure S42. Root Mean Square Fluctuation (RMSF) calculated for α-carbon atoms of GS, GS-L, GS_C-FB, and GS_C-FB in water. S39

Figure S43. Solvent-Accessible Surface Area (SASA) calculated for GS, GS-L, GS_C-FB, and GS_C-FB in water. S40

Figure S44. Time-evolution of the polar surface area (PSA) obtained for GS, GS-L, GS_C-FB, and GS_C-SS in water. S40

Figure S45. Frame from the MD simulations showing the expanded GS structure. The dotted cyan lines indicate the presence of hydrogen bonds formed between GS and water molecules (rendered as ball-and-stick model). Color coding: grey – carbon, blue – nitrogen, red – oxygen, and white – hydrogen. S41

Figure S46. Frame from the MD simulations showing the GS_C-FB structure. The dotted cyan lines indicate the presence of hydrogen bonds formed between GS_C-FB and water molecule (rendered as ball-and-stick model). Color coding: grey – carbon, blue – nitrogen, red – oxygen, yellow – sulfur, green – fluorine, and white – hydrogen. S41

Table S1. List of the obtained peptides.

GS	Cyclo(-Val-Orn-Leu-D-Phe-Pro-)$_2$
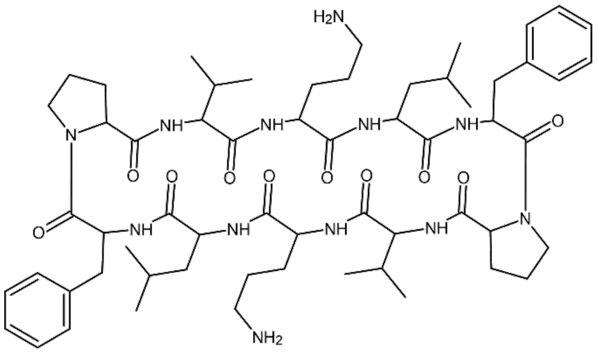	
GS_C-FB	Cyclo(-Val-Orn-cyclo(Cys-D-Phe-Pro-Val-Orn-Cys)4FB-D-Phe-Pro-)
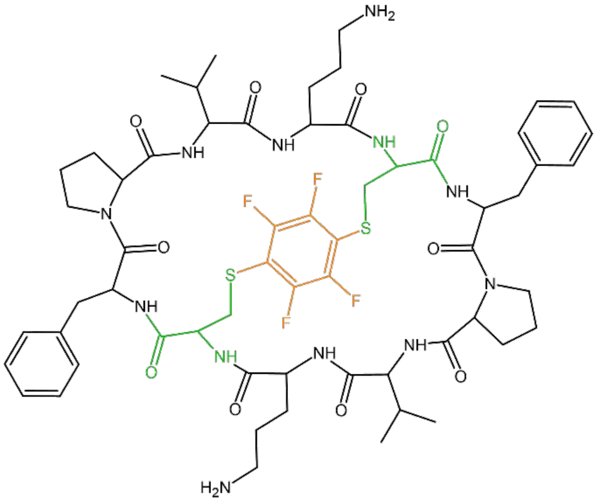	
GS_C-SS	Cyclo(-Val-Orn-cyclo(Cys-D-Phe-Pro-Val-Orn-Cys)-S-S-D-Phe-Pro-)
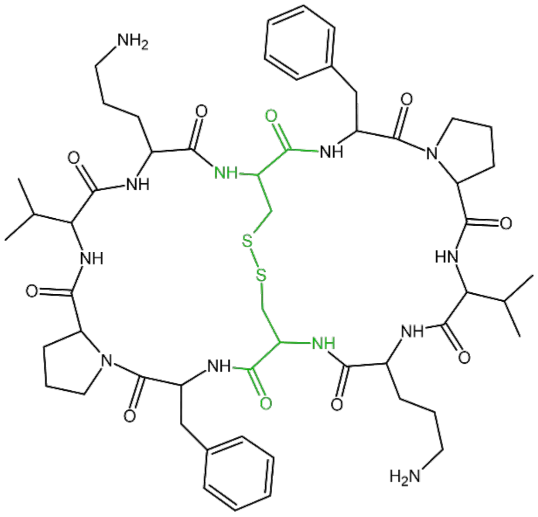	

Table S1 (Continuation). List of the obtained peptides.

GS-L	**H-Pro-Val-Orn-Leu-D-Phe-Pro-Val-Orn-Leu-D-Phe-OH**
	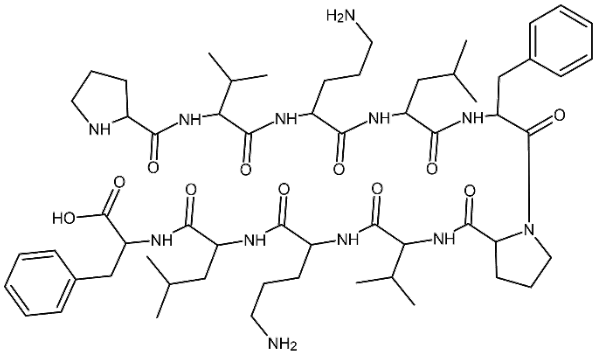
**GS-L(2Boc)**	**H-Pro-Val-Orn(Boc)-Leu-D-Phe-Pro-Val-Orn(Boc)-Leu-D-Phe-OH**
	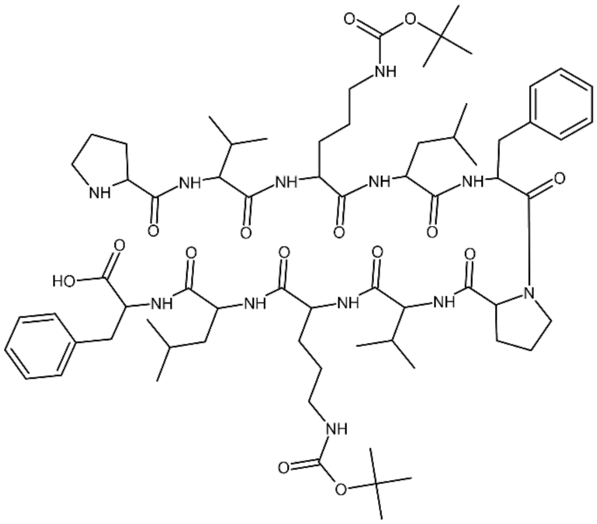
**GS$_C$-L**	**H-Cys-D-Phe-Pro-Val-Orn-Cys-D-Phe-Pro-Val-Orn-N-(2-sulfanylethyl)Gly-NH_2**
	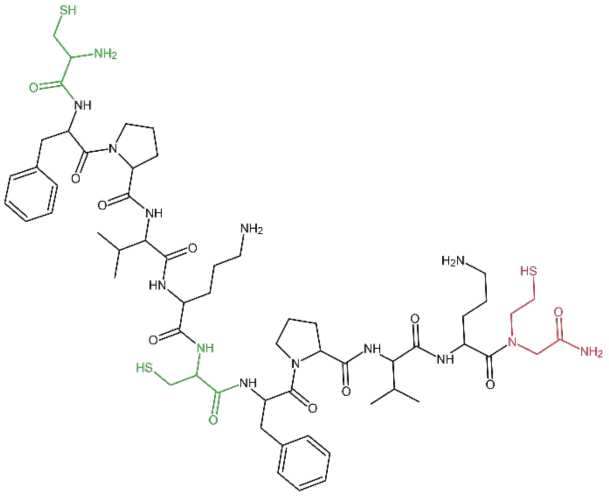

Table S1 (Continuation). List of the obtained peptides.

GS_C	cyclo(-Val-Orn-Cys-D-Phe-Pro-Val-Orn-Cys-D-Phe-Pro-)
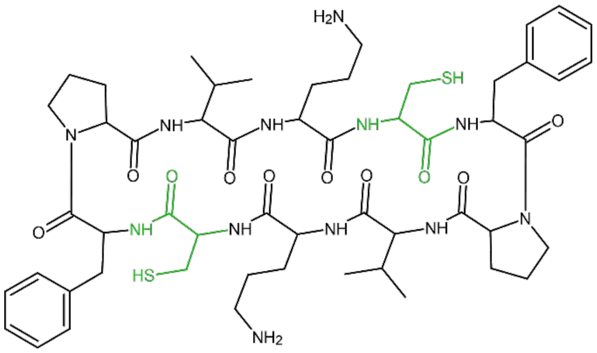	

Table S2. Analytical data for **GS$_C$-FB**.

LC-IT-ToF-MS	Retention time [min]			
	9.3; 8.4; 8.2			
**ESI-MS**	**M**	***m/z***	**Calculated *m/z***	**Ion**
	$C_{60}H_{78}F_4N_{12}O_{10}S_2$	1289.5216	1289.5234	$[M+Na]^+$
		1267.5365	1267.5414	$[M+H]^+$
		645.2633	645.2653	$[M+H+Na]^{2+}$
		634.2693	634.2743	$[M+2H]^{2+}$
**ESI-MS/MS**	**Precursor ion**	**Daughter ions**		
		m/z	**Calculated *m/z***	**Ion**
	634.2693 $[M+2H]^{2+}$	1170.4870	1170.4887	b_{9VP}
		1153.4602	1153.4621	b_{9CO}
		1125.4730	1125.4672	a_{9CO}
		1071.4182	1071.4202	b_{8OV}
		1054.3939	1054.3937	b_{8CO}
		957.3442	957.3409	b_{7CO}
		810.2711	810.2725	b_{7CO}
		620.2800	620.2769	$[M+2H-CO]^{2+}$
		527.7001	527.7005	$[b_{8CO}+H]^{2+}$
		311.2059	311.2078	b_{3PDF}

Table S3. Analytical data for **GS_C-SS**.

LC-IT-ToF-MS	Retention time [min]			
	7.6			
**ESI-MS**	**M**	***m/z***	**Calculated *m/z***	**Ion**
	$C_{54}H_{78}N_{12}O_{10}S_2$	1119.5482	1119.5478	$[M+H]^+$
		560.2799	560.2775	$[M+2H]^{2+}$
**ESI-MS/MS**	**Precursor ion**	**Daughter ions**		
		m/z	**Calculated *m/z***	**Ion**
	560.2799 $[M+2H]^{2+}$	1022.4941	1022.4950	b_{9VP}
		1005.4660	1005.4685	b_{9CO}
		977.4795	977.4736	a_{9CO}
		923.4249	923.4266	b_{8OV}
		906.4013	906.4001	b_{8CO}
		809.3468	809.3473	b_{7CO}
		662.2800	662.2789	b_{6CO}
		546.2874	546.2801	$[M+2H-CO]^{2+}$
		458.2832	458.2762	b_{4DFC}
		453.7076	453.7037	$[b_{8CO}+H]^{2+}$
		405.1791	405.1773	$[b_{7CO}+H]^{2+}$
		344.1990	344.1969	b_{3DFC}
		311.2088	311.2078	b_{3PDF}

Table S4. Analytical data for **GS**.

LC-IT-ToF-MS	Retention time [min]			
	11.0			
**ESI-MS**	**M**	***m/z***	**Calculated *m/z***	**Ion**
	$C_{60}H_{92}N_{12}O_{10}$	1141.7228	1141.7132	$[M+H]^+$
		571.3675	571.3602	$[M+2H]^{2+}$
**ESI-MS/MS**	**Precursor ion**	**Daughter ions**		
		m/z	**Calculated *m/z***	**Ion**
	571.3675 $[M+2H]^{2+}$	1044.6748	1044.6604	b_{9VP}
		1028.6451	1028.6292	b_{9DFL}
		945.6034	945.5920	b_{8OV}
		881.5710	881.5607	b_{8PDF}
		831.5226	831.5127	b_{7LO}
		767.4903	767.4814	b_{7PDF}
		685.4487	685.4396	b_{6OV}
		668.4213	668.4130	b_{6PDF}
		497.8337	497.8260	$[b_{9PDF}+H]^{2+}$
		441.2912	441.2840	$[b_{8PDF}+H]^{2+}$
		424.2963	424.2918	b_{4PDF}
		375.2462	375.2391	b_{3OV}
		311.2126	311.2078	b_{3PDF}
		261.1652	261.1598	b_{2LO}

Table S5. Analytical data for **GS-L**.

LC-IT-ToF-MS	Retention time [min]			
	9.3			
**ESI-MS**	**M**	***m/z***	**Calculated *m/z***	**Ion**
	$C_{60}H_{94}N_{12}O_{11}$	1159.7245	1159.7238	$[M+H]^{+}$
		580.3673	580.3655	$[M+2H]^{2+}$
**ESI-MS/MS**	**Precursor ion**	**Daughter ions**		
		m/z	**Calculated *m/z***	**Ion**
	580.3673 $[M+2H]^{2+}$	1062.6854	1062.671	y_9
		963.6137	963.6026	y_8
		881.5694	881.5607	b_8
		849.5320	849.5233	y_7
		767.4877	767.4814	b_7
		736.4452	736.4392	y_6
		685.4470	685.4396	c_6
		668.4228	668.4130	b_6
		589.3743	589.3708	y_5
		571.3633	571.3602	b_5
		497.8311	497.8260	$[b_9+H]^{2+}$
		482.3091	482.3049	$[y_8+H]^{2+}$
		441.2872	441.2840	$[b_8+H]^{2+}$
		424.2933	424.2918	b_4
		393.2504	393.2496	y_3
		311.2078	311.2078	b_3
		279.1696	279.1703	y_2

LC-MS, ESI-MS, ESI-MS/MS analysis

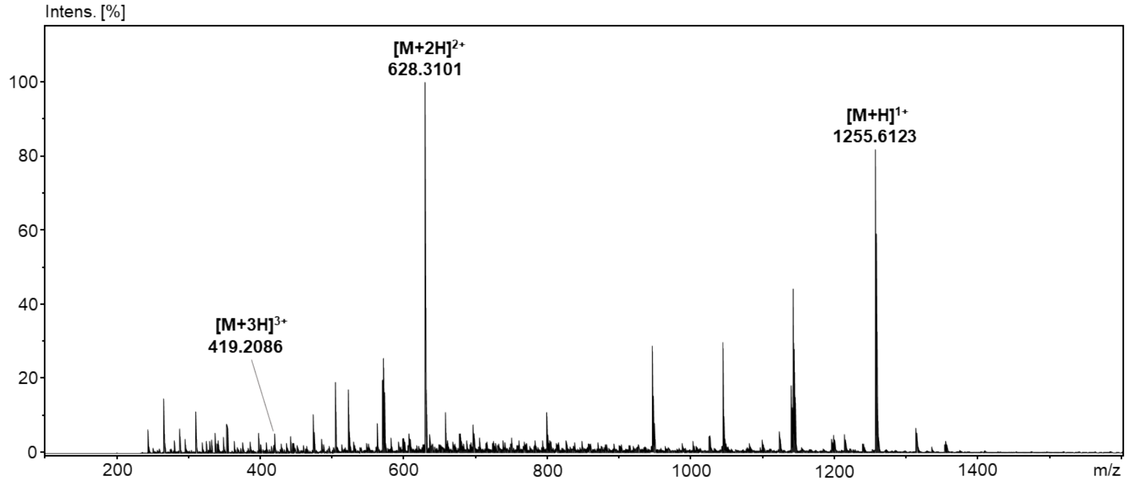

Figure S1. ESI-MS spectrum of crude **GS$_C$-L** (positive ion mode).

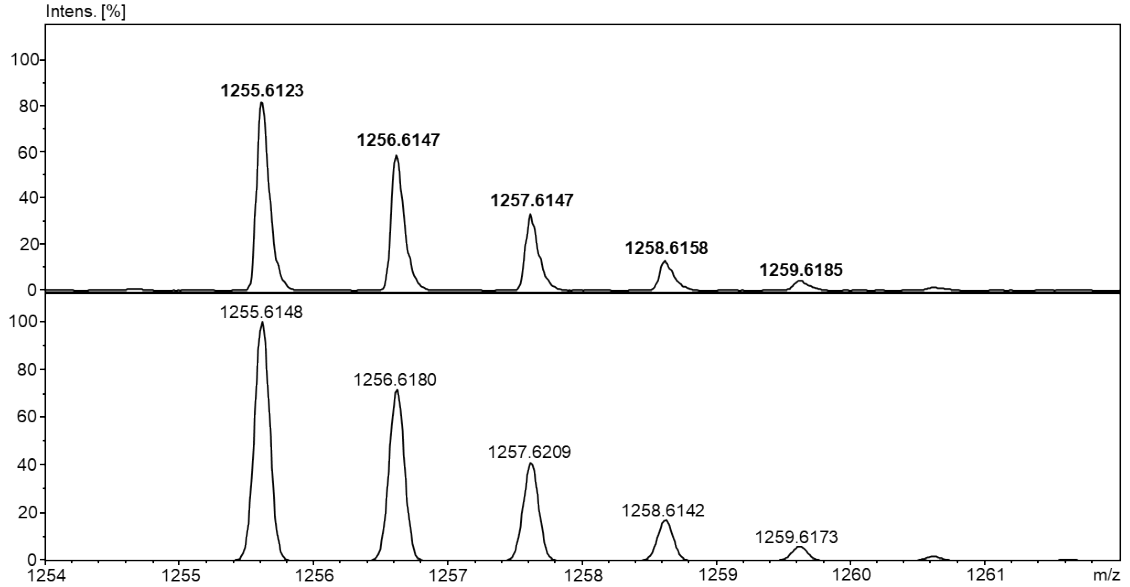

Figure S2. ESI-MS of crude **GS$_C$-L** in zoom range at m/z 1254-1262 (top) and simulated for pseudomolecular ion $[M+H]^+$ where M= $C_{58}H_{90}N_{14}O_{11}S_3$ (bottom).

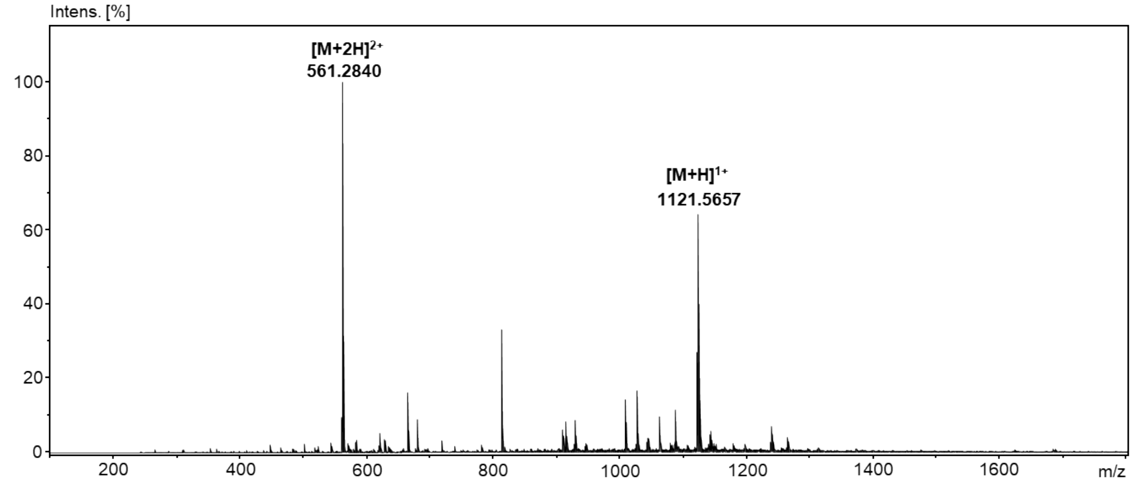

Figure S3. ESI-MS spectrum of crude $\mathbf{GS_C}$ (after cyclization via native chemical ligation); positive ion mode.

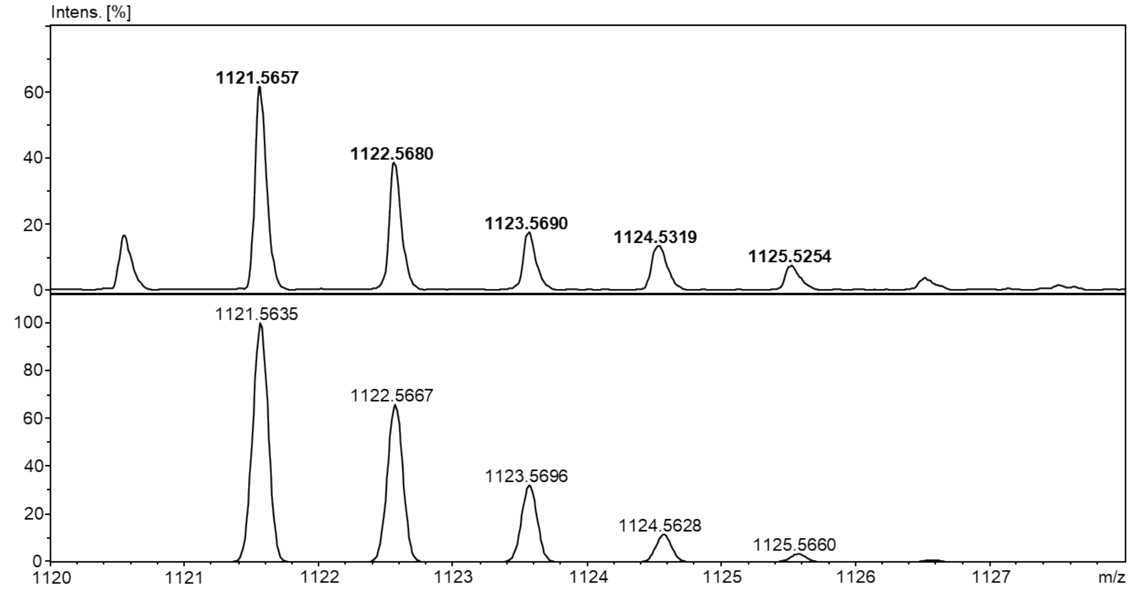

Figure S4. ESI-MS of crude $\mathbf{GS_C}$ (after cyclization via native chemical ligation) in zoom range at m/z 1120-1128 (top) and simulated for pseudomolecular ion $[M+H]^+$ where M = $C_{54}H_{80}N_{12}O_{10}S_2$ (bottom).

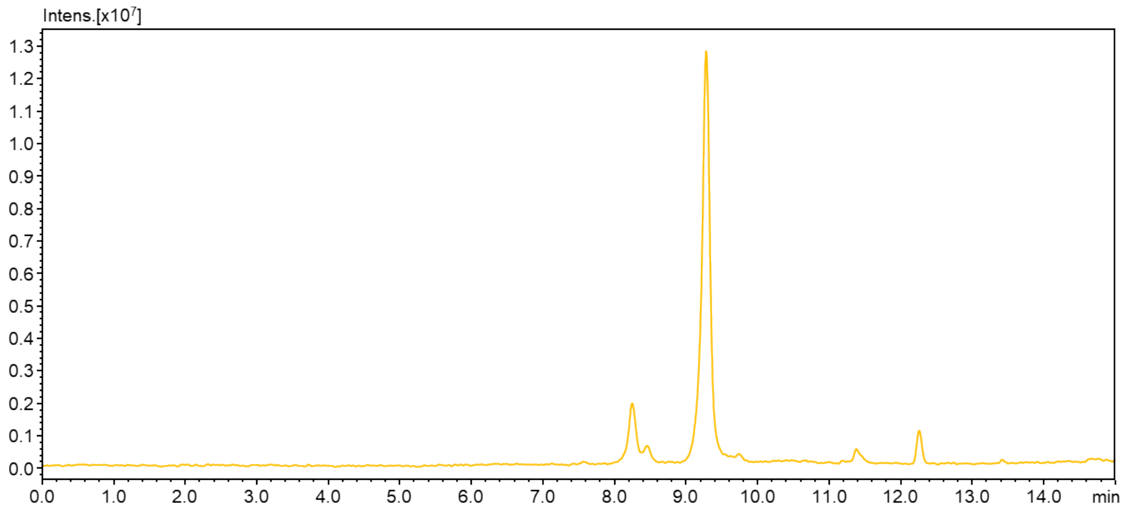

Figure S5. LC-MS (**TIC**) of **GS$_C$-FB**. Conditions: RP-Zorbax column (50 × 2.1 mm, 3.5 µm); gradient elution of 0-80% B in A (A= 0.1% HCOOH in water; B= 0.1% HCOOH in MeCN) in 15 min; flow rate: 0.2 ml/min.

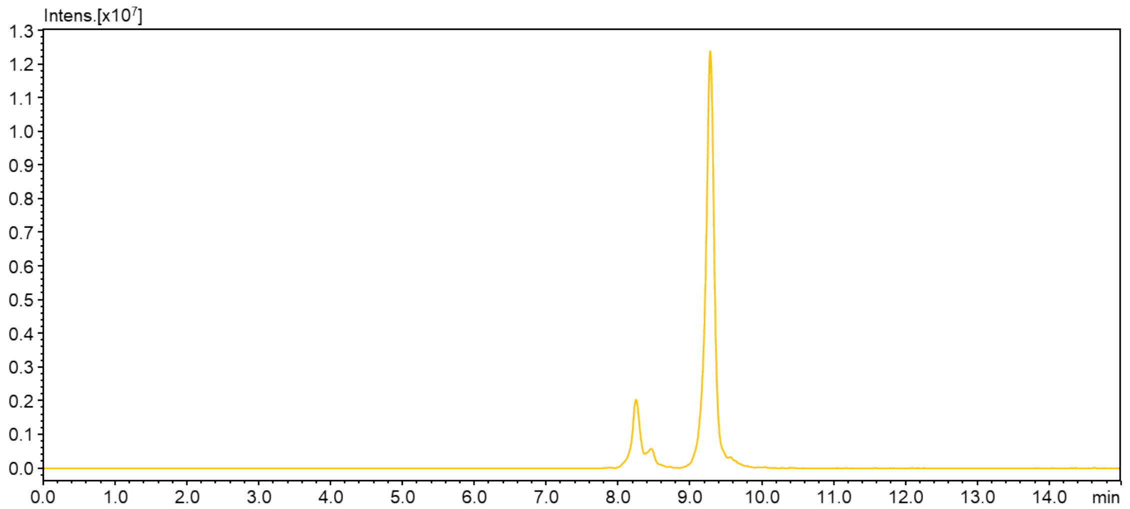

Figure S6. LC-MS (**XIC** for m/z 634.2693) of **GS$_C$-FB**. Conditions: RP-Zorbax column (50 × 2.1 mm, 3.5 µm); gradient elution of 0-80% B in A (A= 0.1% HCOOH in water; B= 0.1% HCOOH in MeCN) in 15 min; flow rate: 0.2 ml/min.

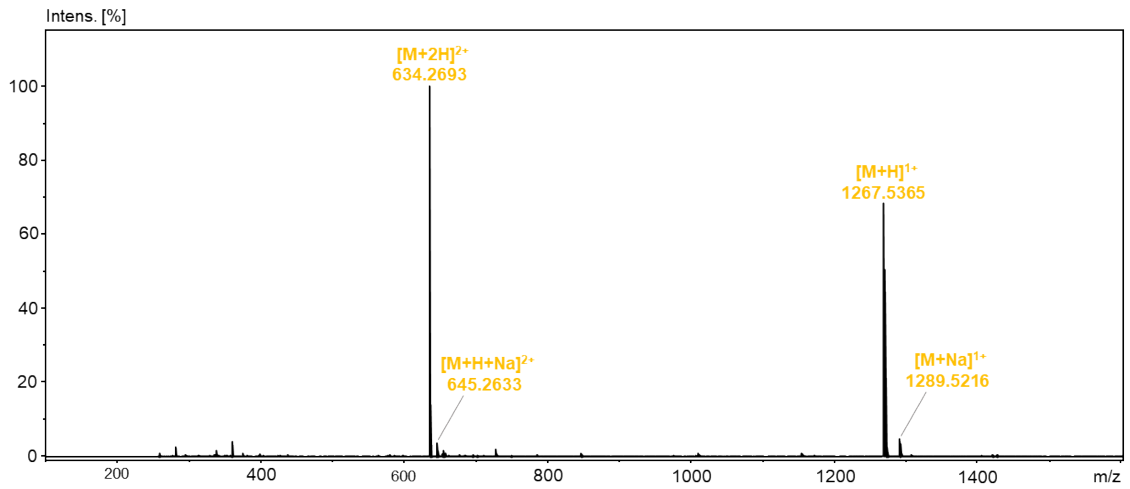

Figure S7. ESI-MS spectrum of **GS$_C$-FB** (positive ion mode).

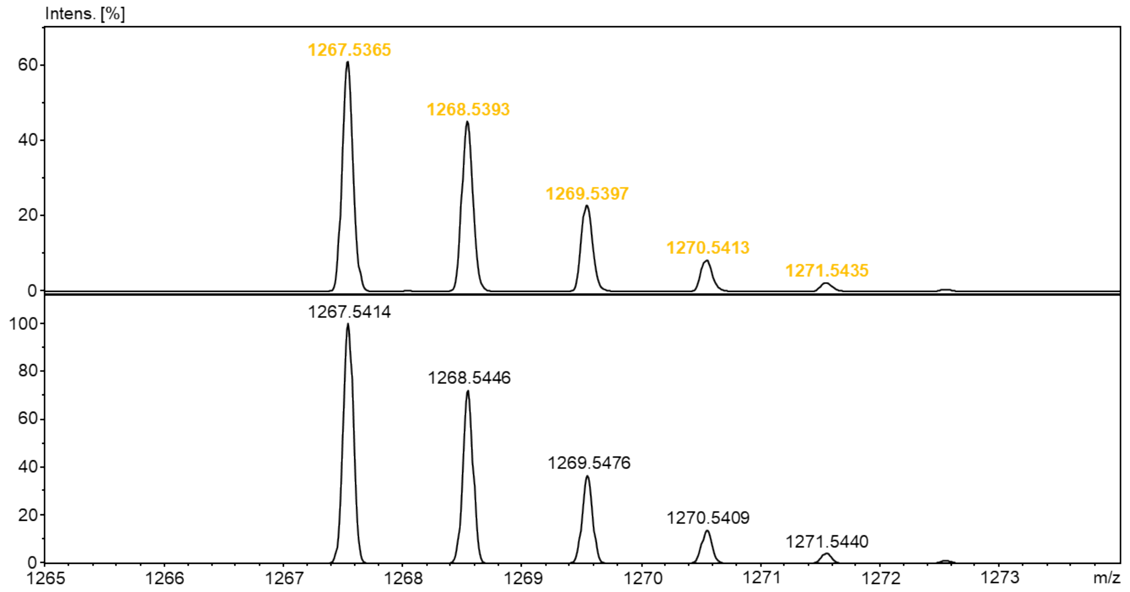

Figure S8. ESI-MS of **GS$_C$-FB** in zoom range at m/z 1265-1274 (top) and simulated for pseudomolecular ion $[M+H]^+$ where M = $C_{60}H_{78}F_4N_{12}O_{10}S_2$ (bottom).

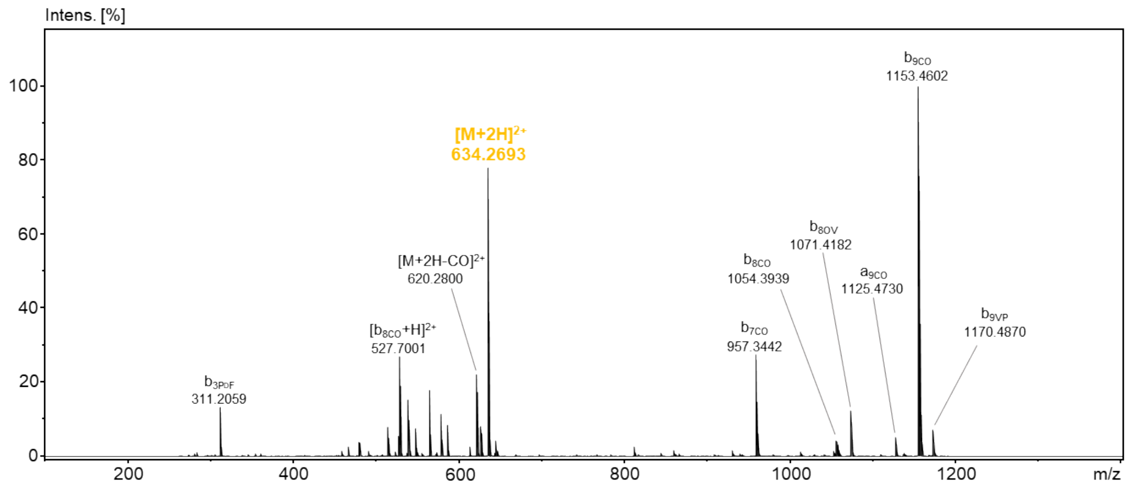

Figure S9. ESI-MS/MS spectrum of **GS$_C$-FB** for precursor ion m/z 634.2693 (positive ion mode; collision energy: 20 eV).

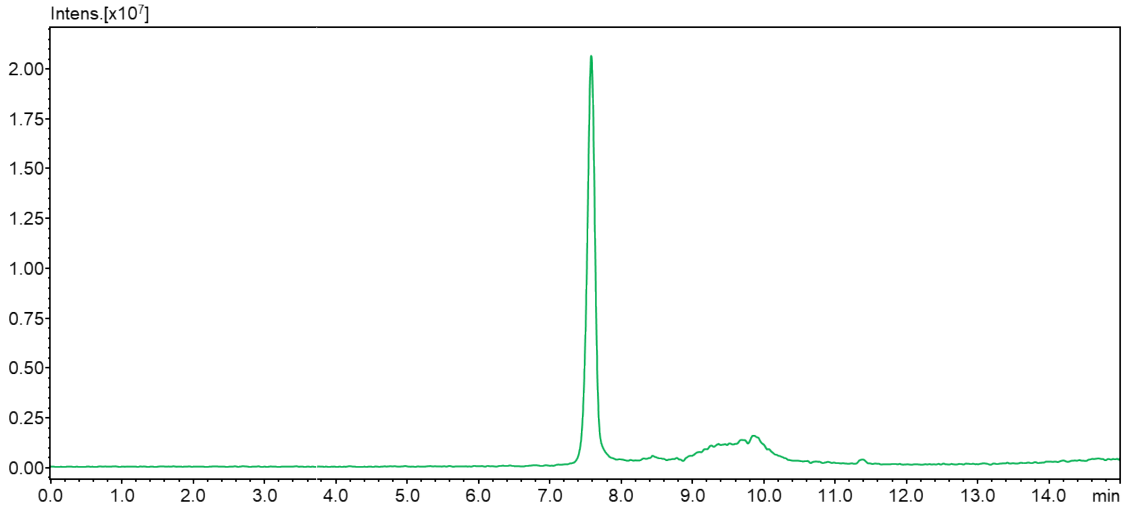

Figure S10. LC-MS (**TIC**) of **GS$_C$-SS**. Conditions: RP-Zorbax column (50 × 2.1 mm, 3.5 µm); gradient elution of 0-80% B in A (A= 0.1% HCOOH in water; B= 0.1% HCOOH in MeCN) in 15 min; flow rate: 0.2 ml/min.

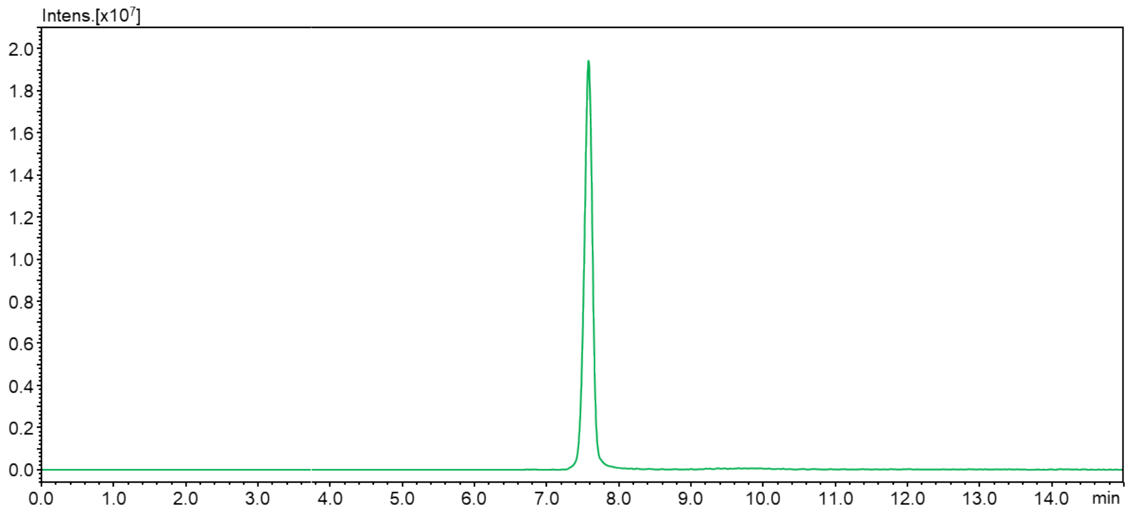

Figure S11. LC-MS (**XIC** for *m/z* 560.2799) of **GS$_C$-SS**. Conditions: RP-Zorbax column (50 × 2.1 mm, 3.5 µm); gradient elution of 0-80% B in A (A= 0.1% HCOOH in water; B= 0.1% HCOOH in MeCN) in 15 min; flow rate: 0.2.

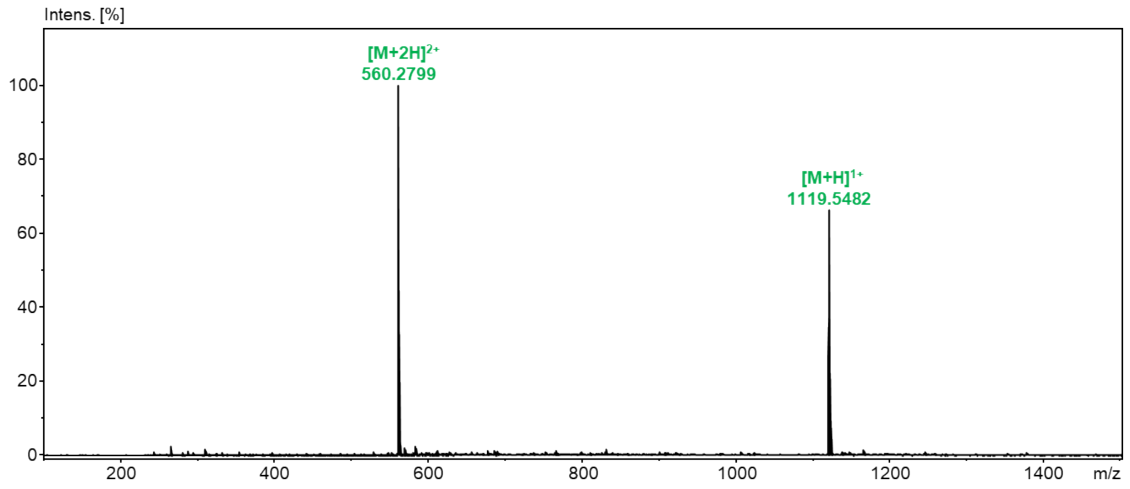

Figure S12. ESI-MS spectrum of **GS$_C$-SS** (positive ion mode).

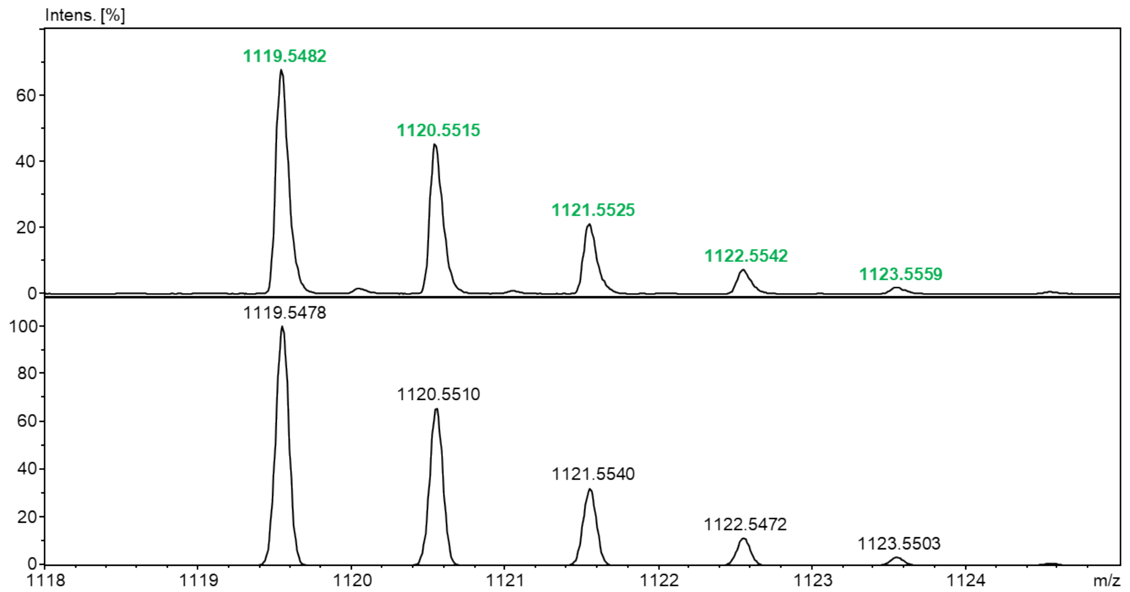

Figure S13. ESI-MS of GS$_C$-SS in zoom range at m/z 1118-1125 (top) and simulated for pseudomolecular ion $[M+H]^+$ where M = $C_{54}H_{78}N_{12}O_{10}S_2$ (bottom).

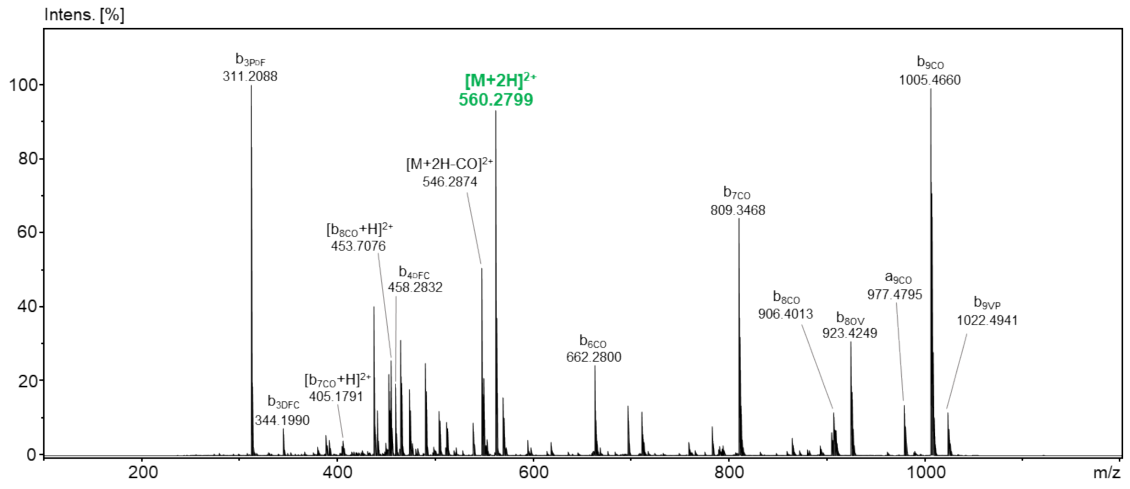

Figure S14. ESI-MS/MS spectrum of GS$_C$-SS for precursor ion m/z 560.2799 (positive ion mode; collision energy: 20 eV).

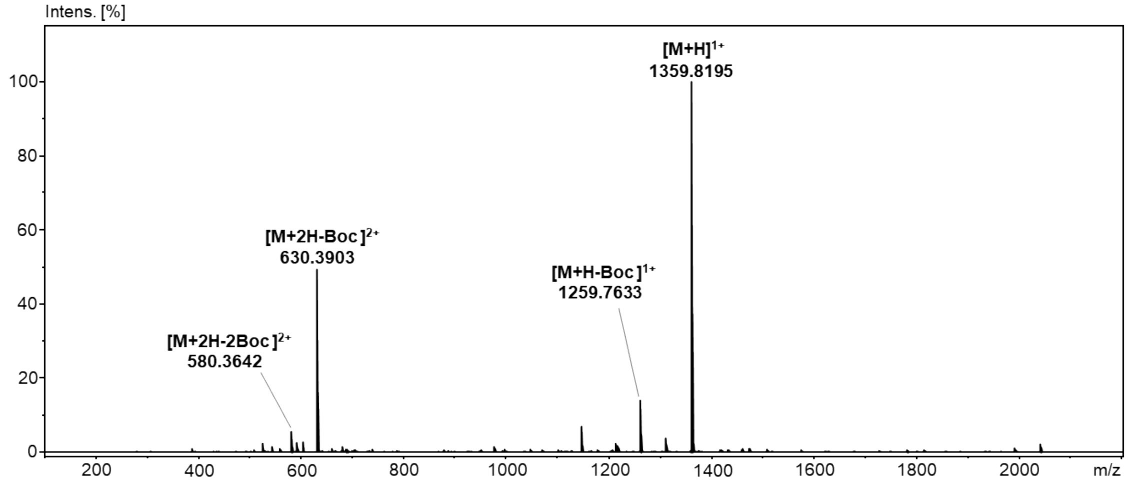

Figure S15. ESI-MS spectrum of crude **GS-L(2Boc)** (positive ion mode).

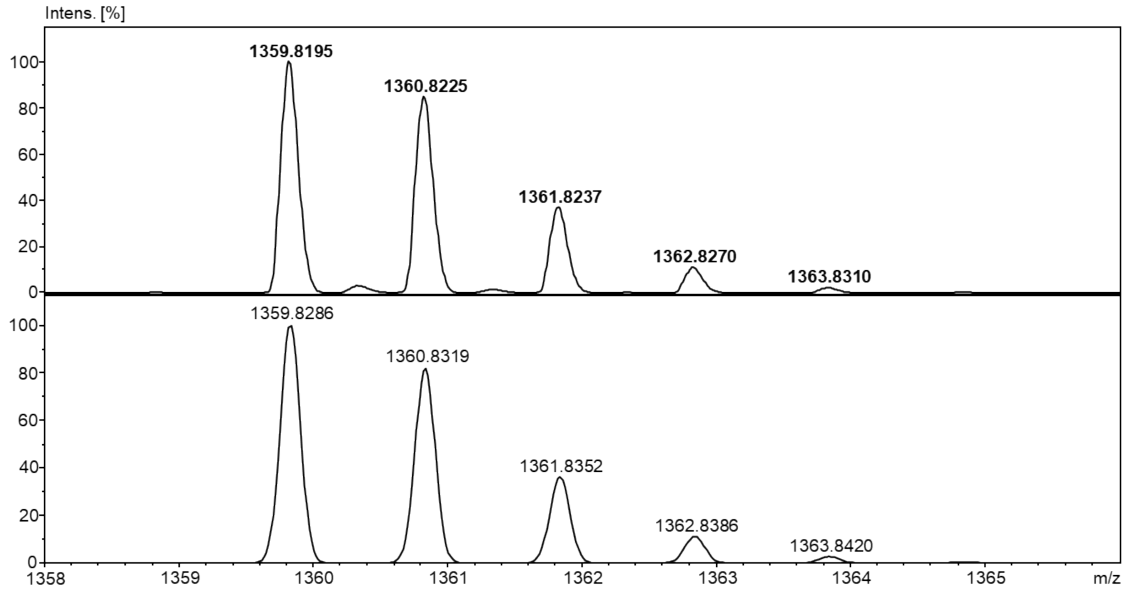

Figure S16. ESI-MS of crude **GS-L(2Boc)** in zoom range at m/z 1358-1366 (top) and simulated for pseudomolecular ion $[M+H]^+$ where M = $C_{70}H_{110}N_{12}O_{15}$ (bottom).

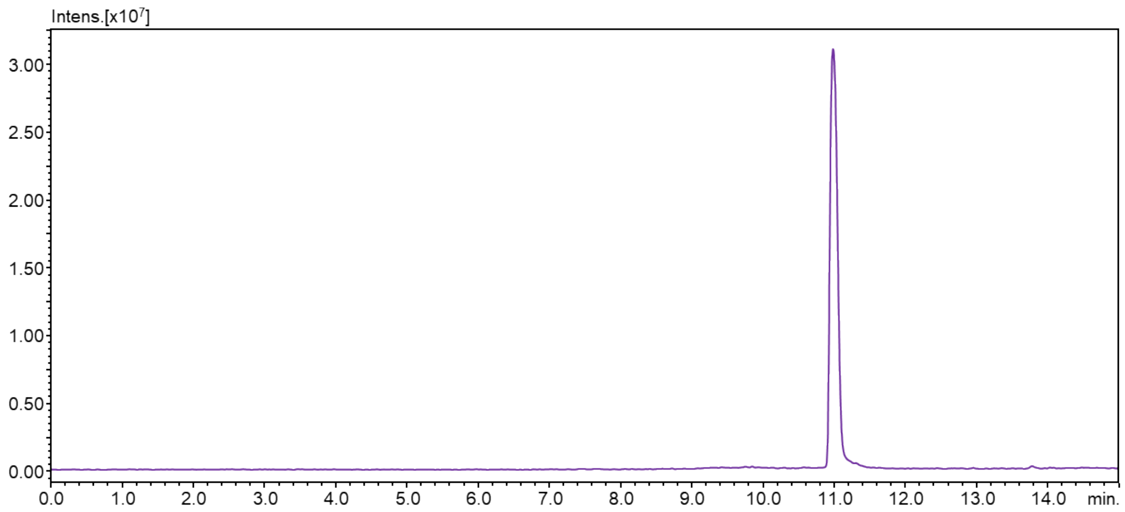

Figure S17. LC-MS (TIC) of **GS**. Conditions: RP-Zorbax column (50 × 2.1 mm, 3.5 µm); gradient elution of 0-80% B in A (A= 0.1% HCOOH in water; B= 0.1% HCOOH in MeCN) in 15 min; flow rate: 0.2 ml/min.

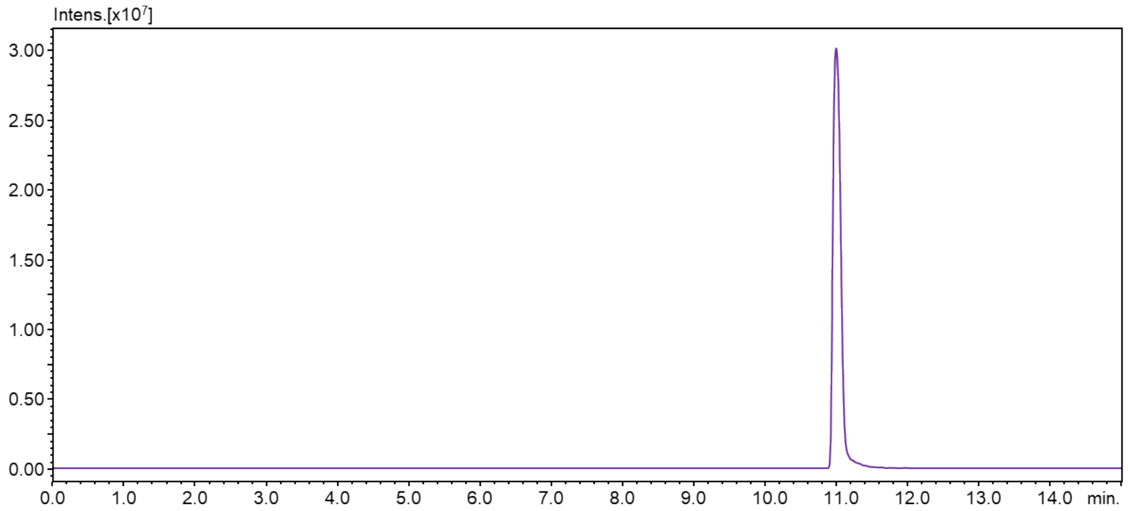

Figure S18. LC-MS (XIC for m/z 571.3675) of **GS**. Conditions: RP-Zorbax column (50 × 2.1 mm, 3.5 µm); gradient elution of 0-80% B in A (A= 0.1% HCOOH in water; B= 0.1% HCOOH in MeCN) in 15 min; flow rate: 0.2 ml/min.

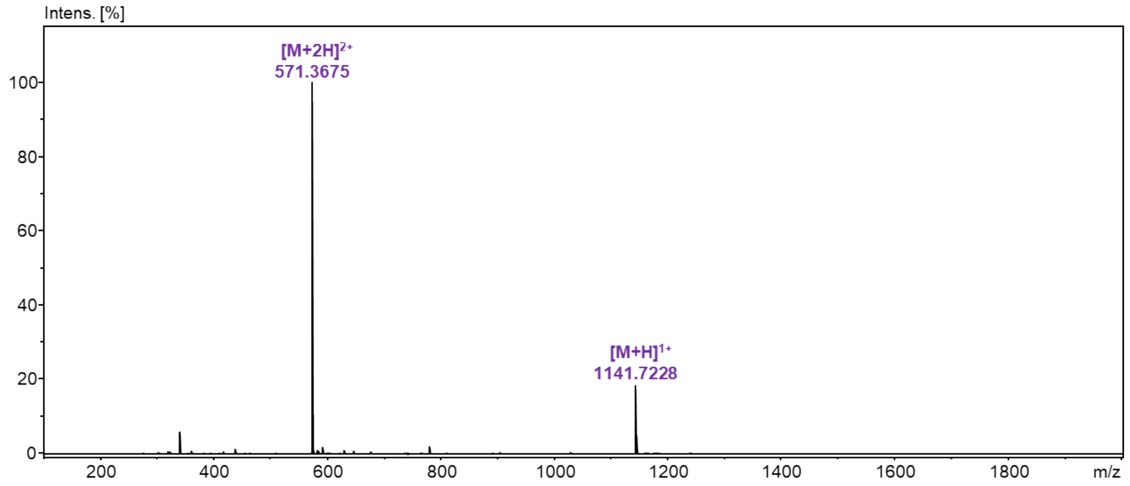

Figure S19. ESI-MS spectrum of **GS** (positive ion mode).

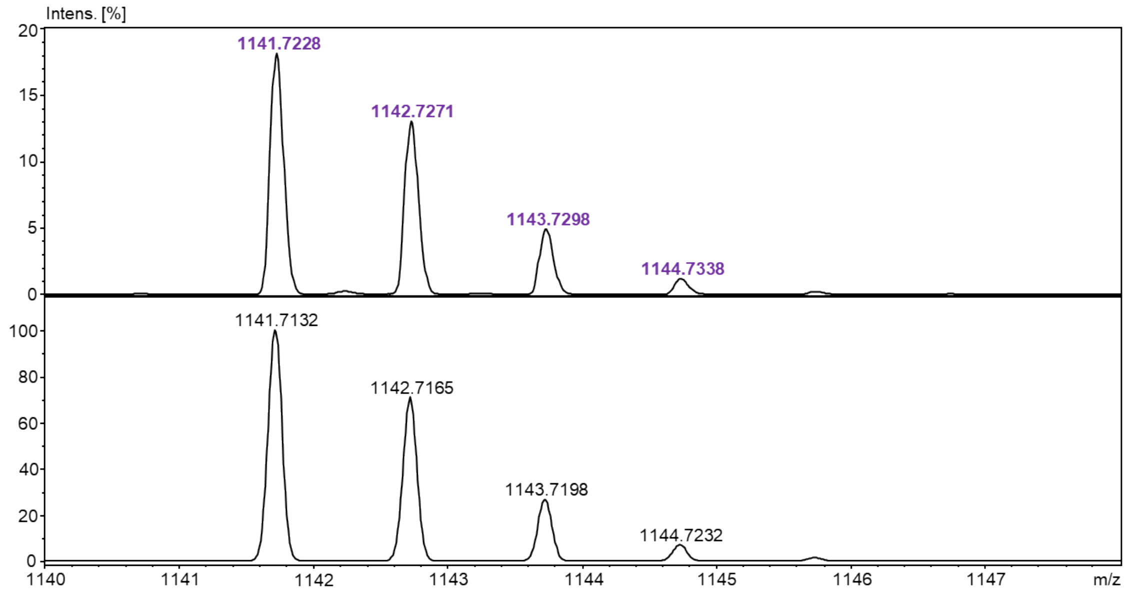

Figure S20. ESI-MS of **GS** in zoom range at m/z 1140-1148 (top) and simulated for pseudomolecular ion $[M+H]^+$ where M = $C_{60}H_{92}N_{12}O_{10}$ (bottom).

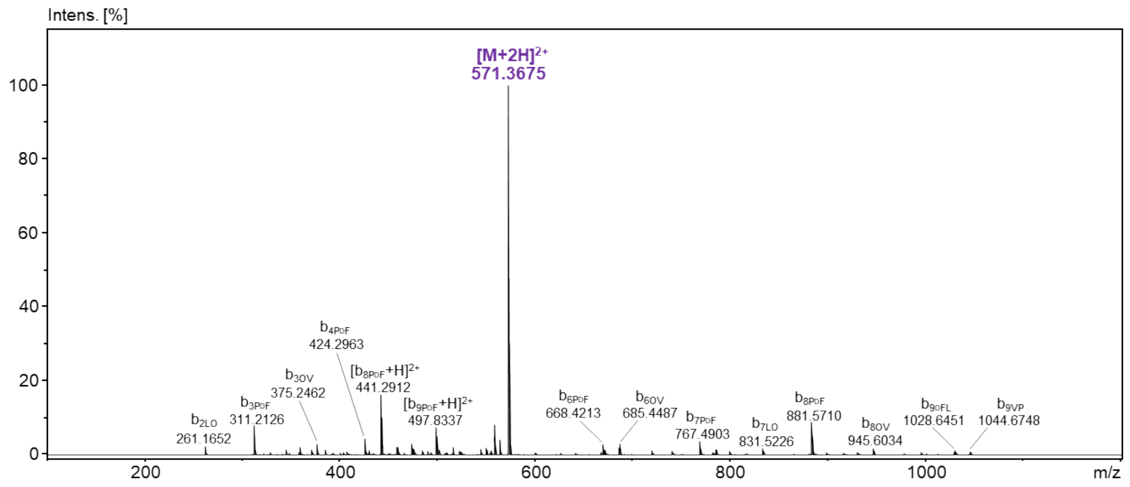

Figure S21. ESI-MS/MS spectrum of **GS** for precursor ion m/z 571.3675 (positive ion mode; collision energy: 25 eV).

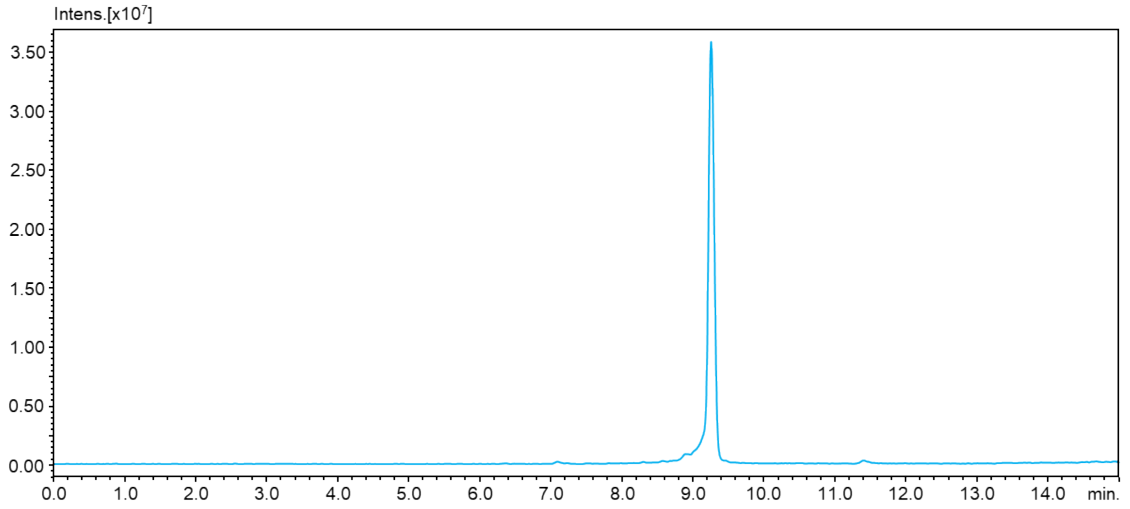

Figure S22. LC-MS (TIC) of **GS-L**. Conditions: RP-Zorbax column (50 × 2.1 mm, 3.5 µm); gradient elution of 0-80% B in A (A= 0.1% HCOOH in water; B= 0.1% HCOOH in MeCN) in 15 min; flow rate: 0.2 ml/min.

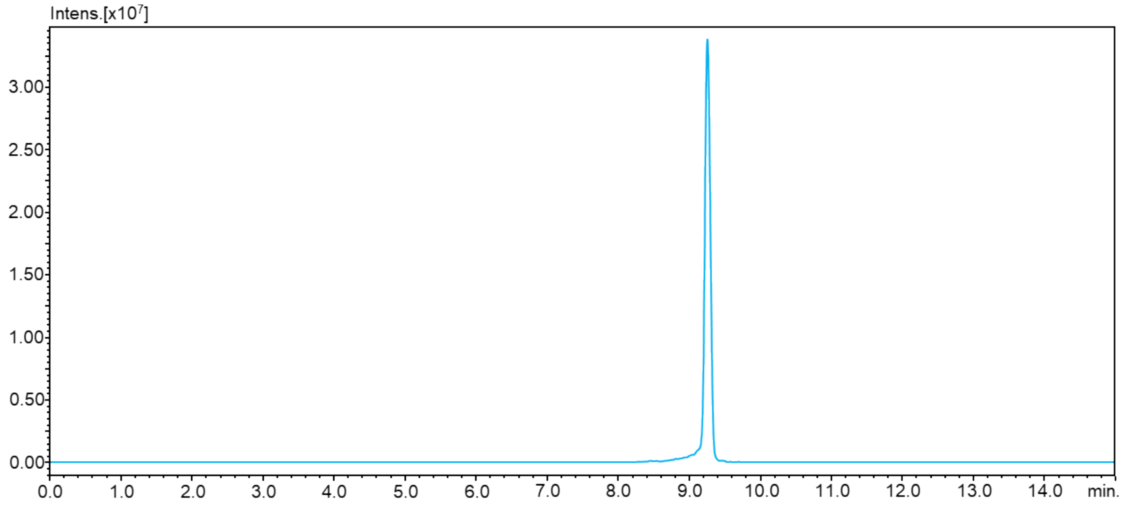

Figure S23. LC-MS (XIC for m/z 580.3673) of **GS-L**. Conditions: RP-Zorbax column (50 × 2.1 mm, 3.5 µm); gradient elution of 0-80% B in A (A= 0.1% HCOOH in water; B= 0.1% HCOOH in MeCN) in 15 min; flow rate: 0.2 ml/min.

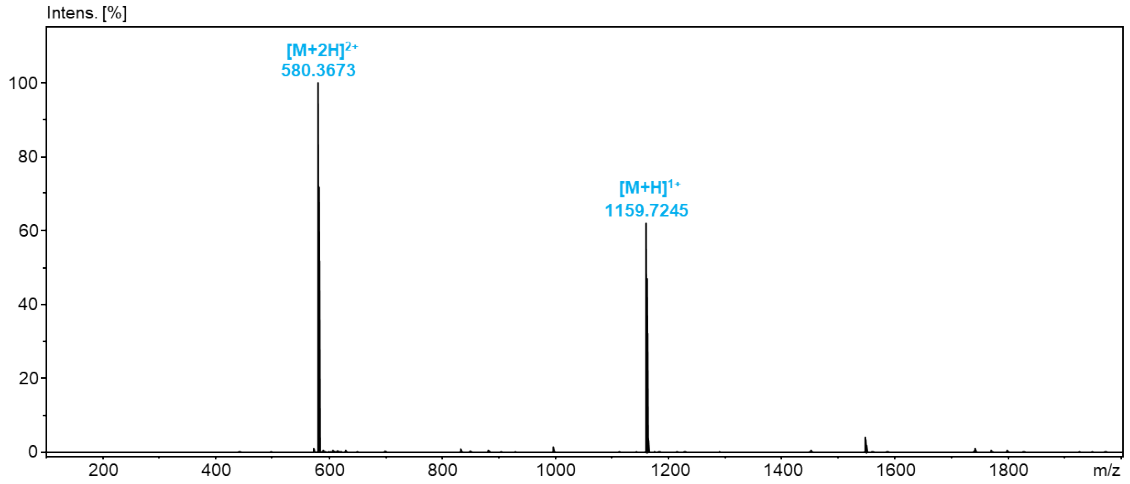

Figure S24. ESI-MS spectrum of **GS-L** (positive ion mode).

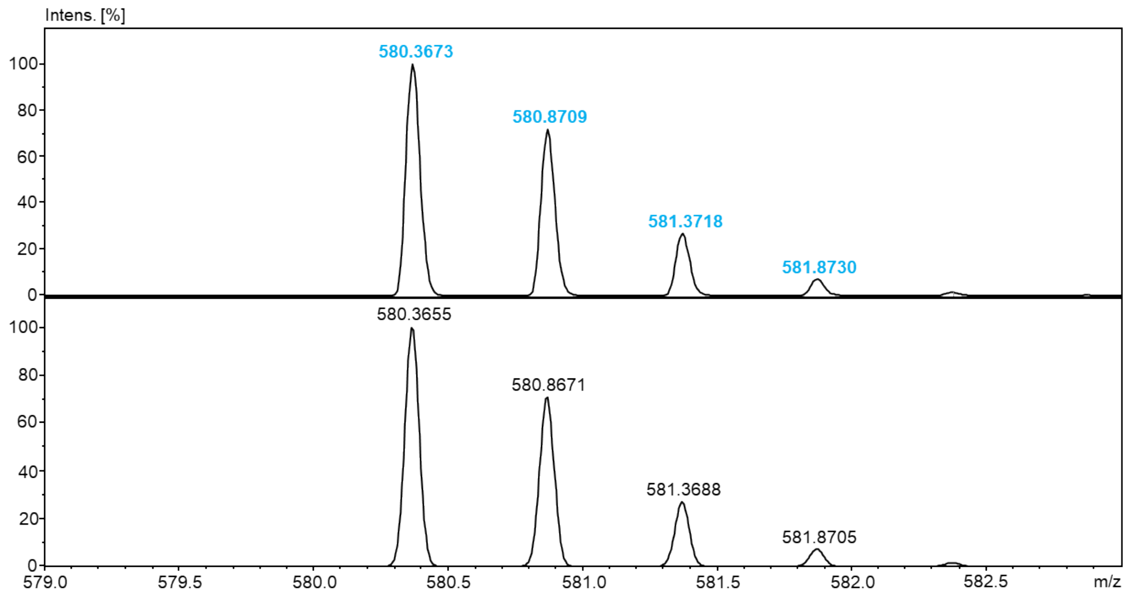

Figure S25. ESI-MS of GS-L in zoom range at m/z 579-583 (top) and simulated for pseudomolecular ion $[M+2H]^{2+}$ where M = $C_{60}H_{94}N_{12}O_{11}$ (bottom).

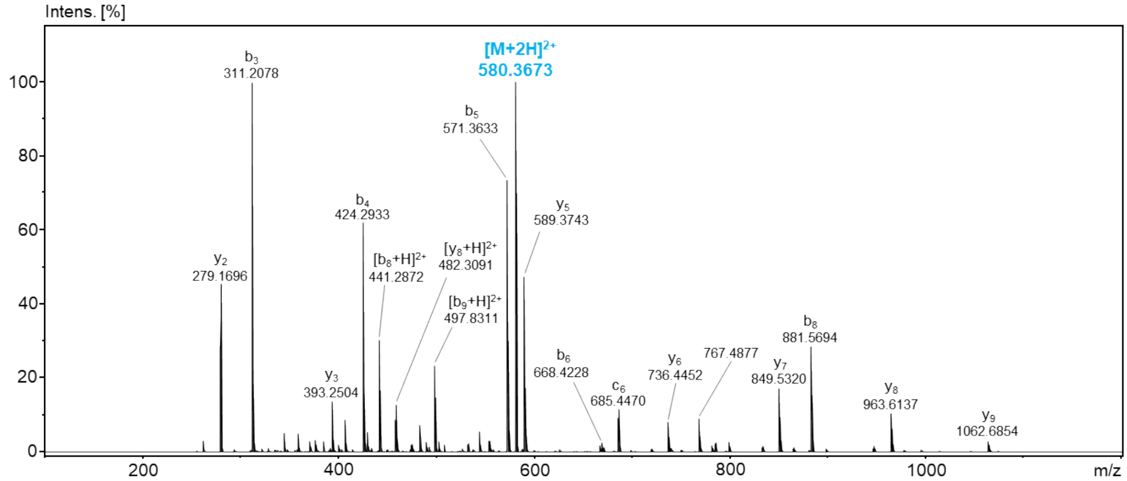

Figure S26. ESI-MS/MS spectrum of GS-L for precursor ion m/z 580.3673 (positive ion mode; collision energy: 25 eV).

HPLC-DAD analysis

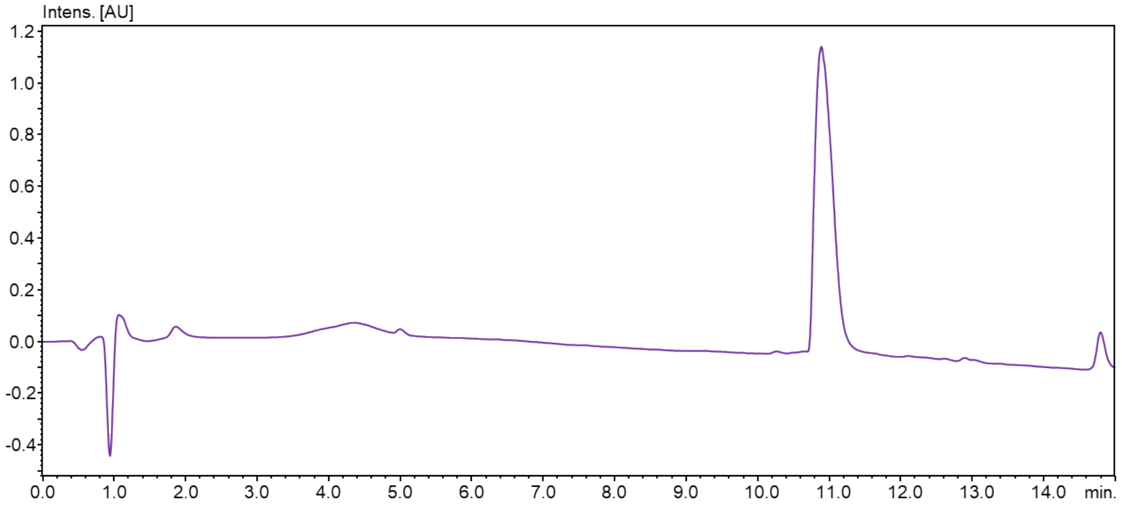

Figure S27. HPLC-DAD chromatogram of **GS**. Conditions: RP-Zorbax column (50 × 2.1 mm, 3.5 µm); gradient elution of 0-80% B in A (A = 0.1% HCOOH in water; B = 0.1% HCOOH in MeCN) in 15 min; flow rate: 0.2 ml/min; detection – DAD, 210 nm.

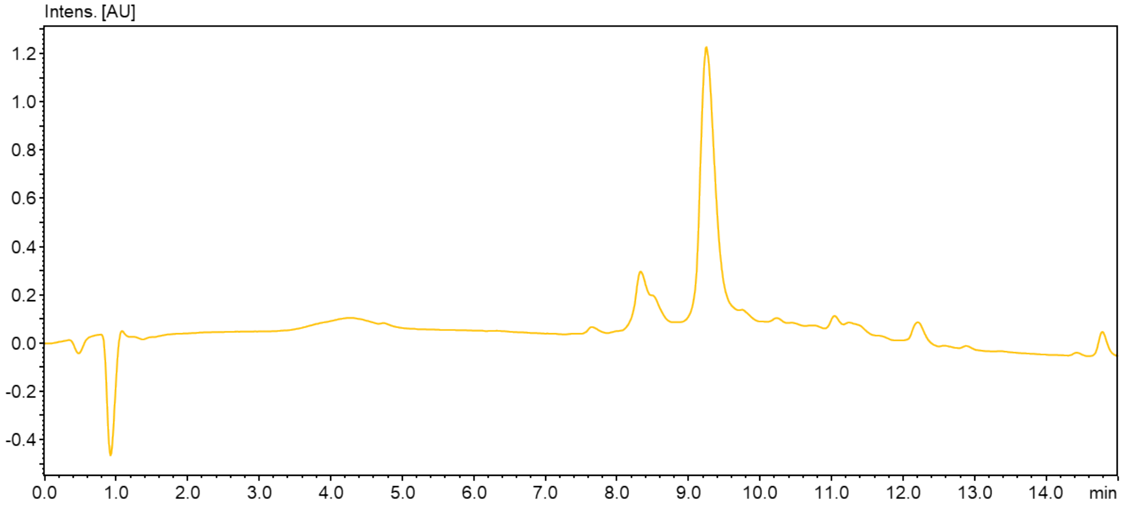

Figure S28. HPLC-DAD chromatogram of GS_C-FB. Conditions: RP-Zorbax column (50 × 2.1 mm, 3.5 µm); gradient elution of 0-80% B in A (A = 0.1% HCOOH in water; B = 0.1% HCOOH in MeCN) in 15 min; flow rate: 0.2 ml/min; detection – DAD, 210 nm.

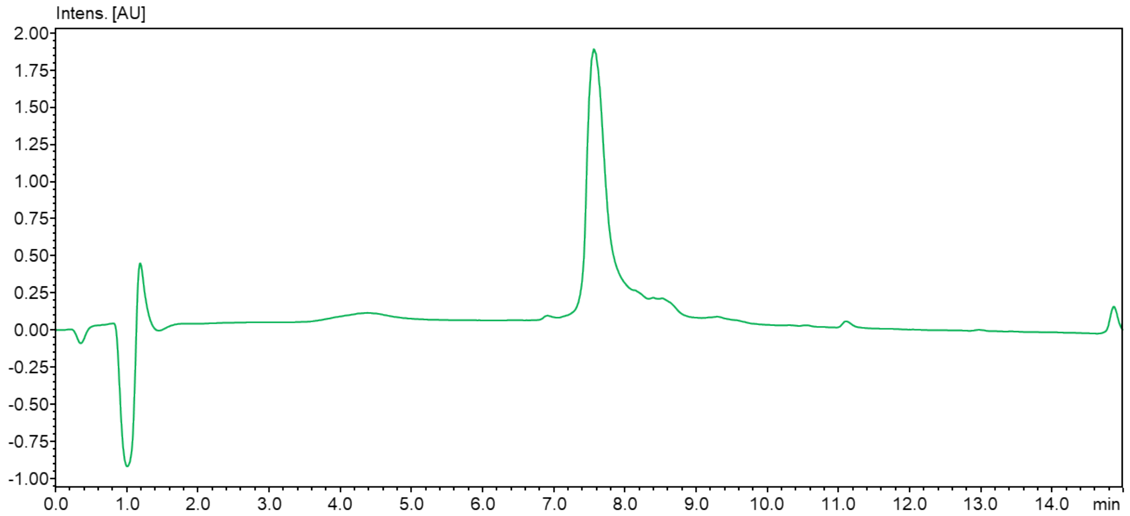

Figure S29. HPLC-DAD chromatogram of **GS_C-SS**. Conditions: RP-Zorbax column (50 × 2.1 mm, 3.5 μm); gradient elution of 0-80% B in A (A = 0.1% HCOOH in water; B = 0.1% HCOOH in MeCN) in 15 min; flow rate: 0.2 ml/min; detection – DAD, 210 nm.

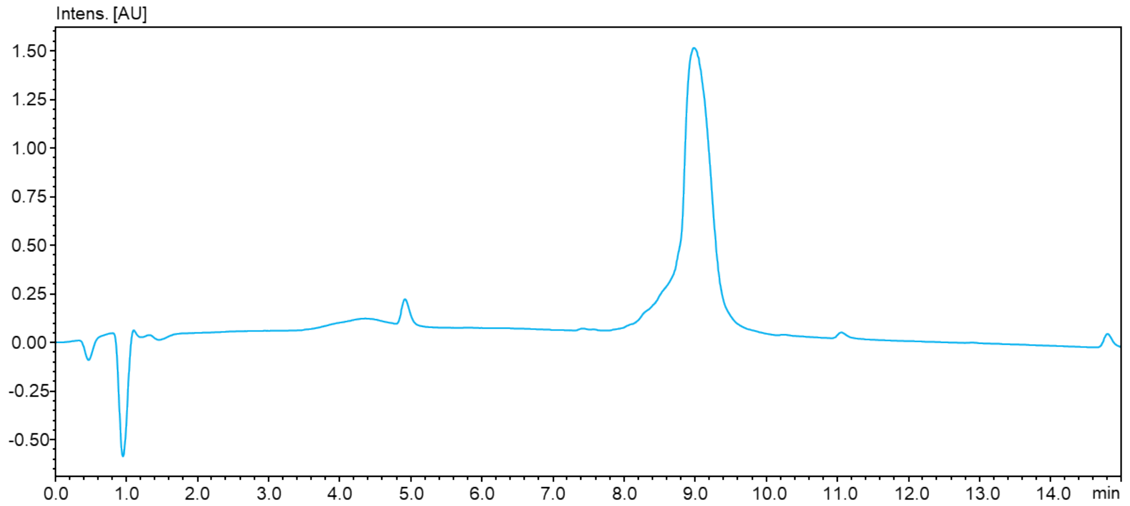

Figure S30. HPLC-DAD chromatogram of **GS-L**. Conditions: RP-Zorbax column (50 × 2.1 mm, 3.5 μm); gradient elution of 0-80% B in A (A = 0.1% HCOOH in water; B = 0.1% HCOOH in MeCN) in 15 min; flow rate: 0.2 ml/min; detection – DAD, 210 nm.

Molecular modeling

Density Functional Theory Investigation of GS and its Analogs

In Figures S31-S34 the structures of the studied peptides obtained as a result of the DFT/M06-2X/def2-TZVP simulations with IEF-PCM and water as a solvent are presented. The prepared models provided information on the structure complexity and possible inter-residue hydrogen bonding formation. We focused on the hydrogen bonds network based on geometry criteria to visualize the strongest and the most significant among non-covalent bonds in the biologically relevant systems. However, it is worth to mention that besides hydrogen bonds other types of non-covalent interactions are present, e.g. van der Waals, which have an attractive nature. Taking into account that inside the peptides we were able to detect such interactions, we can conclude that they play a stabilizing role in their conformations. The obtained results are presented in Tables S6, S7, S8, and the discussion of the relevant interactions follows the Table S8. It is worth mentioning that concerning GS the obtained theoretical results were compared with experimental data available – crystal structure of gramicidin S hydrochloride [1] – and general agreement between the two was found. Additionally, other levels of theory were benchmarked to make our choice for further computational studies reasonable and supported by the good agreement between experimental and theoretical data (see Table S7).

Color coding	
carbon – grey<br>nitrogen – blue<br>oxygen – red	sulfur – yellow<br>fluorine – green<br>hydrogen – white

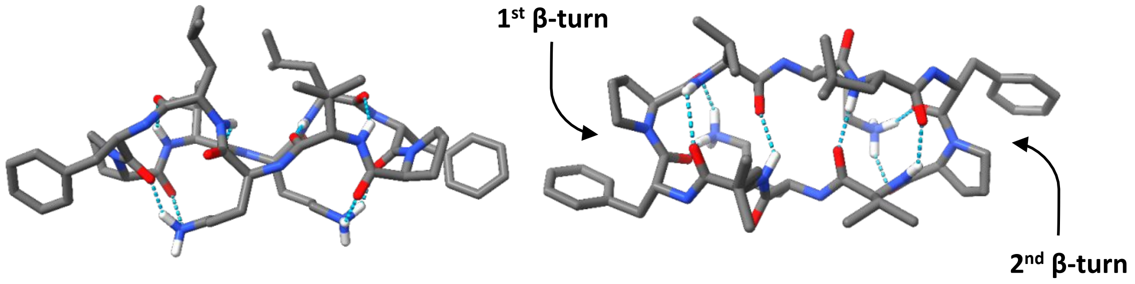

Figure S31. GS structure optimized at the M06-2X/def2-TZVP level of theory with solvent reaction field (IEF-PCM, water as a solvent). Side view (left) and top view (right). Hydrogen atoms not involved in the hydrogen bond formation are omitted for clarity. Dotted cyan lines indicate the presence of intramolecular hydrogen bonds.

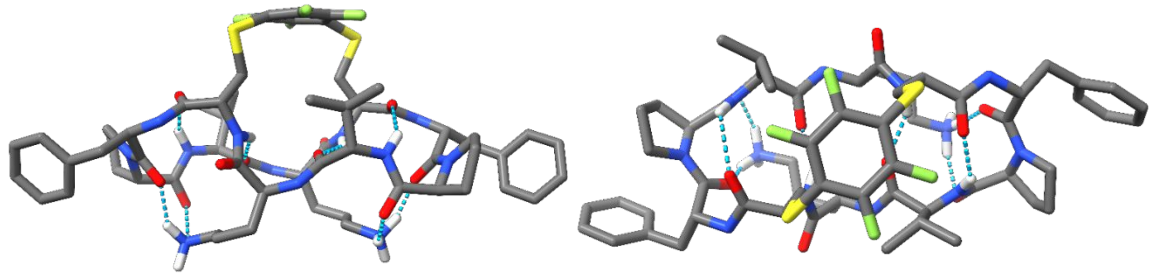

Figure S32. GS_C-FB structure optimized at the M06-2X/def2-TZVP level of theory with solvent reaction field (IEF-PCM, water as a solvent). Side view (left) and top view (right). Hydrogen atoms not involved in the hydrogen bond formation are omitted for clarity. Dotted cyan lines indicate the presence of intramolecular hydrogen bonds.

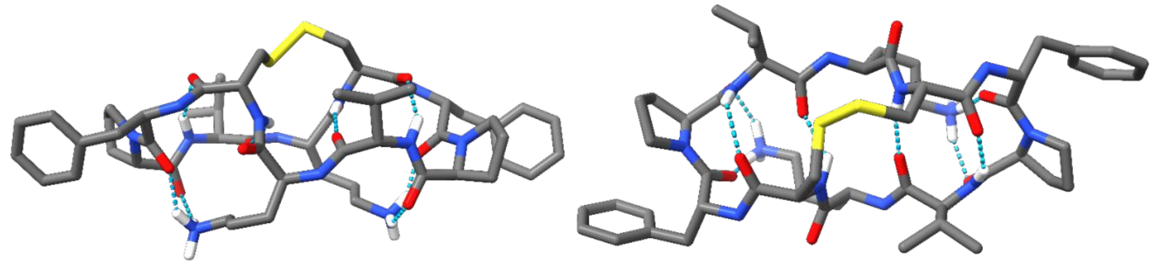

Figure S33. GS_C-SS structure optimized at the M06-2X/def2-TZVP level of theory with solvent reaction field (IEF-PCM, water as a solvent). Side view (left) and top view (right). Hydrogen atoms not involved in the hydrogen bond formation are omitted for clarity. Dotted cyan lines indicate the presence of intramolecular hydrogen bonds.

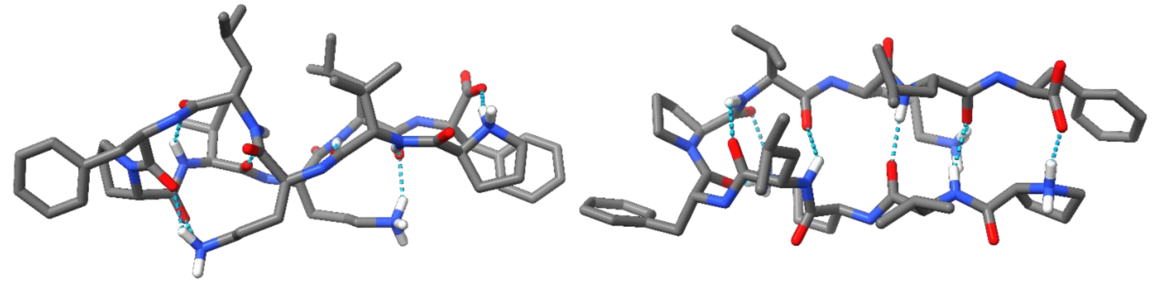

Figure S34. GS-L structure optimized on M06-2X/def2-TZVP level of theory with solvent reaction field (IEF-PCM, water as a solvent). Side view (left) and top view (right). Hydrogen atoms not involved in the hydrogen bond formation are omitted for clarity. Dotted cyan lines indicate the presence of intramolecular hydrogen bonds.

Table S6. Dihedral angle values obtained at the M06-2X/def2-TZVP level of theory with solvent reaction field (IEF-PCM, water as a solvent) for GS, GS_C-FB, GS_C-SS, and GS-L structures.

		**Dihedral angle value [°]**			
		**GS**	**GS_C-FB**	**GS_C-SS**	**GS-L**
**1st β-turn**	φ_{i+1}	59.1	60.9	63.5	57.2
	ψ_{i+1}	-131.5	-131.9	-131.0	-131.5
	φ_{i+2}	-69.7	-70.6	-69.7	-69.9
	ψ_{i+2}	-35.0	-35.1	-34.7	-33.1
**2nd β-turn**	φ_{i+1}	56.1	53.3	57.1	NA
	ψ_{i+1}	-132.1	-135.9	-133.4	NA
	φ_{i+2}	-71.1	-72.7	-68.6	NA
	ψ_{i+2}	-25.1	-23.1	-27.9	NA

Table S7. Selected hydrogen bonds present in GS obtained at different levels of theory in the gas phase and compared with the literature crystal structure. The calculations were performed using various functionals and def2-TZVP basis set.

Methods	Donor	Acceptor	HB distance [Å]			HB angle [°]
			N-H	O...H	N…O	
**Crystal structure [Ref. 1]**	Val I	Leu II	0.8604	2.4461	3.2957	169.5
	Leu II	Val I	0.8606	2.0593	2.9179	175.3
	Leu I	Val II	0.8600	1.9122	2.7643	170.6
	Val II	Leu I	0.8595	2.2108	3.0353	160.7
**M06-2X**	Val I	Leu II	1.0157	2.2481	3.1711	150.4
	Leu II	Val I	1.0147	2.0511	2.9161	141.7
	Leu I	Val II	1.0178	1.8994	2.8948	165.2
	Val II	Leu I	1.0151	2.0766	2.9644	144.8
**B3LYP**	Val I	Leu II	1.0119	2.3486	3.2332	145.4
	Leu II	Val I	1.0144	2.0792	3.0556	160.9
	Leu I	Val II	1.0155	2.0055	2.9917	163.1
	Val II	Leu I	1.0131	2.2036	3.0878	144.9
**PBE0**	Val I	Leu II	1.0126	2.2747	3.1835	148.7
	Leu II	Val I	1.0144	2.0169	2.9843	158.6
	Leu I	Val II	1.0159	1.9551	2.9454	164.1
	Val II	Leu I	1.0137	2.1513	3.0433	145.8
**ωB97-XD**	Val I	Leu II	1.0121	2.2706	3.1930	150.9
	Leu II	Val I	1.0131	1.9779	2.8986	149.8
	Leu I	Val II	1.0150	1.8234	2.8177	165.4
	Val II	Leu I	1.0133	2.1001	3.0135	148.9

Reference:

[1] Asano, A.; Doi, M. Crystal structure of gramicidin s hydrochloride at 1.1 Å Resolution. *X-ray Structure Analysis Online* **2019**, *35* (0), 1–2. DOI: 10.2116/xraystruct.35.1

Table S8. Selected hydrogen bonds present in GS and its analogs. The data was obtained as a result of the DFT/M06-2X/def2-TZVP with IEF-PCM and water as a solvent. The hydrogen bond energy was estimated using data from the QTAIM and based on EML equation. Donor-acceptor distances in parentheses for GS are experimental values [Ref. 1].

Compound	Donor	Acceptor	HB distance [Å]			HB angle [°]	HB Energy [kcal/mol]
			N-H	H…O	N…O		
**GS**	Val I	Leu II	1.0138	2.4473	3.3563 (3.296)	148.9	-1.3036
	Leu II	Val I	1.0153	2.0156	2.8816 (2.918)	141.7	-4.8202
	Leu I	Val II	1.0174	1.8108	2.8189 (2.764)	170.4	-8.9398
	Val II	Leu I	1.0154	2.1031	3.0351 (3.035)	151.6	-3.3895
**GS$_C$-FB**	Val I	Cys II	1.0129	2.5955	3.4884	146.9	-0.9362
	Cys II	Val I	1.0167	1.9441	2.8545	147.5	-5.8870
	Cys I	Val II	1.0198	1.9265	2.9264	166.0	-5.9786
	Val II	Cys I	1.0140	2.1152	3.0150	146.8	-3.3565
**GS$_C$-SS**	Val I	Cys II	1.0126	2.6811	3.5777	147.6	-0.7662
	Cys II	Val I	1.0154	2.1577	2.9541	133.9	-3.3578
	Cys I	Val II	1.0149	1.8051	2.7914	163.0	-9.1146
	Val II	Cys I	1.0148	2.1380	3.0598	150.1	-3.0676
**GS-L**	Val I	Leu II	1.0147	2.3083	3.2298	150.4	-1.8273
	Leu II	Val I	1.0161	1.9785	2.8796	146.3	-5.2880
	Leu I	Val II	1.0154	1.8500	2.8629	175.0	-7.7259
	Val II	Leu I	1.0166	2.0920	3.0726	161.4	-3.5561
	Pro II	Phe I	1.0931	1.4940	2.5816	172.4	-28.3932

The interatomic distance N…O varies between 3.5777 Å and 2.5816 Å, while the HB length ranges from 2.6811Å to 1.4940 Å. The geometric criteria associated with the distances and valence angle value confirmed the presence of HBs with diverse strengths. The strength of each of the discussed HBs was estimated on the basis of potential energy density (V) derived from quantum theory of atoms in molecules (QTAIM)band calculated according to the EML equation (Ref. 2):

$$E_{HB} = \frac{1}{2} \times V(r_{CP})$$

This enabled us to provide a quantitative description of the HB energy and allowed to estimate the changes associated with the diversity of the residues in the vicinity of HBs as well as the conformation of the peptide. The HB energy estimates are consistent with the structural view of these contacts. They are mostly weak and middle-strong, between -0.8 and -9.1 kcal/mol, without apparent symmetry in the cyclic structure; the weakest contacts are formed by Val I as the donor. A surprisingly strong Pro II – Phe I HB in the linear peptide is a result of the electrostatic strengthening (charge-assisted HB), since the linear structure contains charged *N*- and *C*-termini.

References:
[1] Asano, A.; Doi, M. Crystal structure of gramicidin s hydrochloride at 1.1 Å Resolution. *X-ray Structure Analysis Online* **2019**, *35* (0), 1–2. DOI: 10.2116/xraystruct.35.1
[2] Espinosa, E.; Molins, E.; Lecomte, C. Hydrogen bond strengths revealed by topological analyses of experimentally observed electron densities. *Chem. Phys. Lett.* **1998**, *285* (3–4), 170–173. DOI: 10.1016/s0009-2614(98)00036-0.

The next part of the theoretical study is devoted to the electronic structure description. We have employed QTAIM and MEP methods to gain an insight into the electron density distribution. As it is shown in Figures S35-S39 the QTAIM method detected many non-covalent interactions stabilizing the studied peptides. We cannot classify all of them as HBs; however, it is quantitative information concerning the presence of non-covalent interactions responsible for molecular features of the peptides. The contacts are formed between diverse residues, but formally within one ten-residue peptide, thus we will label the HBs as intramolecular. As we could see from the MD simulations and time-evolution analyses, it is possible that water molecules (in our case) could enter the interior of the peptide and change the HBs network forming intermolecular contacts, which is described in more detail below.

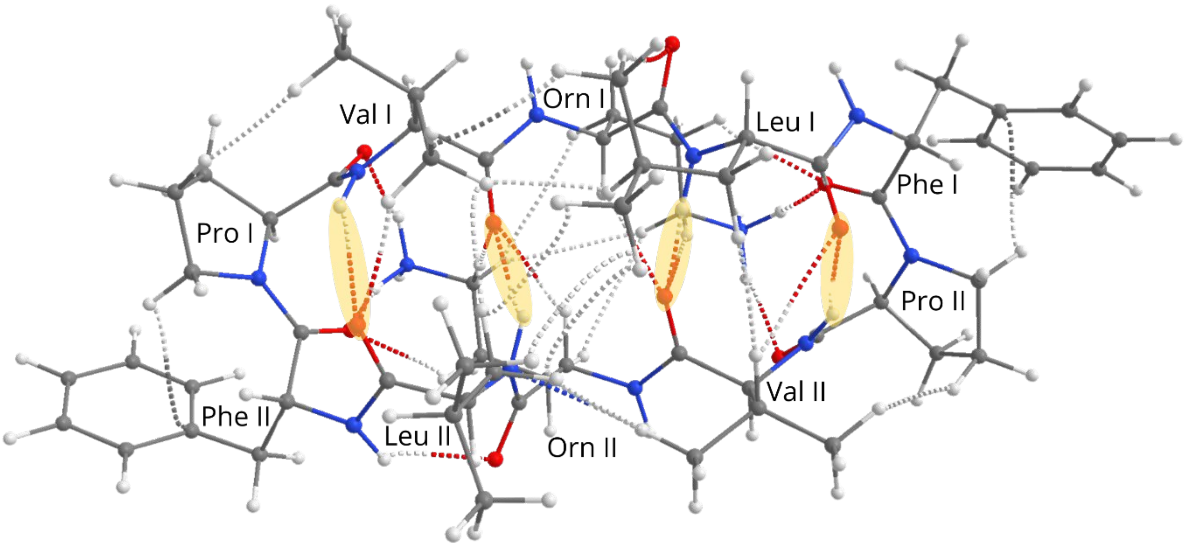

Figure S35. The top view of the GS structure calculated at the M06-2X/def2-TZVP level of theory, with the solvent reaction field (IEF-PCM, water as a solvent) indicating non-covalent interactions based on the QTAIM. The yellow ellipsoids indicate intramolecular hydrogen bonds presented in Table 3. The dotted lines indicate intramolecular interactions for which the QTAIM method detected bond critical points (BCPs).

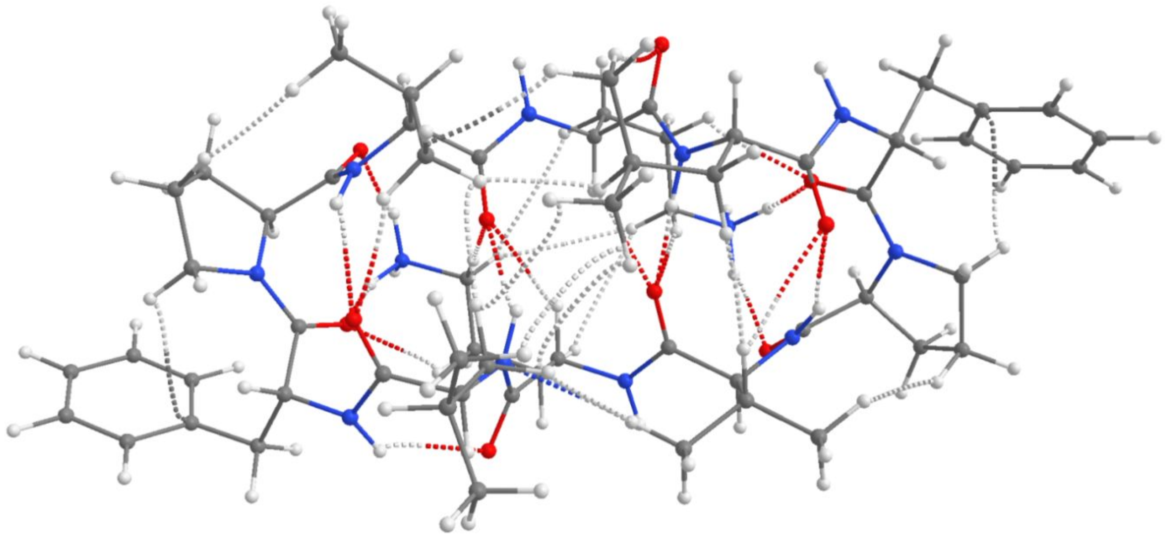

Figure S36. Top view of GS model calculated at the M06-2X/def2-TZVP level of theory with solvent reaction field (IEF-PCM, water as a solvent) indicating non-covalent interactions based on the QTAIM. The dotted lines indicate intramolecular interactions for which the QTAIM method detected bond critical points (BCPs).

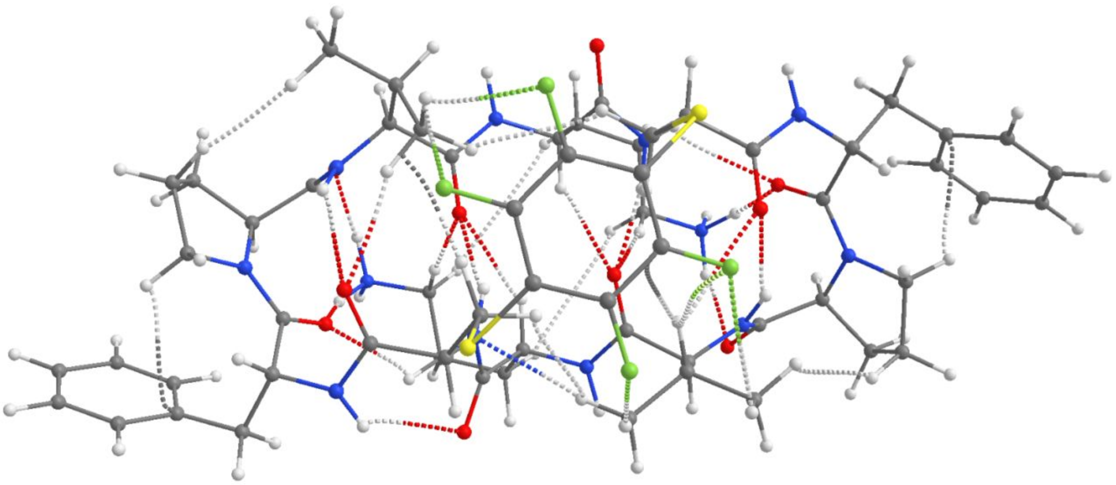

Figure S37. Top view of GS_C-FB model calculated at the M06-2X/def2-TZVP level of theory with solvent reaction field (IEF-PCM, water as a solvent) indicating non-covalent interactions based on the QTAIM. The dotted lines indicate intramolecular interactions for which the QTAIM method detected bond critical points (BCPs).

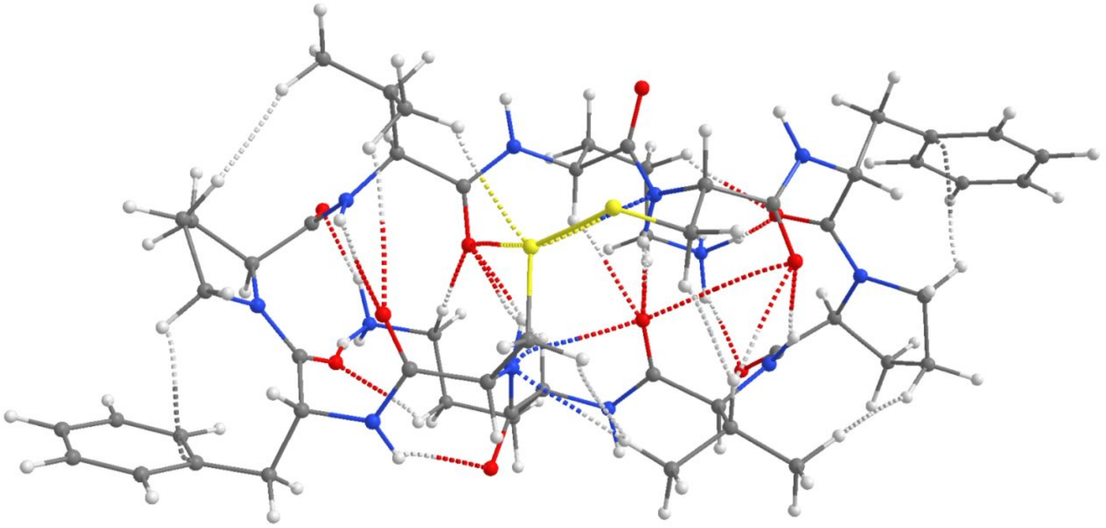

Figure S38. Top view of GS_C-SS model calculated at the M06-2X/def2-TZVP level of theory with solvent reaction field (IEF-PCM, water as a solvent) indicating non-covalent interactions based on the QTAIM. The dotted lines indicate intramolecular interactions for which the QTAIM method detected bond critical points (BCPs).

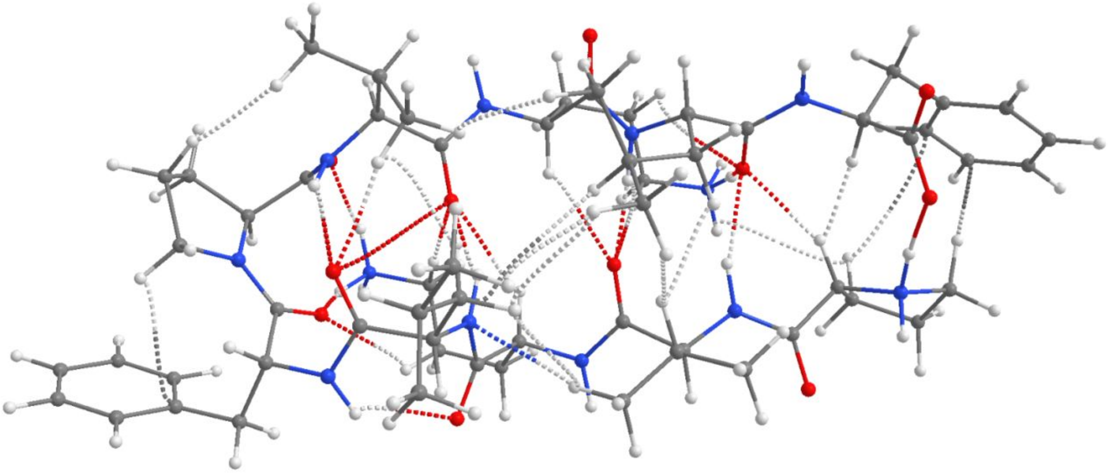

Figure S39. Top view of GS-L model calculated at the M06-2X/def2-TZVP level of theory with solvent reaction field (IEF-PCM, water as a solvent) indicating non-covalent interactions based on the QTAIM. The dotted lines indicate intramolecular interactions for which the QTAIM method detected bond critical points (BCPs).

The last analysis based on static models for the GS and its analogs was related to the atomic charge distribution and its potential effect on the biological target interactions. MEP were prepared based on static models obtained at the M06-2X/def2-TZVP level of theory and with the presence of the IEF-PCM model (water was used to reproduce the polar environment influence on molecular properties). The blue isosurfaces indicate the positive MEP while the red isosurfaces showed places in the peptides with negative MEP. From the data shown in Figure S40, we can see that the positive MEP dominates in the studied structures. Moreover, it is distributed rather evenly on the surfaces of the molecules. However, the isosurfaces with negative MEP are less pronounced, which could suggest that the dominant interaction should be with positively charged regions. They are distributed and cover also the ornithine side chains, responsible for electrostatic interactions of the peptide.

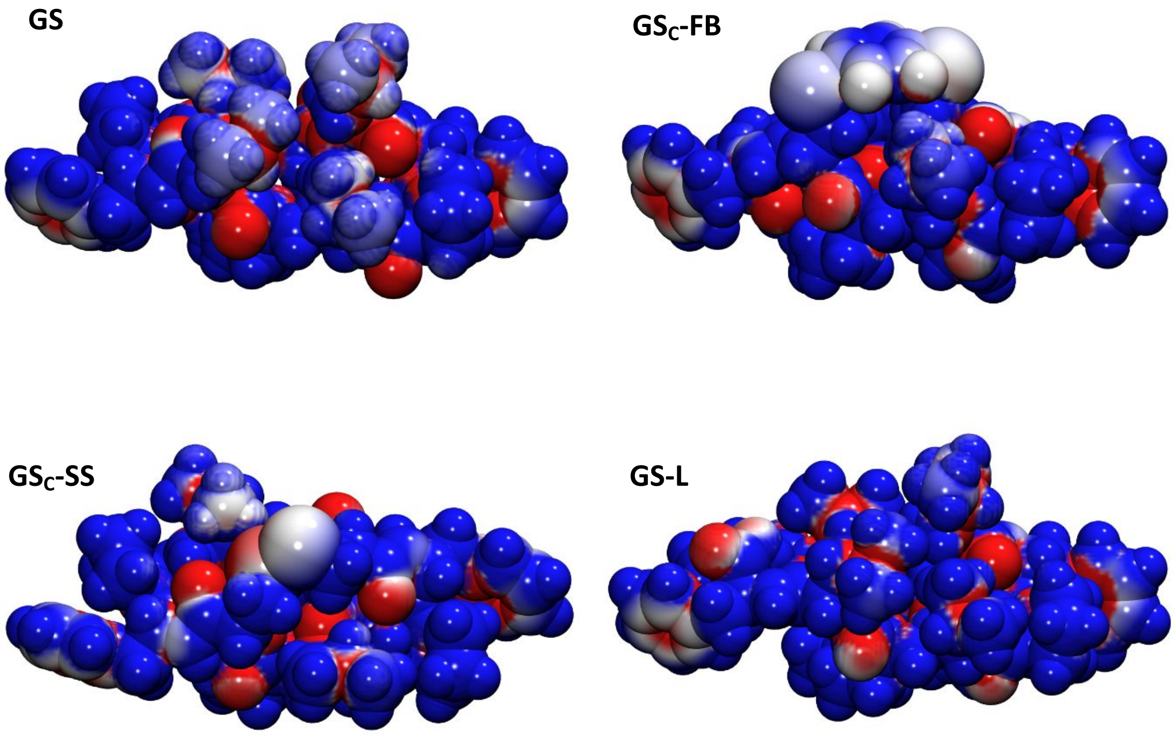

Figure S40. Molecular Electrostatic Potential (MEP) surfaces of GS, GS_C-FB, GS_C-SS, and GS-L. Blue color indicates the positive MEP while the red – negative one.

Molecular Dynamics of GS and its Analogs in Aqueous Solution

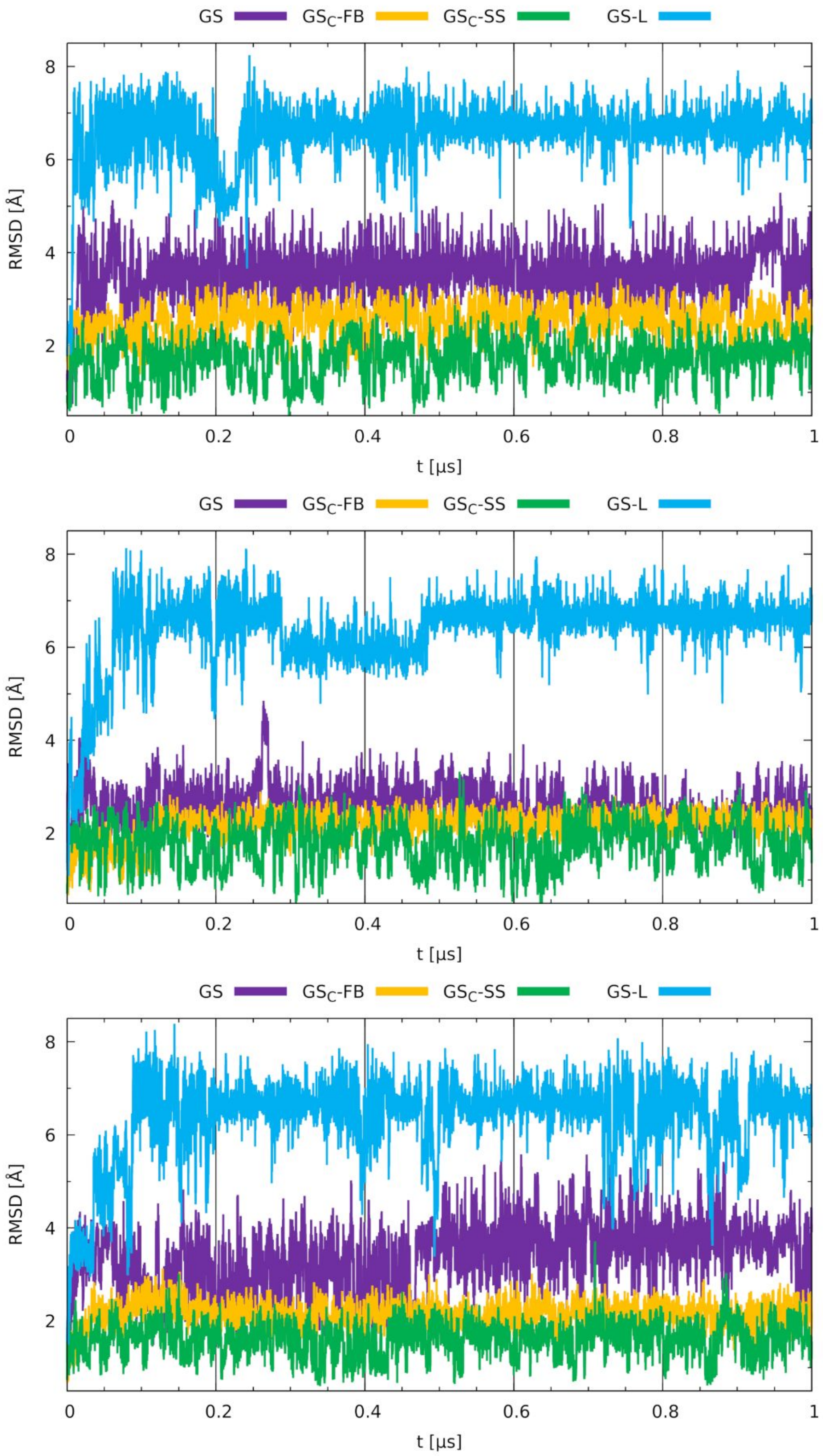

Figure S41. Root Mean Square Deviation (RMSD) calculated for GS, GS-L, GS_C-FB, and GS_C-FB (three MD runs for the set of the studied peptides) in water.

The RMSF analysis (see Figure S43) underlines perfectly the differences between the stiffness of gramicidin S and its stapled or linear variants. Fluctuations of the $C_α$ atoms are strongly marked for the GS-L, while the dynamics of motions within the stapled peptides are markedly inhibited. The GS_C-SS is quite evenly stable, while one of the two loops of the GS_C-FB variant formed by stapling is more dynamic than the other loop. This underlines the fact that the perfluoroaryl stapling leaves more room for the peptide flexibility than the disulfide bridge.

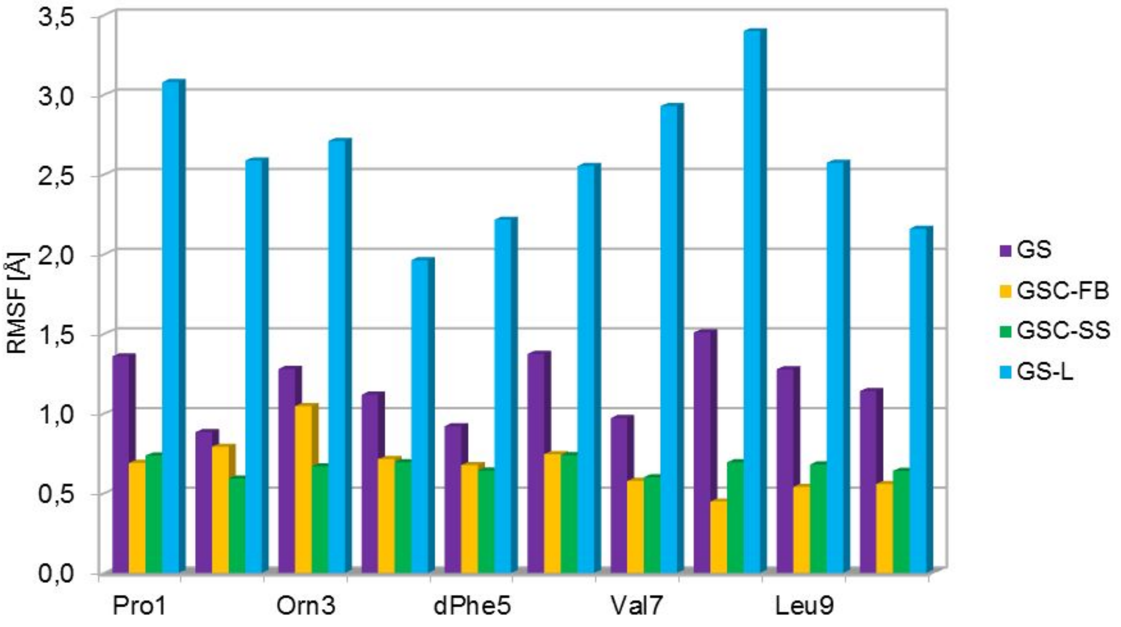

Figure S42. Root Mean Square Fluctuation (RMSF) calculated for α-carbon atoms of GS, GS-L, GS_C-FB, and GS_C-FB in water.

Next analysis was associated with the surface area of a peptide that is accessible to a solvent (SASA) and the part of SASA restricted to the polar atoms (oxygen, nitrogen and -OH or -NH hydrogen atoms) – Polar Surface Area (PSA). As it is shown in Figure S44 the lowest SASA values, close to 1100 Å^2, were obtained for GS_C-SS analog. Similar solvent accessibility is exhibited by GS and GS_C-FB analogs. The obtained values do not differ significantly. They are in the range between 1200 – 1250 Å^2. As it could be expected, the largest SASA values (range between 1250 – 1300 Å^2) were obtained for the GS-L analog. Again, the analysis confirmed that the cyclic analogs are less accessible for the solvent. Additional insight is obtained via investigation of PSA, the part of SASA covering the polar atoms – see Figure S45. Interestingly, the cyclic peptides are very similar in their PSA values throughout the simulation, including the most compact GS_C-SS peptide (with the lowest overall SASA parameter). This increased PSA/SASA ratio suggests that the exposed surface of this particular peptide is more

hydrophilic than the other cyclic analogs, reducing its ability to permeate lipophilic membranes. The linear GS-L peptide is also more hydrophilic due to the appearance of terminal zwitterionic groups.

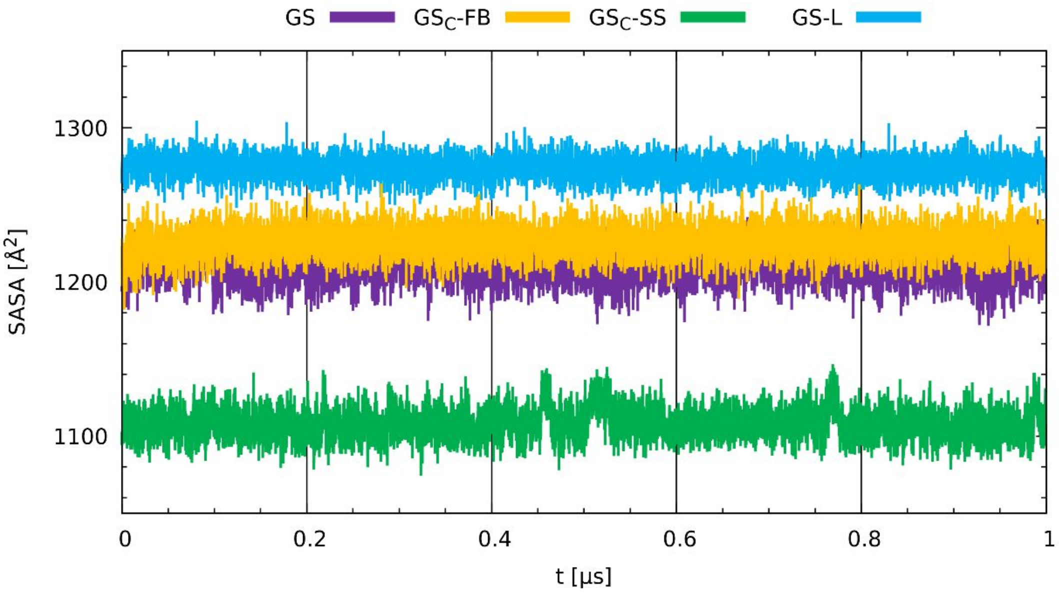

Figure S43. Solvent-Accessible Surface Area (SASA) calculated for GS, GS-L, GS_C-FB, and GS_C-FB in water.

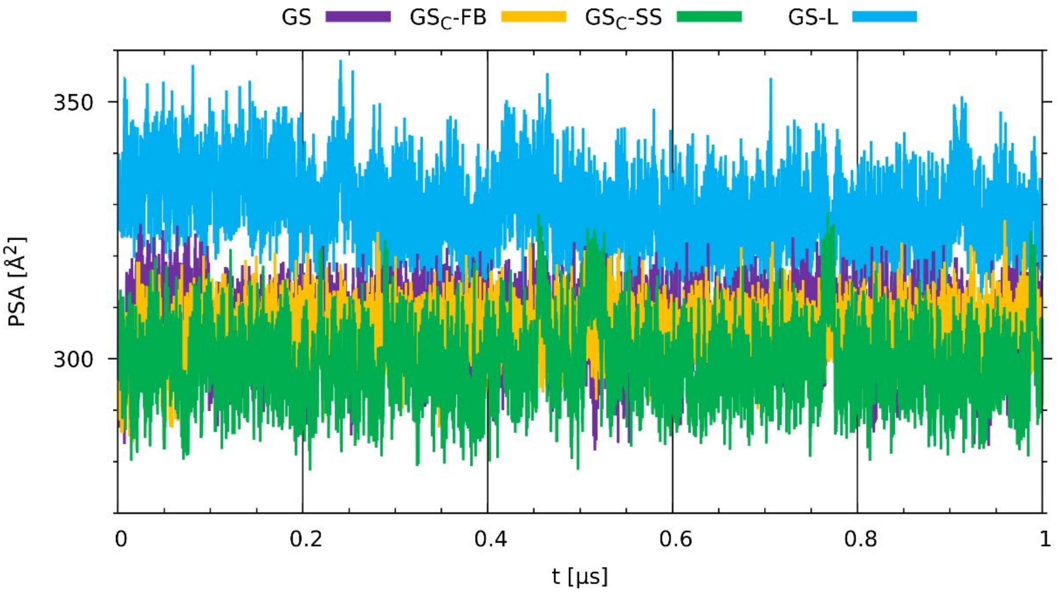

Figure S44. Time-evolution of the polar surface area (PSA) obtained for GS, GS-L, GS_C-FB, and GS_C-SS in water.

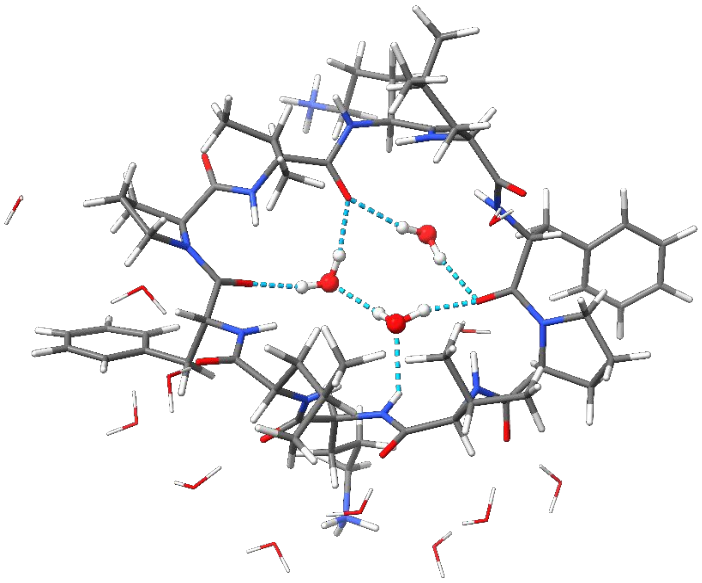

Figure S45. Frame from the MD simulations showing the expanded GS structure. The dotted cyan lines indicate the presence of hydrogen bonds formed between GS and water molecules (rendered as ball-and-stick model). Color coding: grey – carbon, blue – nitrogen, red – oxygen, and white – hydrogen.

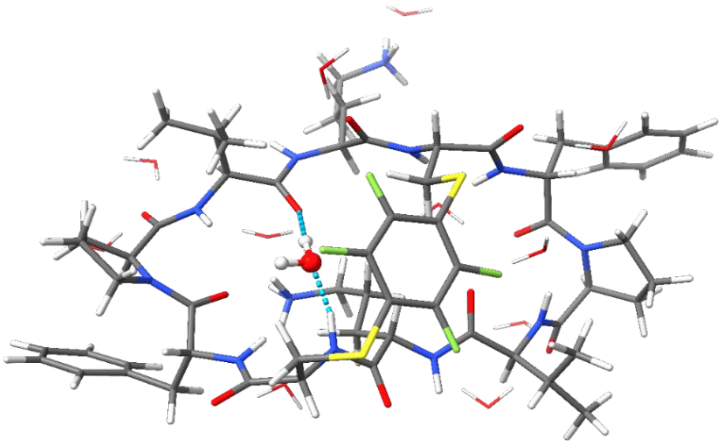

Figure S46. Frame from the MD simulations showing the GS_C-FB structure. The dotted cyan lines indicate the presence of hydrogen bonds formed between GS_C-FB and water molecule (rendered as ball-and-stick model). Color coding: grey – carbon, blue – nitrogen, red – oxygen, yellow – sulfur, green – fluorine, and white – hydrogen.
